# Supplementary material for: New Cardenolides from Biotransformation of Gitoxigenin by the Endophytic Fungus Alternaria eureka 1E1BL1: Characterization and Cytotoxic Activities
Source: Molecules. 2021 May 19;26(10):3030. doi: 10.3390/molecules26103030 (PMC8161373; doi:10.3390/molecules26103030)
Supplement: Supplementary file 1 [file molecules-26-03030-s001.zip › molecules-1161544-supplementary.pdf]

Supporting information

New Cardenolides from Biotransformation of Gitoxigenin by the Endophytic Fungus *Alternaria eureka* 1E1BL1: Characterization and Cytotoxic Activities

Erdal Bedir [1,*], Çiğdem Karakoyun [2,*], Gamze Doğan [1], Gülten Kuru [1], Melis Küçüksolak [1], Hasan Yusufoğlu [3]

[1] Department of Bioengineering, Faculty of Engineering, İzmir Institute of Technology, 35430 Urla-İzmir, Turkey
[2] Department of Pharmacognosy, Faculty of Pharmacy, Ege University, 35100 Bornova-İzmir, Turkey
[3] Department of Pharmacognosy, College of Pharmacy, Prince Sattam Bin Abdulaziz University, 11942 Al-Kharj, Saudi Arabia

* Correspondence: erdalbedir@iyte.edu.tr and cigdem.karakoyun@ege.edu.tr

Index/List of figures, tables and chromatograms:

Figure S1. Negative-ion mode HR-ESI-MS spectrum of substrate gitoxigenin.................................... 5
Figure S2. Positive-ion mode HR-ESI-MS spectrum of substrate gitoxigenin 6
Figure S3. FT-IR Spectrum of substrate gitoxigenin... 7
Figure S4. ^{1}H NMR Spectrum of substrate gitoxigenin 8
Figure S5. ^{13}C NMR Spectrum of substrate gitoxigenin 9
Figure S6. COSY Spectrum of substrate gitoxigenin.. 10
Figure S7. HSQC Spectrum of substrate gitoxigenin.. 11
Figure S8. HMBC Spectrum of substrate gitoxigenin.. 12
Figure S9. NOESY Spectrum of substrate gitoxigenin 13
Figure S10. Negative-ion mode HR-ESI-MS spectrum of 7(β)-hydroxy-3-oxo-gitoxigenin (**compound 1**) 14
Figure S11. Positive-ion mode HR-ESI-MS spectrum of 7(β)-hydroxy-3-oxo-gitoxigenin (**compound 1**)... 15
Figure S12. FT-IR Spectrum of 7(β)-hydroxy-3-oxo-gitoxigenin (**compound 1**).............................. 16
Figure S13. ^{1}H NMR Spectrum of 7(β)-hydroxy-3-oxo-gitoxigenin (**compound 1**).......................... 17
Figure S14. ^{13}C NMR Spectrum of 7(β)-hydroxy-3-oxo-gitoxigenin (**compound 1**)......................... 18
Figure S15. COSY Spectrum of 7(β)-hydroxy-3-oxo-gitoxigenin (**compound 1**) 19
Figure S16. HSQC Spectrum of 7(β)-hydroxy-3-oxo-gitoxigenin (**compound 1**) 20
Figure S17. HMBC Spectrum of 7(β)-hydroxy-3-oxo-gitoxigenin (**compound 1**) 21
Figure S18. NOESY Spectrum of 7(β)-hydroxy-3-oxo-gitoxigenin (**compound 1**)........................... 22
Figure S19. Negative-ion mode HR-ESI-MS spectrum of 3-oxo-gitoxigenin (**compound 2**)............ 23
Figure S20. Positive-ion mode HR-ESI-MS spectrum of 3-oxo-gitoxigenin (**compound 2**)............. 24
Figure S21. FT-IR Spectrum of 3-oxo-gitoxigenin (**compound 2**) 25
Figure S22. ^{1}H NMR Spectrum of 3-oxo-gitoxigenin (**compound 2**) 26
Figure S23. ^{13}C NMR Spectrum of 3-oxo-gitoxigenin (**compound 2**) 27
Figure S24. COSY Spectrum of 3-oxo-gitoxigenin (**compound 2**)... 28
Figure S25. HSQC Spectrum of 3-oxo-gitoxigenin (**compound 2**)... 29
Figure S26. HMBC Spectrum of 3-oxo-gitoxigenin (**compound 2**).. 30
Figure S27. NOESY Spectrum of 3-oxo-gitoxigenin (**compound 2**) 31
Figure S28. Negative-ion mode HR-ESI-MS spectrum of 7(β),12(β)-dihdyroxy-3-oxo-gitoxigenin (**compound 3**) 32
Figure S29. Positive-ion mode HR-ESI-MS spectrum of 7(β),12(β)-dihdyroxy-3-oxo-gitoxigenin (**compound 3**) 33
Figure S30. FT-IR Spectrum of 7(β),12(β)-dihdyroxy-3-oxo-gitoxigenin (**compound 3**) 34
Figure S31. ^{1}H NMR Spectrum of 7(β),12(β)-dihdyroxy-3-oxo-gitoxigenin (**compound 3**)............. 35
Figure S32. ^{13}C NMR Spectrum of 7(β),12(β)-dihdyroxy-3-oxo-gitoxigenin (**compound 3**)............ 36
Figure S33. COSY Spectrum of 7(β),12(β)-dihdyroxy-3-oxo-gitoxigenin (**compound 3**) 37
Figure S34. HSQC Spectrum of 7(β),12(β)-dihdyroxy-3-oxo-gitoxigenin (**compound 3**) 38
Figure S35. HMBC Spectrum of 7(β),12(β)-dihdyroxy-3-oxo-gitoxigenin (**compound 3**) 39
Figure S36. NOESY Spectrum of 7(β),12(β)-dihdyroxy-3-oxo-gitoxigenin (**compound 3**)............... 40
Figure S37. Negative-ion mode HR-ESI-MS spectrum of 3-dimethylacetal-7(β)-hydroxygitoxigenin (**compound 4**) 41
Figure S38. Positive-ion mode HR-ESI-MS spectrum of 3-dimethylacetal-7(β)-hydroxygitoxigenin (**compound 4**) 42
Figure S39. FT-IR spectrum of 3-dimethylacetal-7(β)-hydroxygitoxigenin (**compound 4**) 43
Figure S40. ^{1}H NMR Spectrum of 3-dimethylacetal-7(β)-hydroxygitoxigenin (**compound 4**) 44
Figure S41. ^{13}C NMR Spectrum of 3-dimethylacetal-7(*β*)-hydroxygitoxigenin (**compound 4**) 45
Figure S42. COSY Spectrum of 3-dimethylacetal-7(β)-hydroxygitoxigenin (**compound 4**)............. 46
Figure S43. HSQC Spectrum of 3-dimethylacetal-7(β)-hydroxygitoxigenin (**compound 4**)............. 47

Figure S44. HMBC Spectrum of 3-dimethylacetal-7(β)-hydroxygitoxigenin (**compound 4**)............ 48
Figure S45. NOESY Spectrum of 3-dimethylacetal-7(β)-hydroxygitoxigenin (**compound 4**) 49
Figure S46. Negative-ion mode HR-ESI-MS spectrum of 3-epi-gitoxigenin (**compound 5**)............. 50
Figure S47. Positive-ion mode HR-ESI-MS spectrum of 3-epi-gitoxigenin (**compound 5**) 51
Figure S48. FT-IR Spectrum of 3-epi-gitoxigenin (**compound 5**)... 52
Figure S49. ^{1}H NMR Spectrum of 3-epi-gitoxigenin (**compound 5**) 53
Figure S50. ^{13}C NMR Spectrum of 3-epi-gitoxigenin (**compound 5**) 54
Figure S51. COSY Spectrum of 3-epi-gitoxigenin (**compound 5**).. 55
Figure S52. HSQC Spectrum of 3-epi-gitoxigenin (**compound 5**).. 56
Figure S53. HMBC Spectrum of 3-epi-gitoxigenin (**compound 5**)... 57
Figure S54. NOESY Spectrum of 3-epi-gitoxigenin (**compound 5**) 58
Figure S55. Negative-ion mode HR-ESI-MS spectrum of 7(β)-hydroxy-3-epi-gitoxigenin (**compound 6**)... 59
Figure S56. Positive-ion mode HR-ESI-MS spectrum of 7(β)-hydroxy-3-epi-gitoxigenin (**compound 6**)... 60
Figure S57: FT-IR Spectrum of 7(β)-hydroxy-3-epi-gitoxigenin (**compound 6**)................................ 61
Figure S58. ^{1}H NMR Spectrum of 7(β)-hydroxy-3-epi-gitoxigenin (**compound 6**)........................... 62
Figure S59. ^{13}C NMR Spectrum of 7(β)-hydroxy-3-epi-gitoxigenin (**compound 6**).......................... 63
Figure S60. COSY Spectrum of 7(β)-hydroxy-3-epi-gitoxigenin (**compound 6**) 64
Figure S61. HSQC Spectrum of 7(β)-hydroxy-3-epi-gitoxigenin (**compound 6**) 65
Figure S62. HMBC Spectrum of 7(β)-hydroxy-3-epi-gitoxigenin (**compound 6**) 66
Figure S63. NOESY Spectrum of 7(β)-hydroxy-3-epi-gitoxigenin (**compound 6**)............................. 67
Figure S64. Negative-ion mode HR-ESI-MS spectrum of 3-oxo-diginatigenin (**compound 7**).......... 68
Figure S65. Positive-ion mode HR-ESI-MS spectrum of 3-oxo-diginatigenin (**compound 7**) 69
Figure S66. FT-IR Spectrum of 3-oxo-diginatigenin (**compound 7**).. 70
Figure S67. ^{1}H NMR Spectrum of 3-oxo-diginatigenin (**compound 7**) 71
Figure S68. ^{13}C NMR Spectrum of 3-oxo-diginatigenin (**compound 7**) 72
Figure S69. COSY Spectrum of 3-oxo-diginatigenin (**compound 7**).. 73
Figure S70. HSQC Spectrum of 3-oxo-diginatigenin (**compound 7**).. 74
Figure S71. HMBC Spectrum of 3-oxo-diginatigenin (**compound 7**)... 75
Figure S72. NOESY Spectrum of 3-oxo-diginatigenin (**compound 7**) 76
Figure S73. Negative-ion mode HR-ESI-MS spectrum of 3-epi-diginatigenin (**compound 8**)........... 77
Figure S74. Positive-ion mode HR-ESI-MS spectrum of 3-epi-diginatigenin (**compound 8**) 78
Figure S75. FT-IR Spectrum of 3-epi-diginatigenin (**compound 8**).. 79
Figure S76. ^{1}H NMR Spectrum of 3-epi-diginatigenin (**compound 8**) 80
Figure S77. ^{13}C NMR Spectrum of 3-epi-diginatigenin (**compound 8**) 81
Figure S78. COSY Spectrum of 3-epi-diginatigenin (**compound 8**).. 82
Figure S79. HSQC Spectrum of 3-epi-diginatigenin (**compound 8**).. 83
Figure S80. HMBC Spectrum of 3-epi-diginatigenin (**compound 8**)... 84
Figure S81. NOESY Spectrum of 3-epi-diginatigenin (**compound 8**) 85

Table S1: HPLC Method for qualitative analysis of selected metabolites (**4**, **5** and **6**) and EtOAc extract of biotransformation broth.. 86

Chromatogram S1: HPLC chromatograms of **a)** EtOAc extract, **b) Compound 4** (retention time: 10.8 min), **c) Compound 5** (retention time: 9.6 min), **d) Compound 6** (retention time: 8.9 min) and **e)** comparison of the EtOAc extract (blue line) with **Compound 4** (red line), **Compound 5** (green line), **Compound 6** (pink line). 87
Chromatogram S2: LC-Q-ToF total ion chromatograms of the EtOAc extracts showing different biotransformation time points (Day 6, 10, 14, 18 and 21) (in negative mode). 88

Chromatogram S3 : LC-Q-ToF extracted ion chromatograms of the selected compounds (Gitoxigenin, **1**, **2**, **4** and **5**) (in negative mode); **a)** Gitoxigenin (retention time: 10.85 min), **b) Compound 1** (retention time: 11.19 min), **c) Compound 2** (retention time: 12.02 min), **d) Compound 4** (retention time: 13.21 min), **e) Compound 5** (retention time: 11.65 min) and **f)** overlapped chromatograms 89
Chromatogram S4: LC-Q-ToF total and extracted ion chromatograms of gitoxigenin together with its mass spectrum (in negative mode). 90
Chromatogram S5: LC-Q-ToF extracted ion chromatograms of gitoxigenin for different biotransformation time points (Day 6, 10, 14, 18 and 21), displaying that gitoxigenin was consumed before Day 14 (in negative mode). 91
Chromatogram S6: LC-Q-ToF total and extracted ion chromatograms of **1** together with its mass spectrum (in negative mode). 92
Chromatogram S7: LC-Q-ToF extracted ion chromatograms of **1** for different biotransformation time points (Day 6, 10, 14, 18 and 21), displaying that compound **1** was present and accumulated from Day 6 to Day 21 (in negative mode). 93
Chromatogram S8: LC-Q-ToF total and extracted ion chromatograms of **2** together with its mass spectrum (in negative mode). 94
Chromatogram S9: LC-Q-ToF extracted ion chromatograms of **2** for different biotransformation time points (Day 6, 10, 14, 18 and 21), displaying that compound **2** was present and accumulated from Day 6 to Day 21 (in negative mode). 95
Chromatogram S10: LC-Q-ToF total and extracted ion chromatograms of **4** together with its mass spectrum (in negative mode). 96
Chromatogram S11: LC-Q-ToF extracted ion chromatograms of **4** for different biotransformation time points (Day 6, 10, 14, 18 and 21), displaying that compound **4** was produced and accumulated in last three biotransformation time points (Day 14, 18 and 21) (in negative mode). 97
Chromatogram S12: LC-Q-ToF total and extracted ion chromatograms of **5** together with its mass spectrum (in negative mode). 98
Chromatogram S13: LC-Q-ToF extracted ion chromatograms of **5** for different biotransformation time points (Day 6, 10, 14, 18 and 21), displaying that compound **5** was produced in the last three biotransformation time points (Day 14, 18 and 21) (in negative mode). 99

Scheme S1: Isolation pattern for biotransformation products of gitoxigenin 100
Scheme S2: Isolation pattern for biotransformation products of gitoxigenin 101
Table S 2: LC-MS method for qualitative analysis of the selected metabolites (**1, 2, 4**, **5**), gitoxigenin, and the EtOAc extracts of biotransformation broth. 102

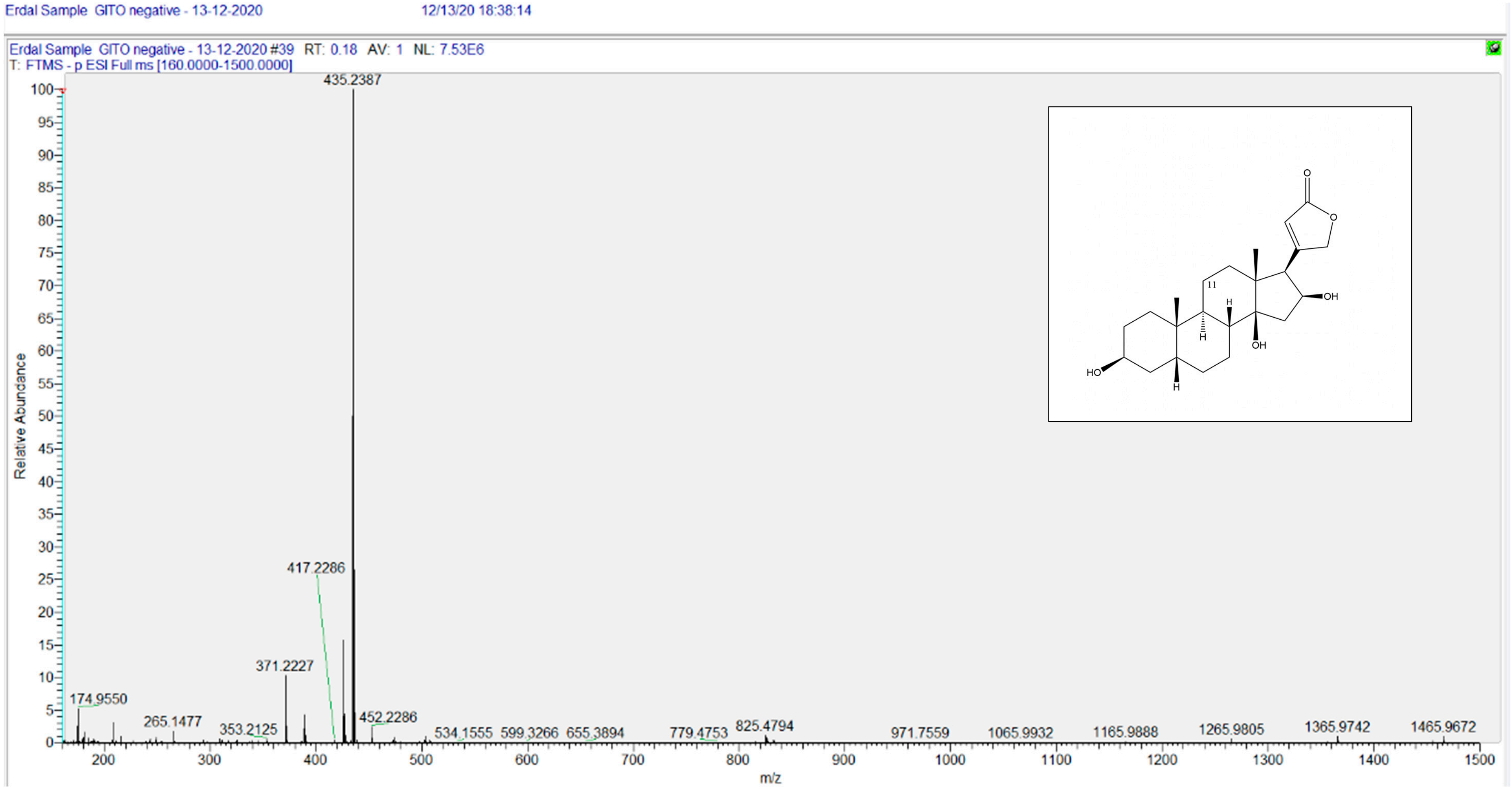

Figure S1. Negative-ion mode HR-ESI-MS spectrum of substrate gitoxigenin

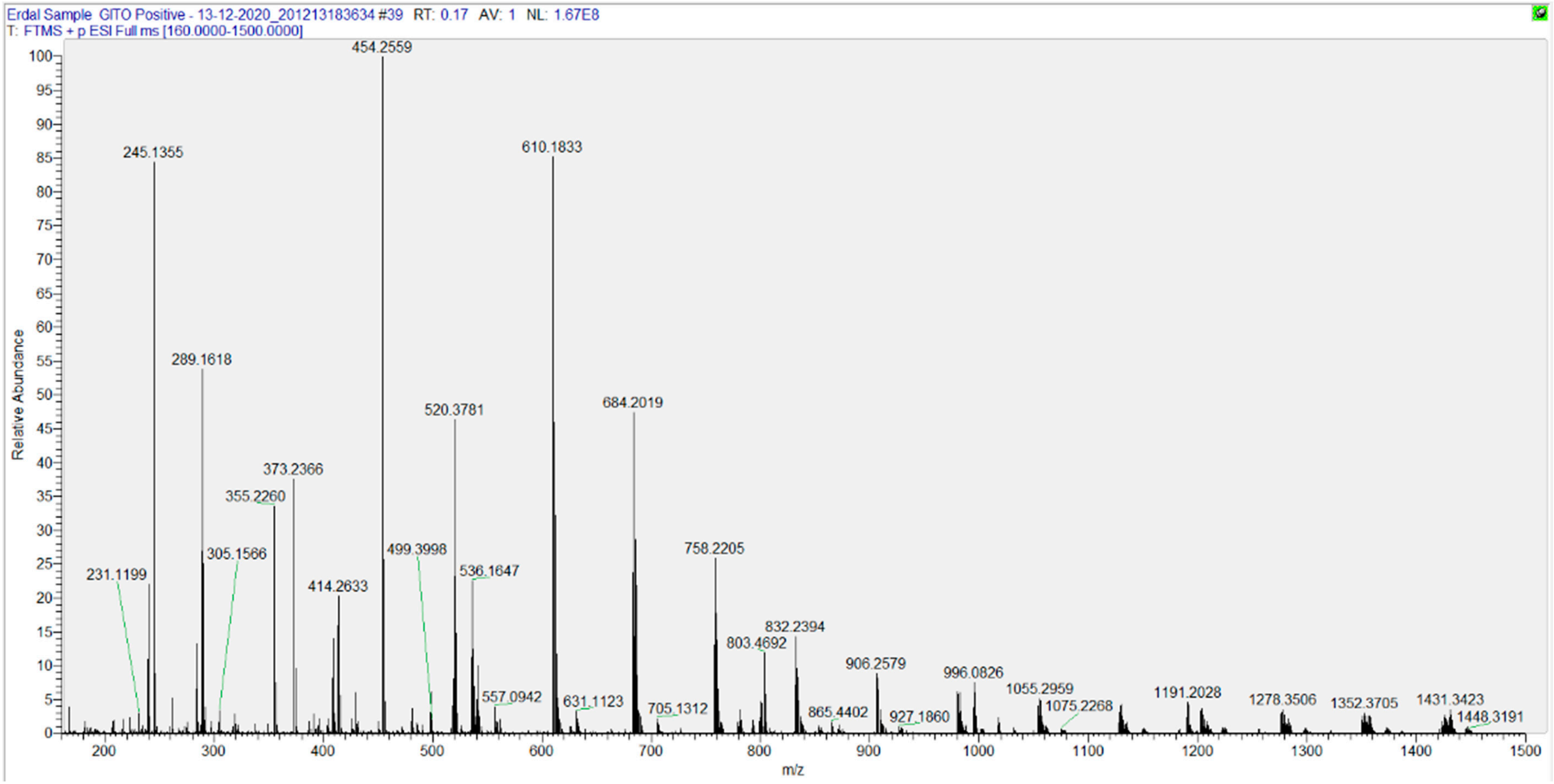

Figure S2. Positive-ion mode HR-ESI-MS spectrum of substrate gitoxigenin

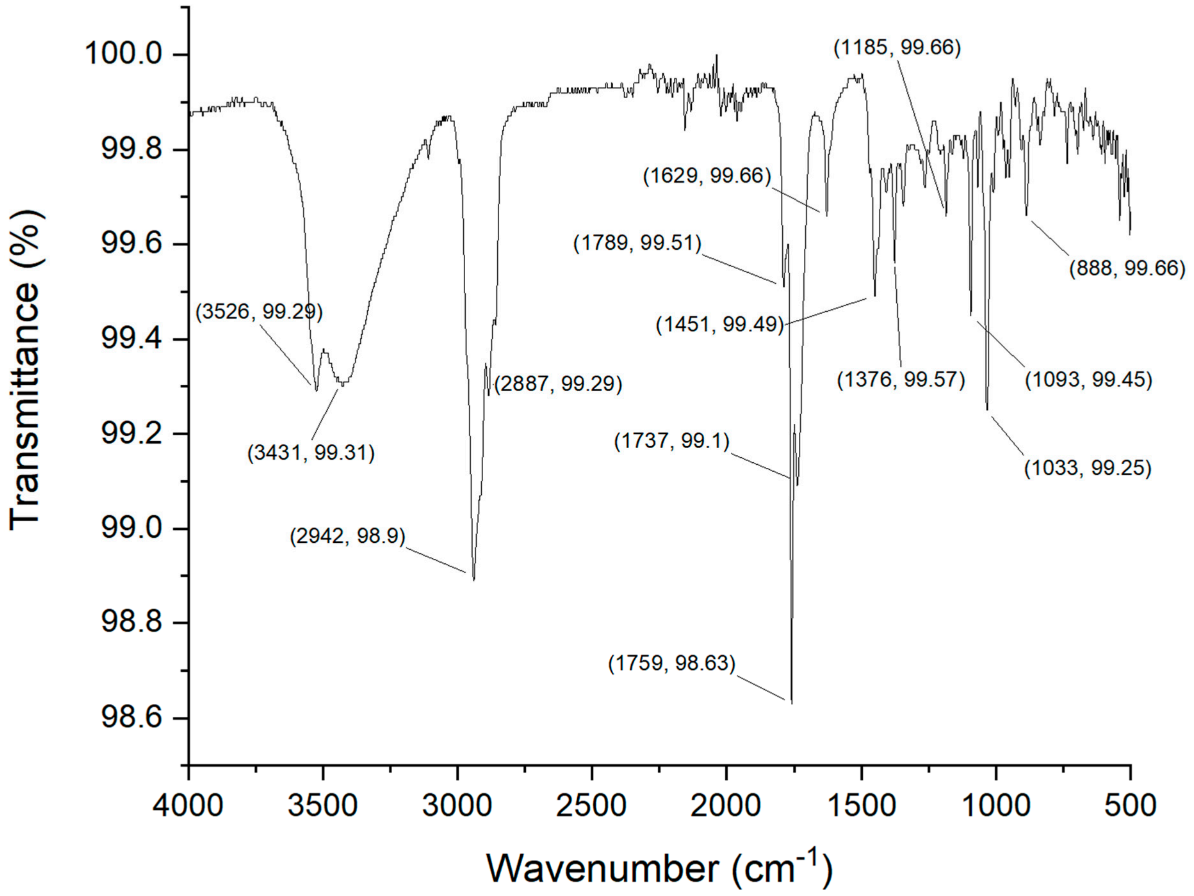

Figure S3. FT-IR Spectrum of substrate gitoxigenin

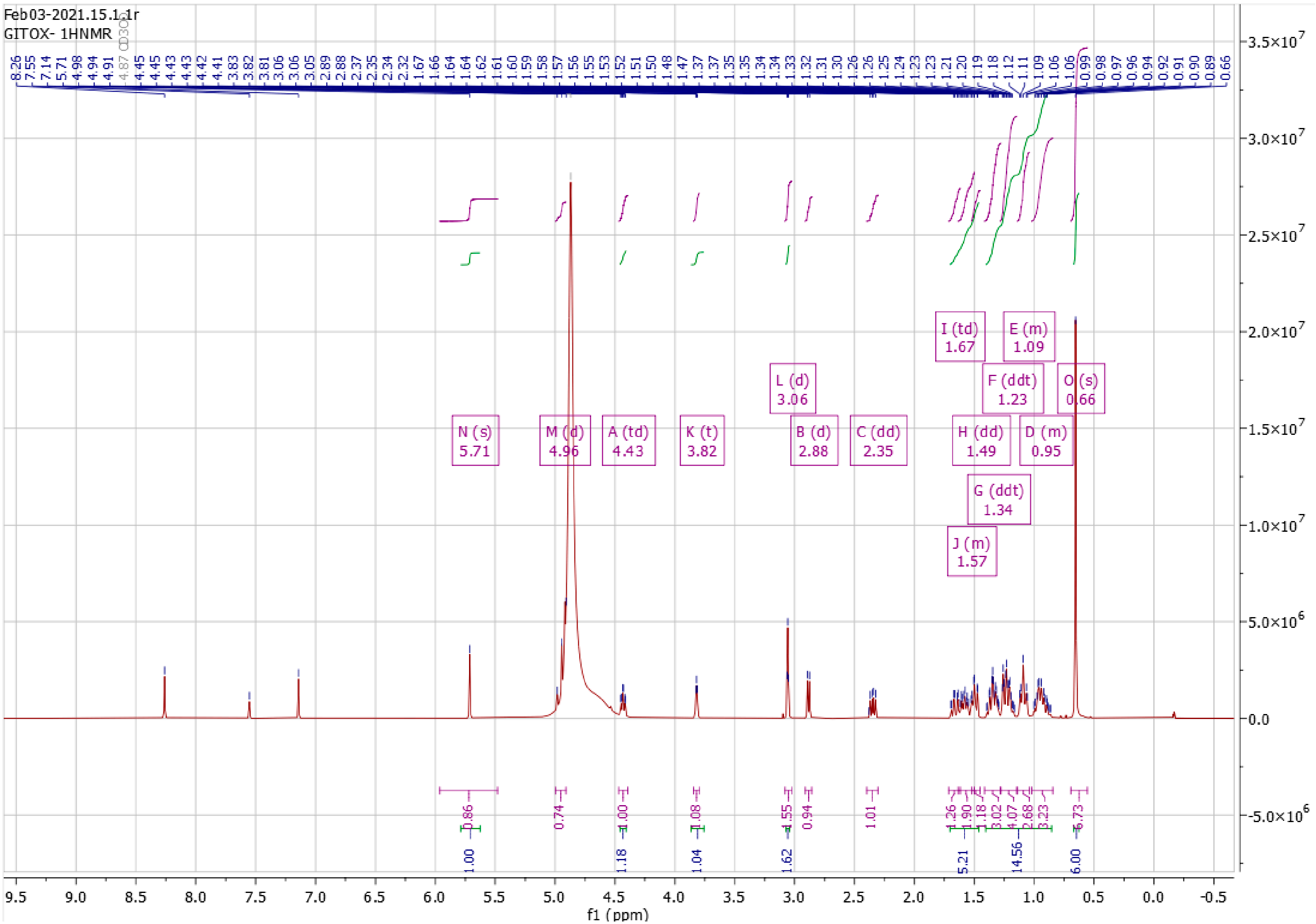

Figure S4. ^{1}H NMR Spectrum of substrate gitoxigenin

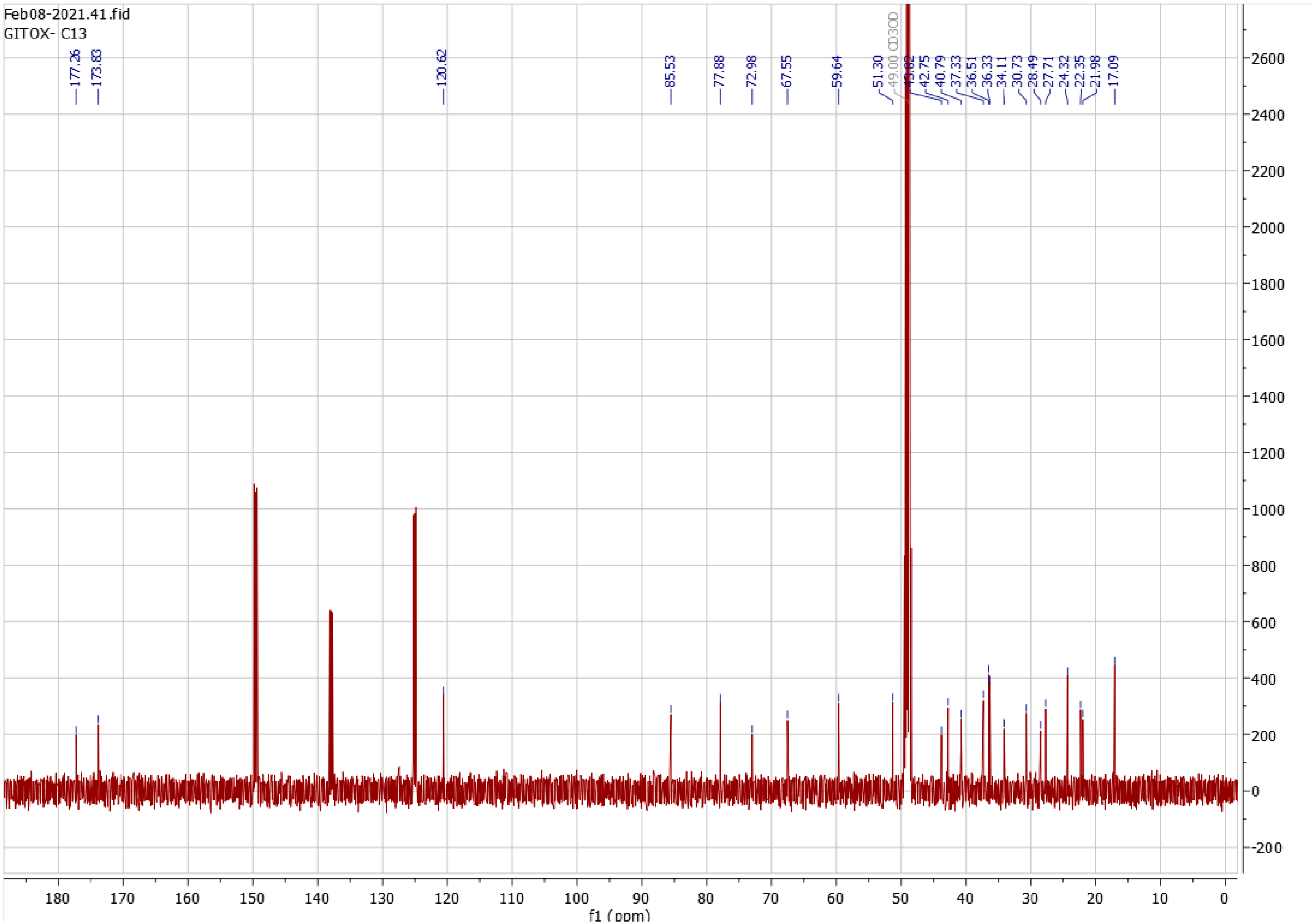

Figure S5. ^{13}C NMR Spectrum of substrate gitoxigenin

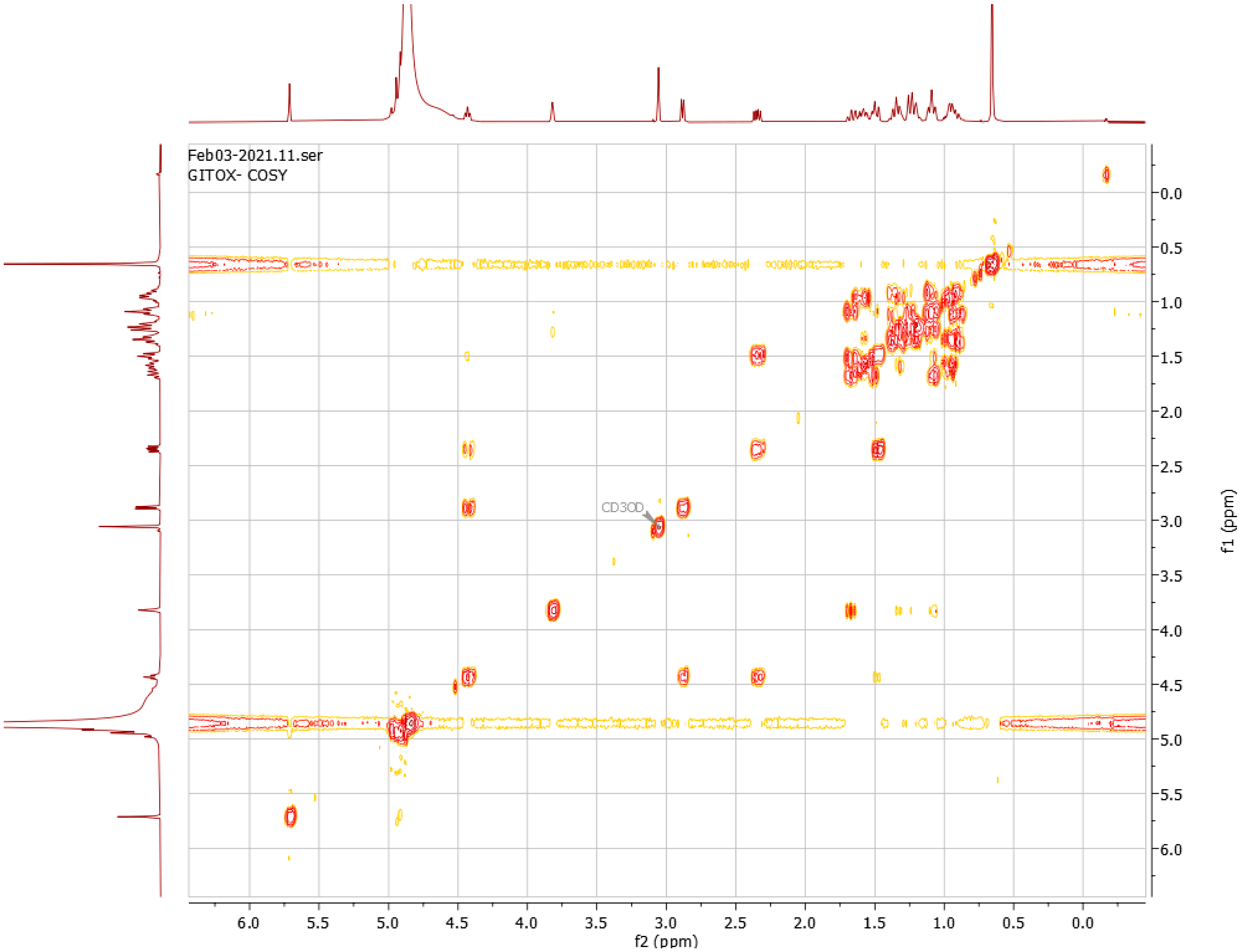

Figure S6. COSY Spectrum of substrate gitoxigenin

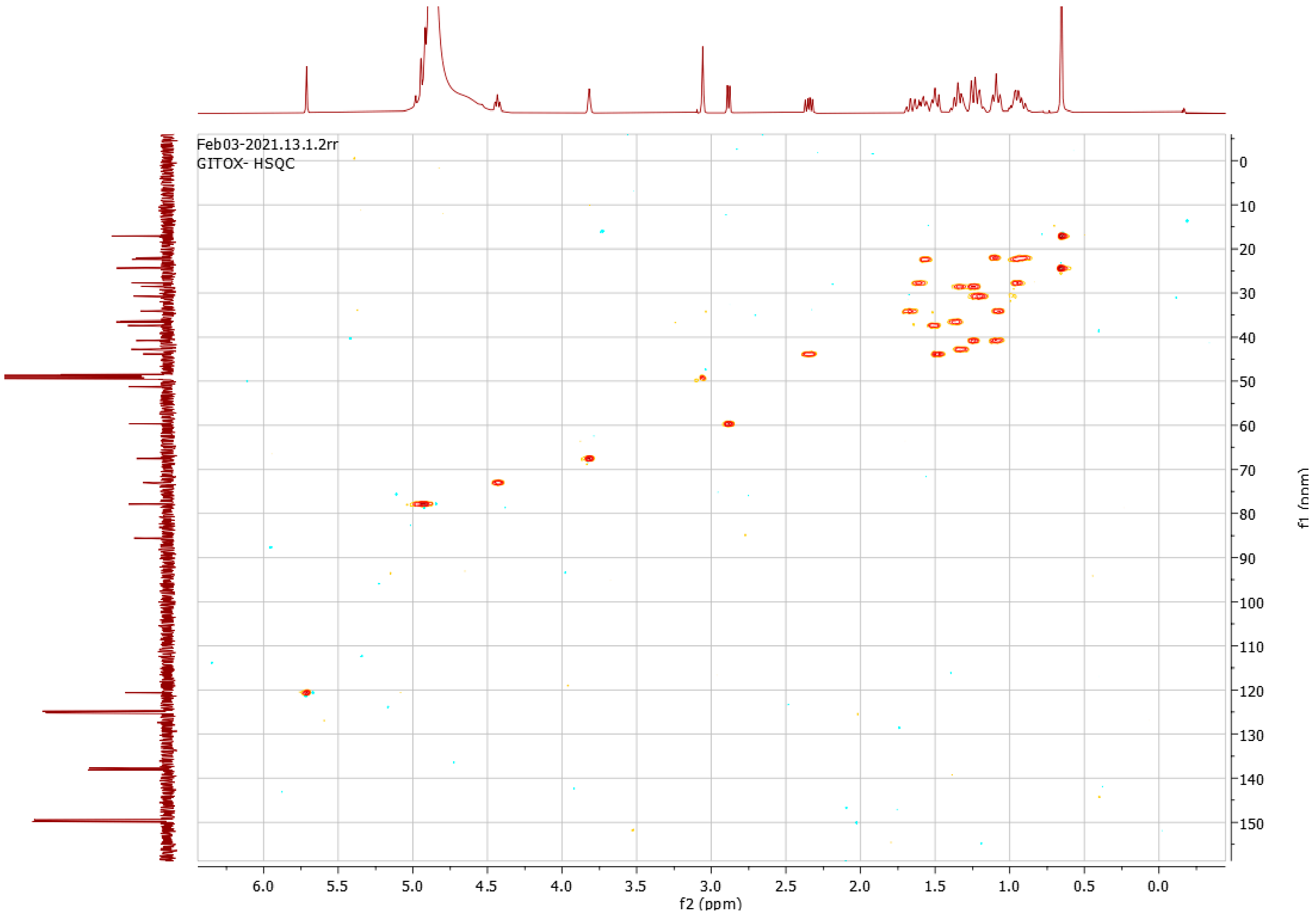

Figure S7. HSQC Spectrum of substrate gitoxigenin

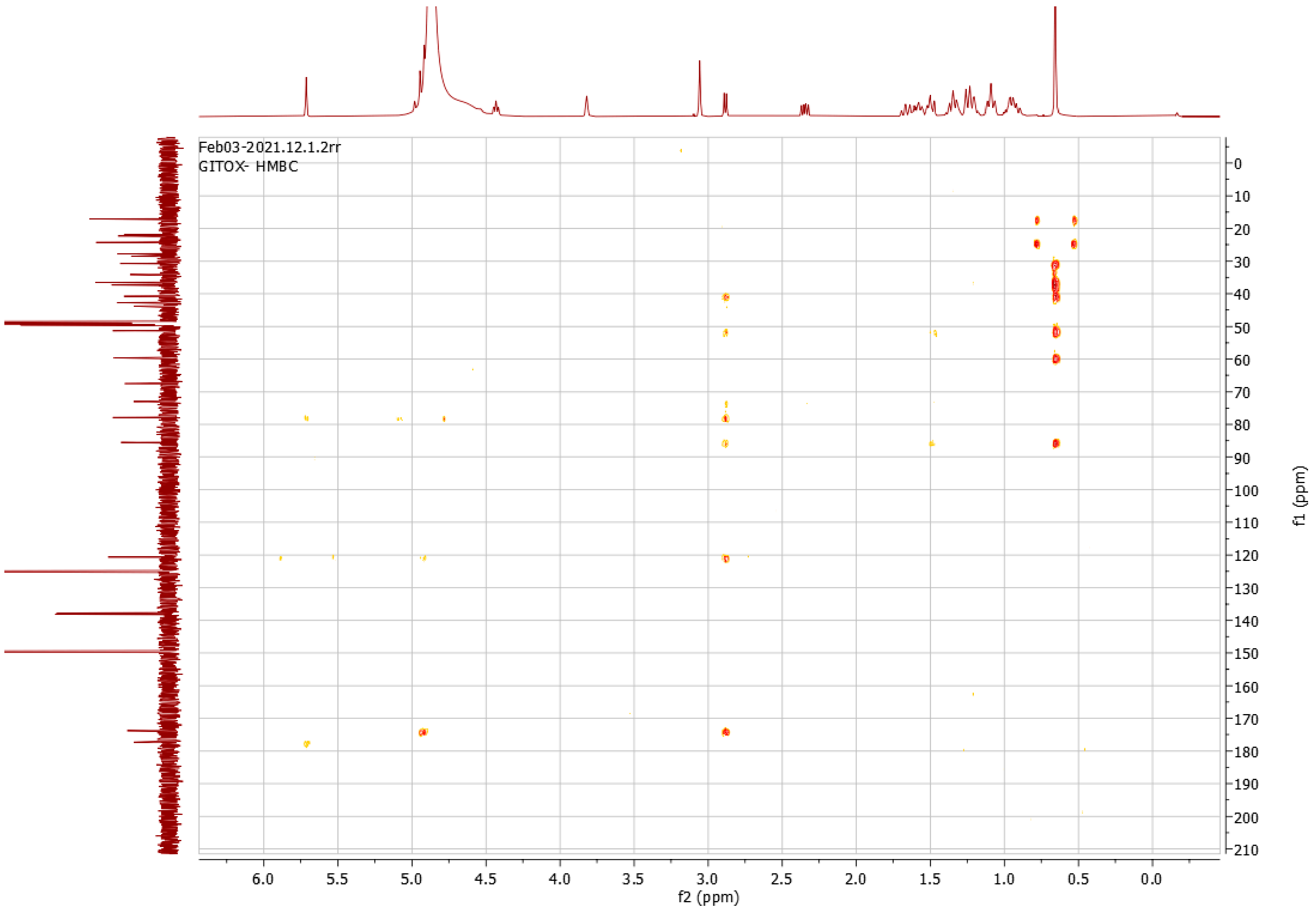

Figure S8. HMBC Spectrum of substrate gitoxigenin

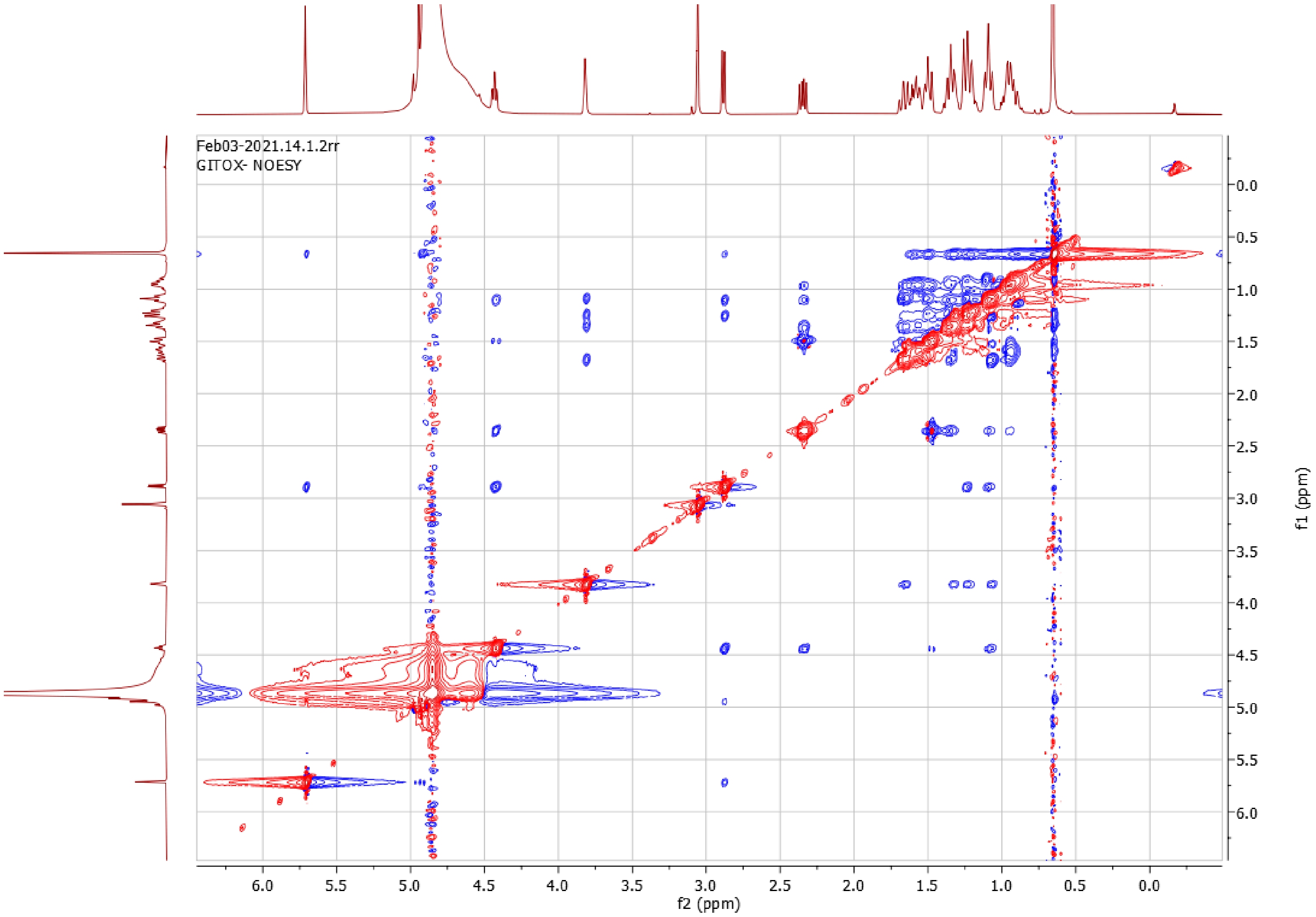

Figure S9. NOESY Spectrum of substrate gitoxigenin

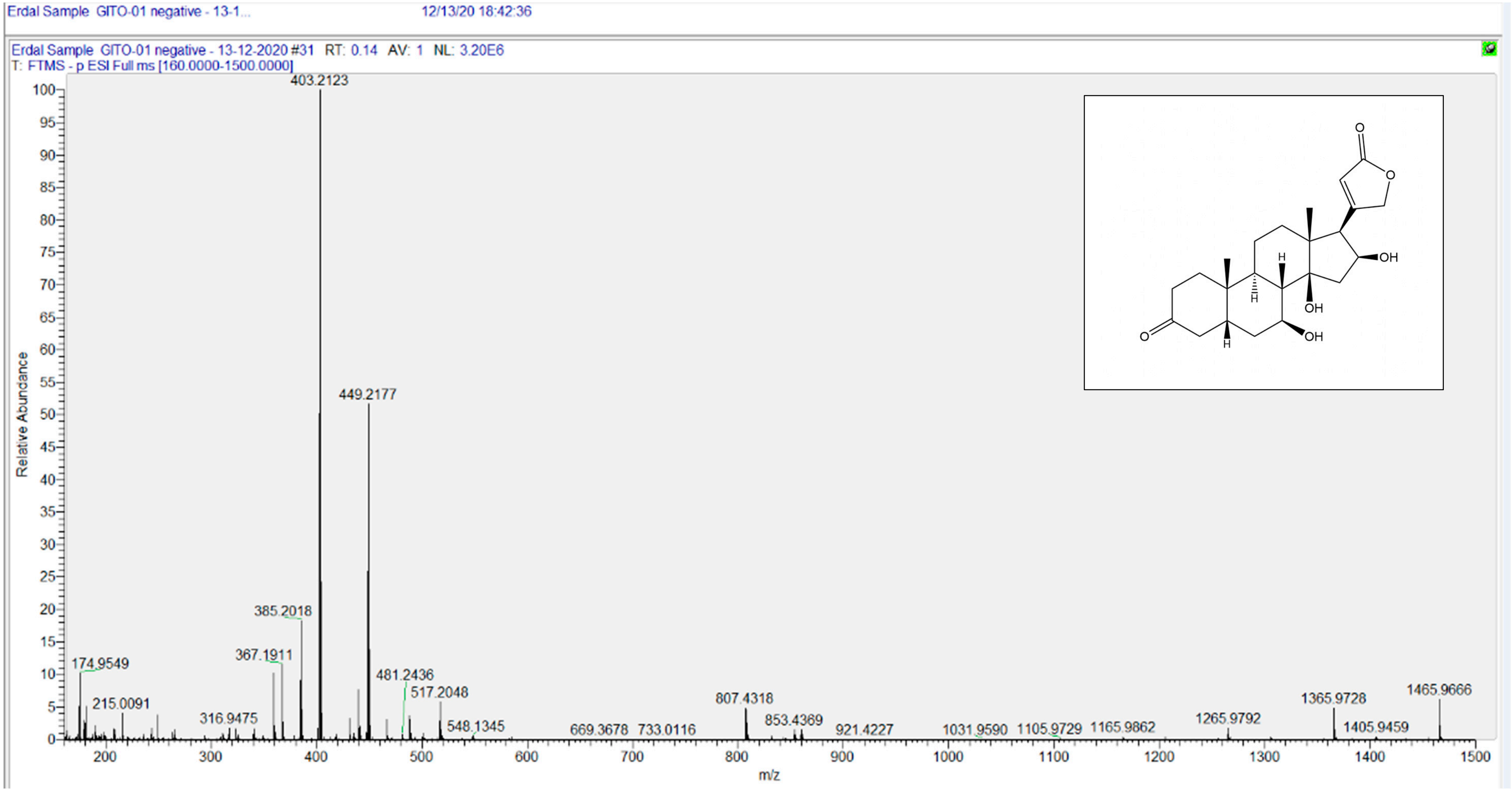

Figure S10. Negative-ion mode HR-ESI-MS spectrum of 7(β)-hydroxy-3-oxo-gitoxigenin (**compound 1**)

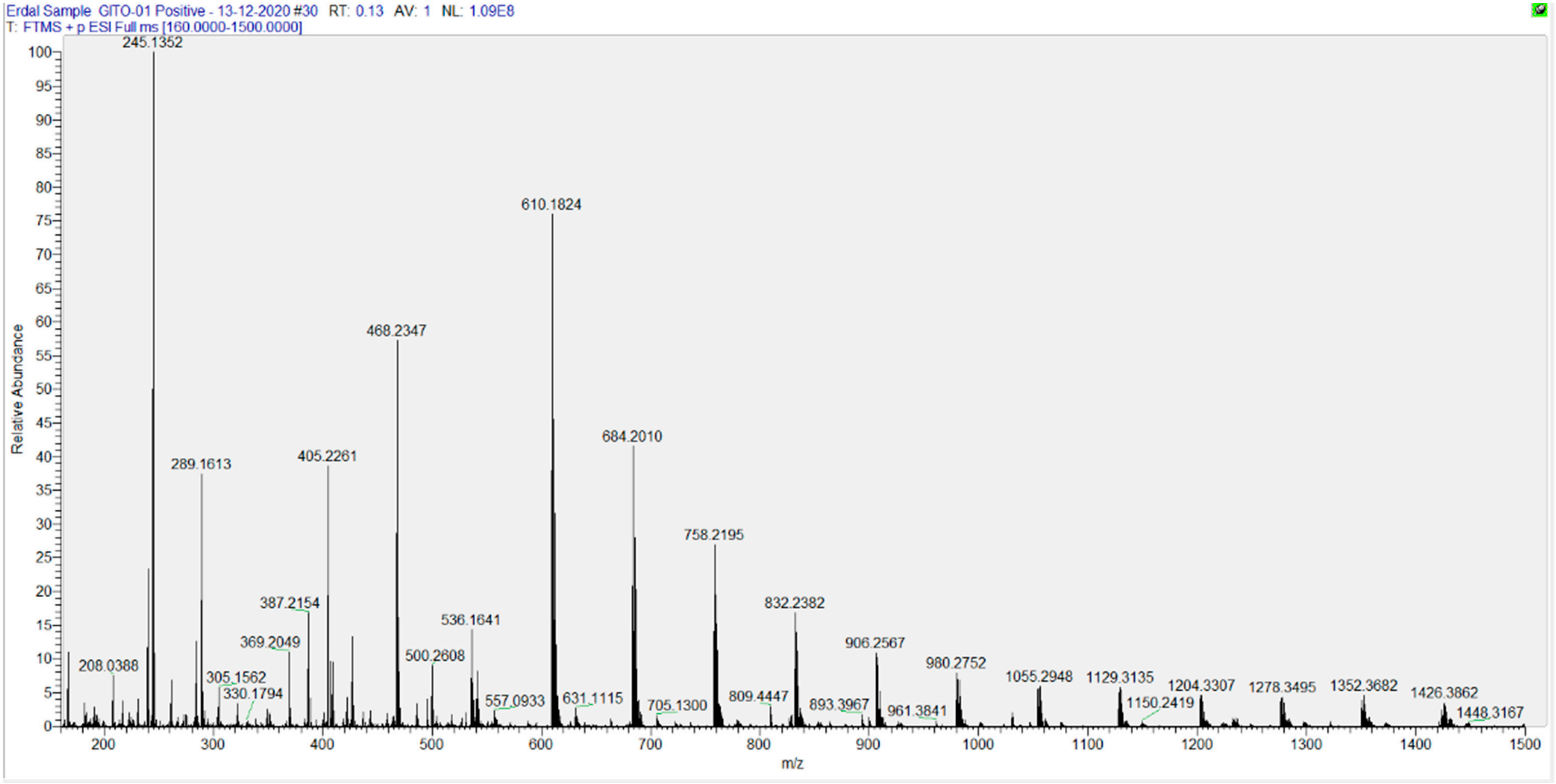

Figure S11. Positive-ion mode HR-ESI-MS spectrum of 7(β)-hydroxy-3-oxo-gitoxigenin (**compound 1**)

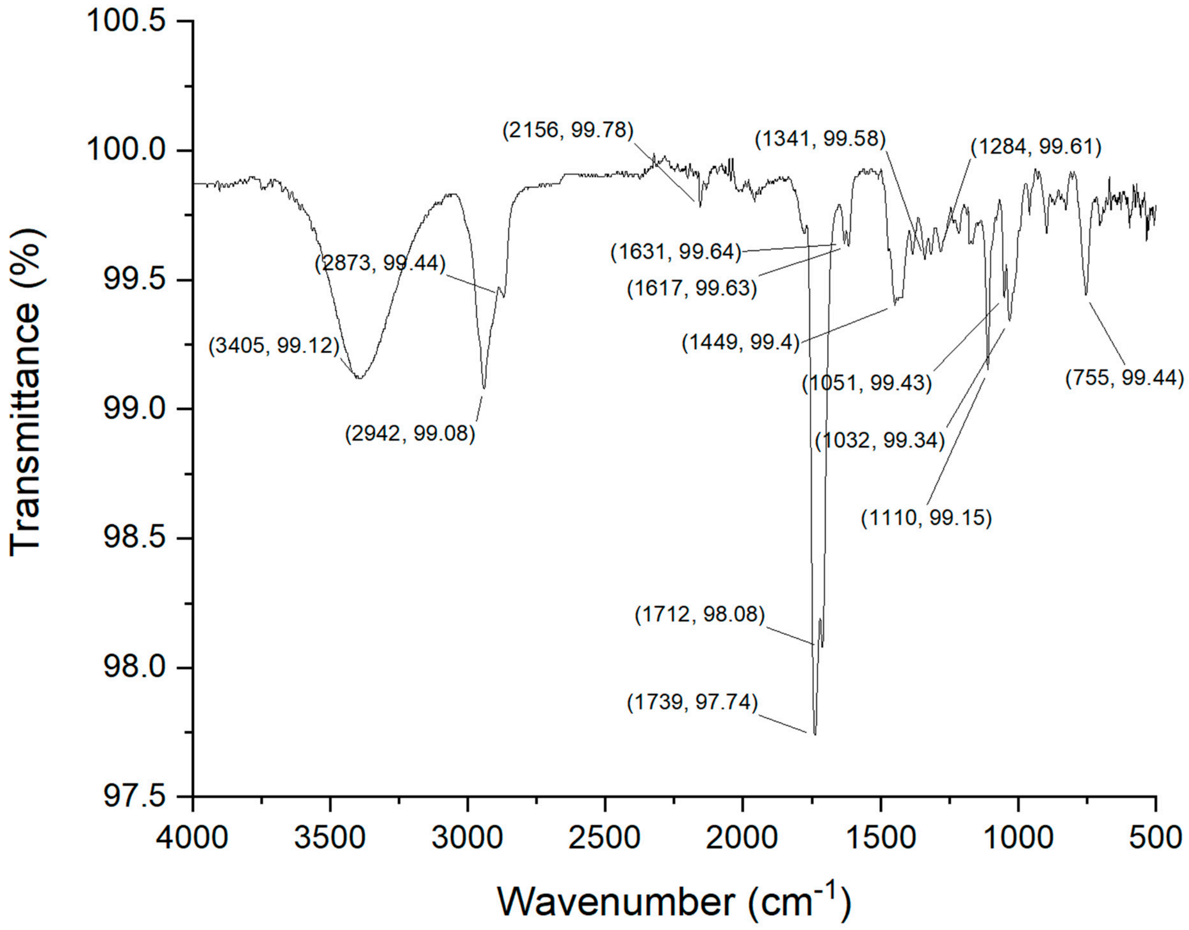

Figure S12. FT-IR Spectrum of 7(*β*)-hydroxy-3-oxo-gitoxigenin (**compound 1**)

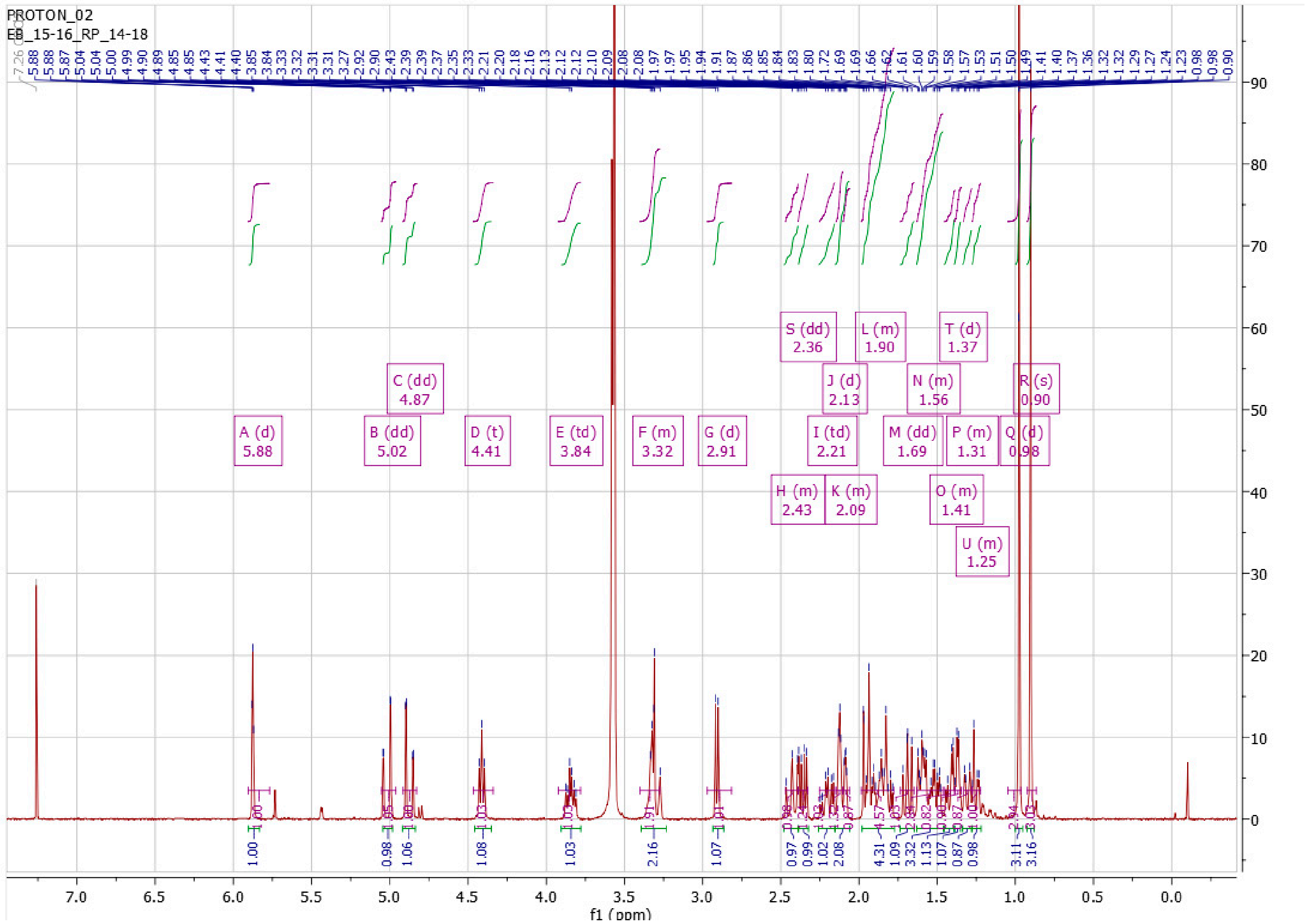

Figure S13. ^{1}H NMR Spectrum of 7(β)-hydroxy-3-oxo-gitoxigenin (**compound 1**)

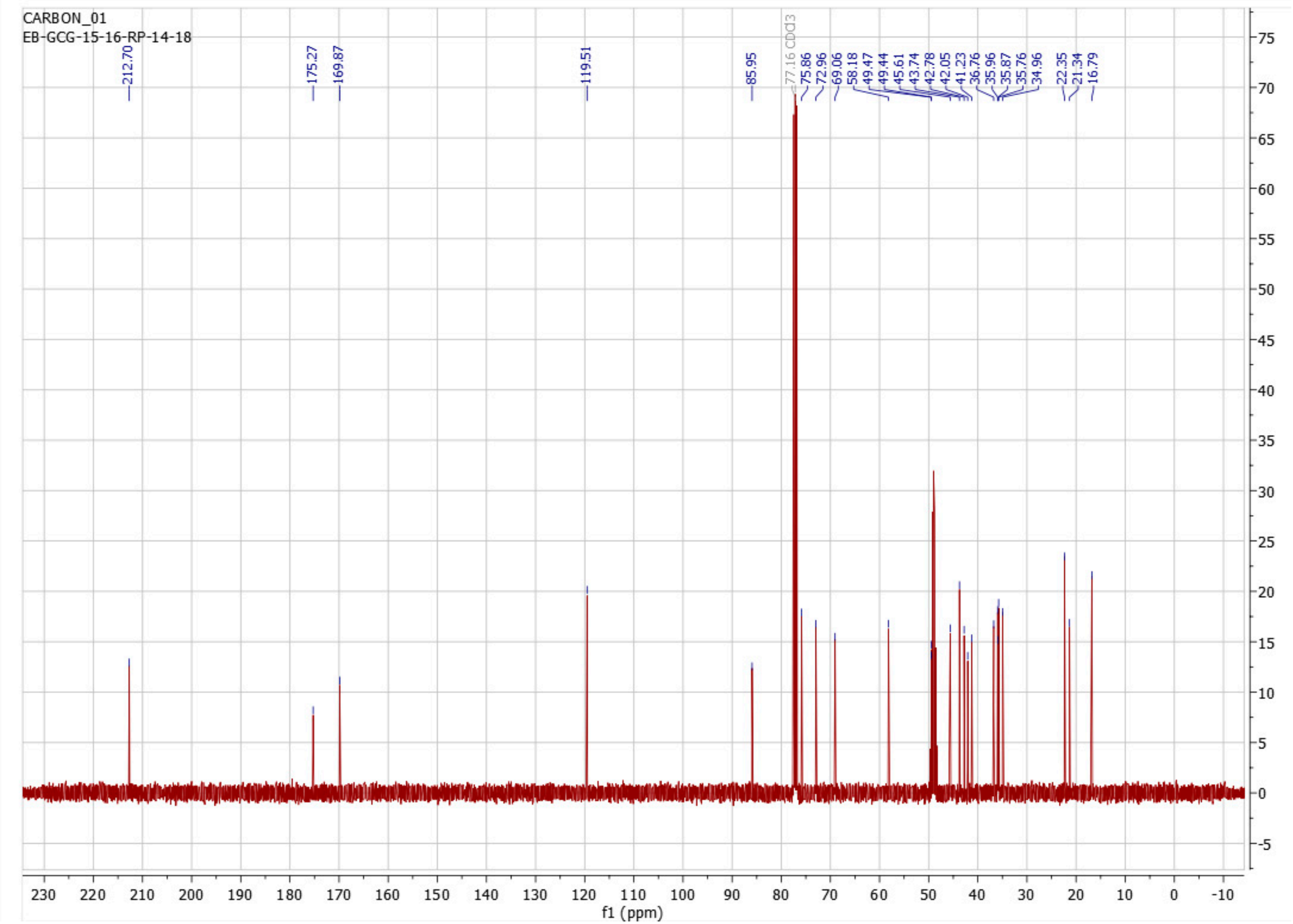

Figure S14. ^{13}C NMR Spectrum of 7(β)-hydroxy-3-oxo-gitoxigenin (**compound 1**)

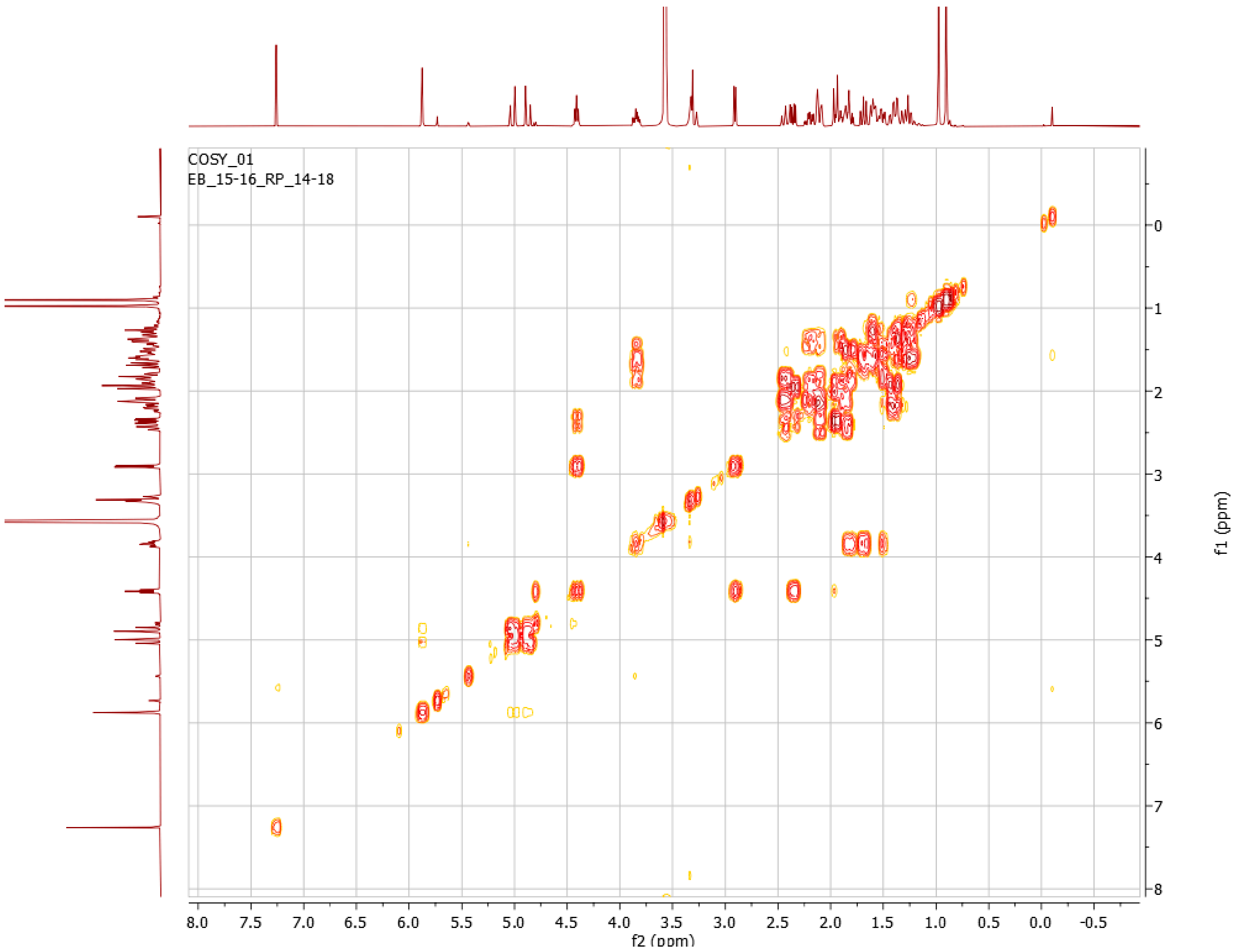

Figure S15. COSY Spectrum of 7(*β*)-hydroxy-3-oxo-gitoxigenin (**compound 1**)

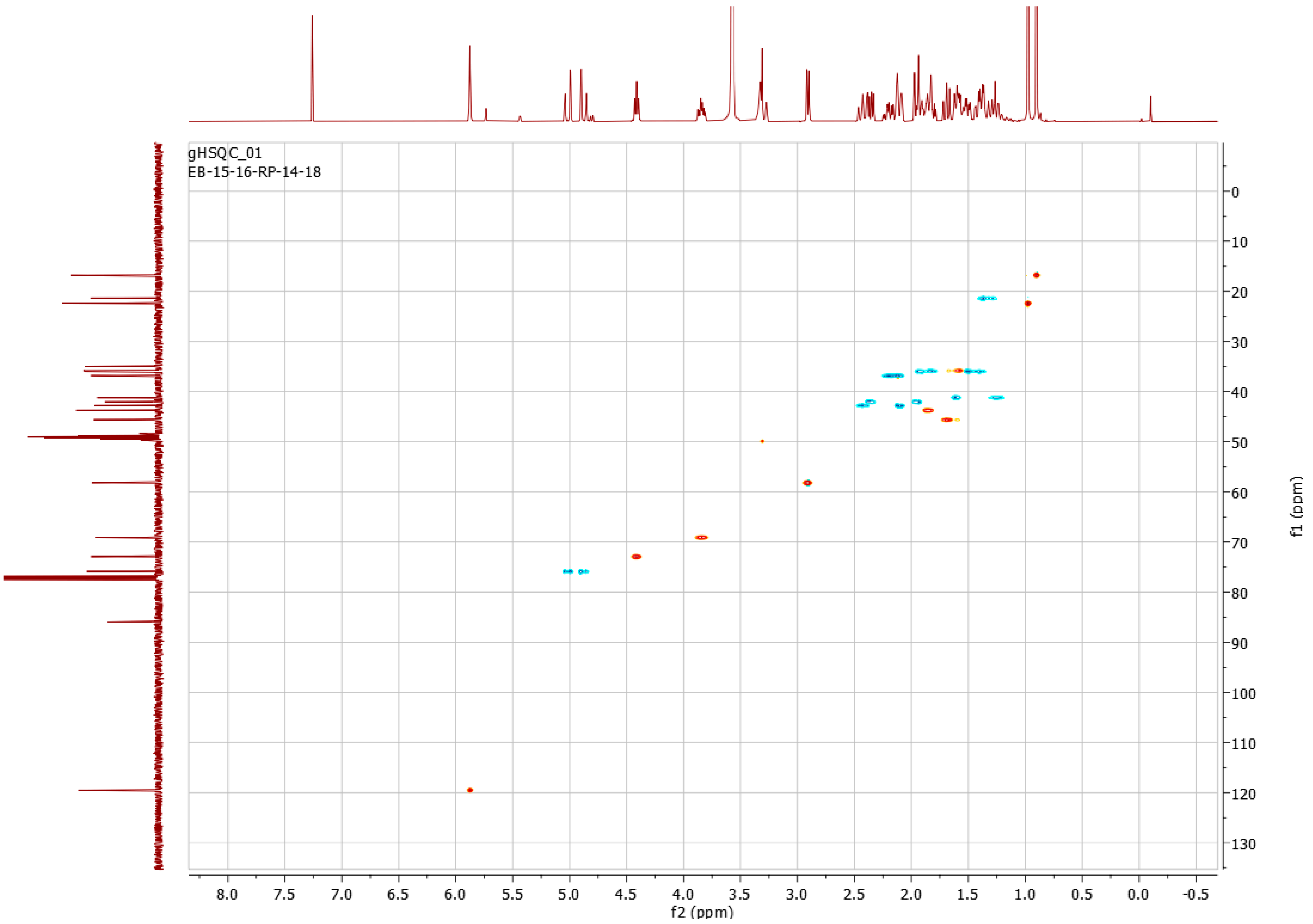

Figure S16. HSQC Spectrum of 7(β)-hydroxy-3-oxo-gitoxigenin (**compound 1**)

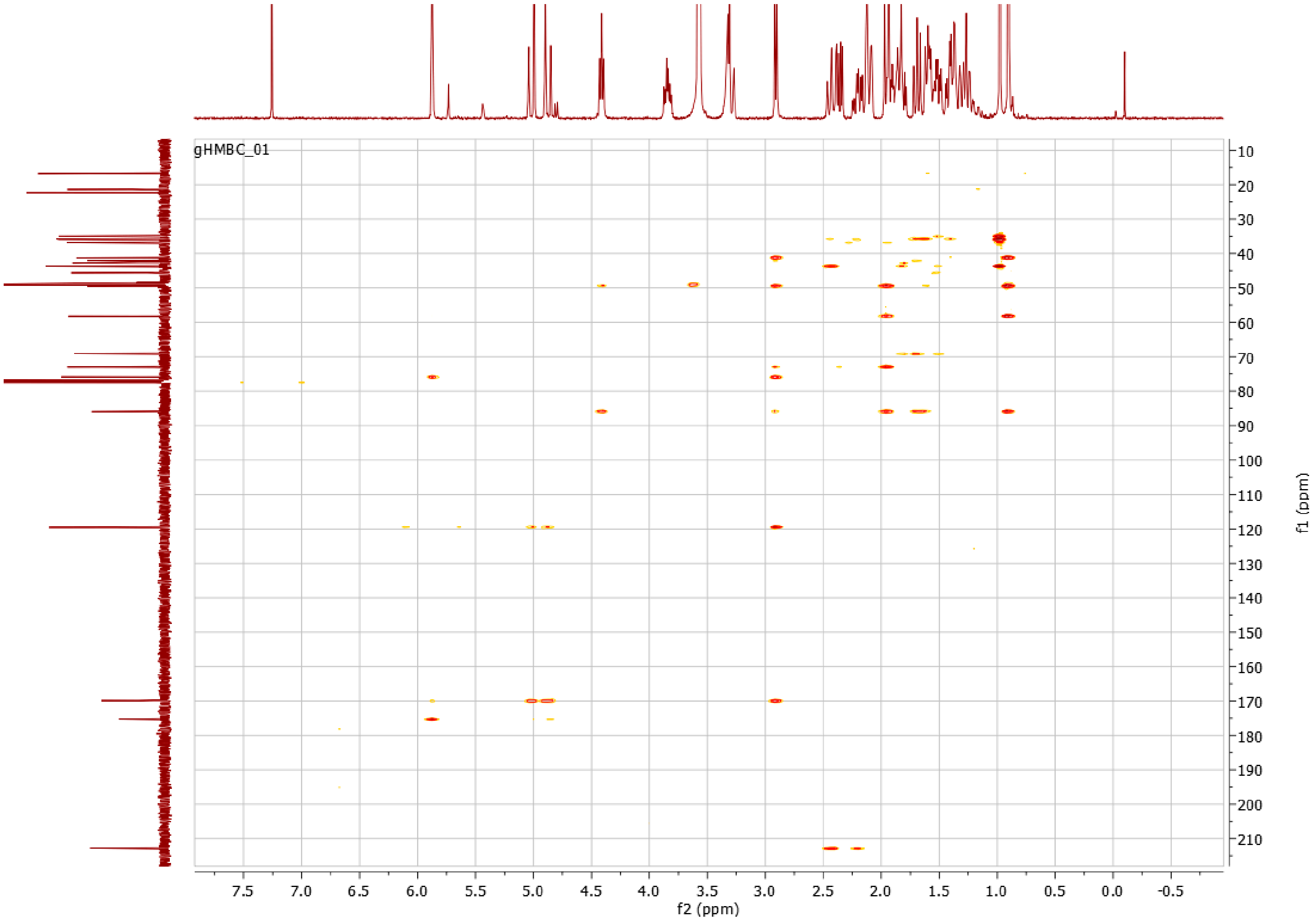

Figure S17. HMBC Spectrum of 7(β)-hydroxy-3-oxo-gitoxigenin (**compound 1**)

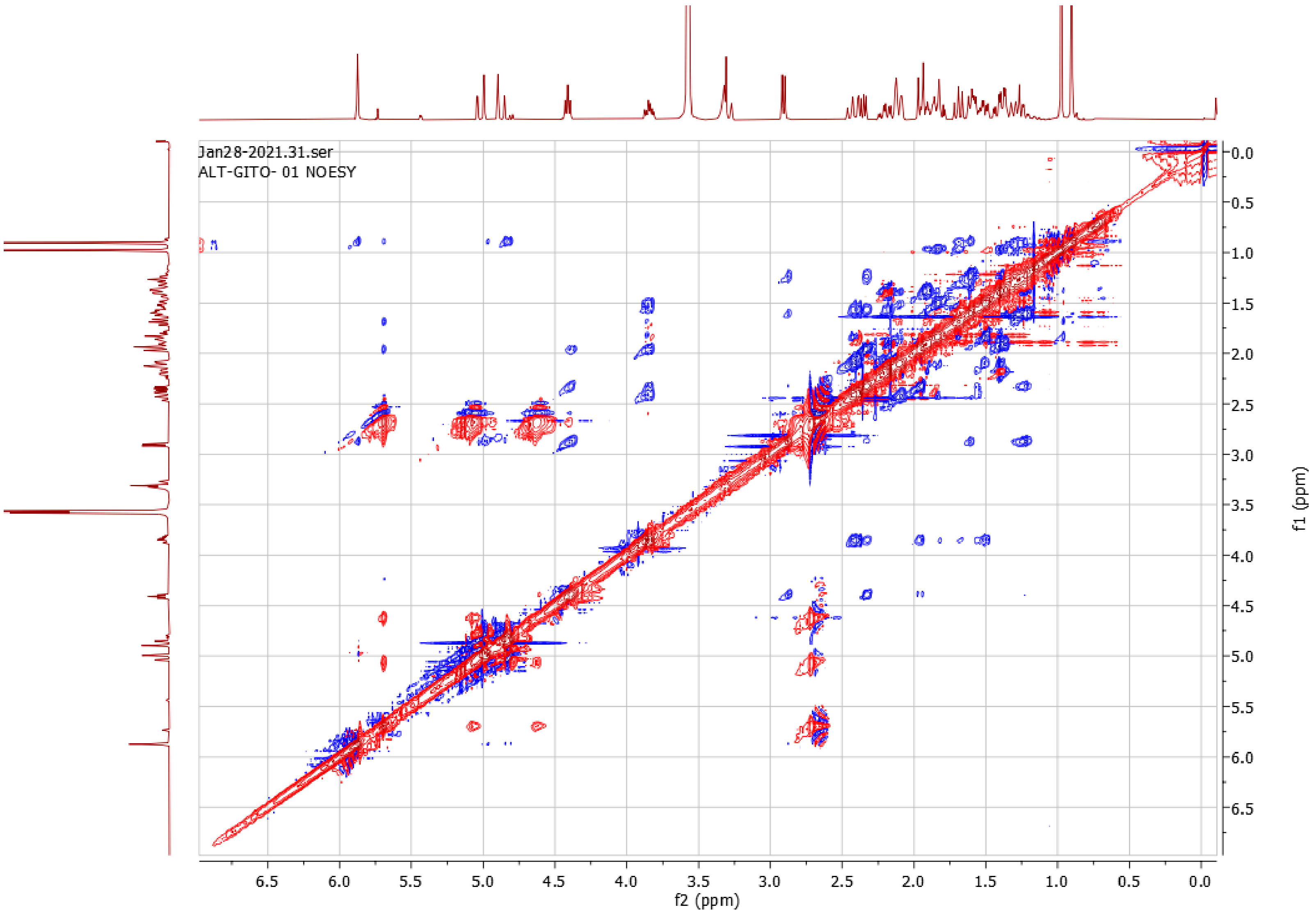

Figure S18. NOESY Spectrum of 7(β)-hydroxy-3-oxo-gitoxigenin (**compound 1**)

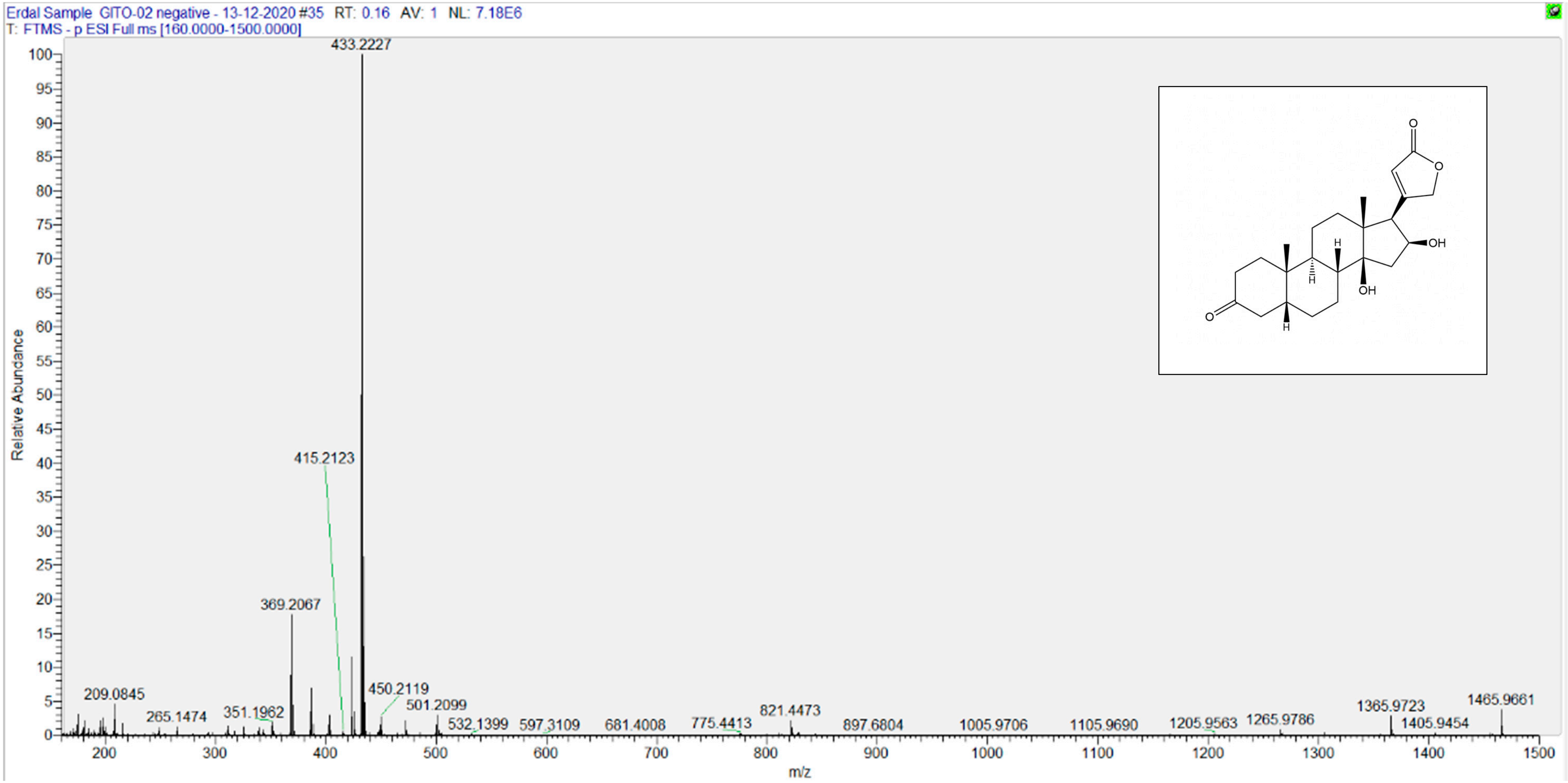

Figure S19. Negative-ion mode HR-ESI-MS spectrum of 3-oxo-gitoxigenin (**compound 2**)

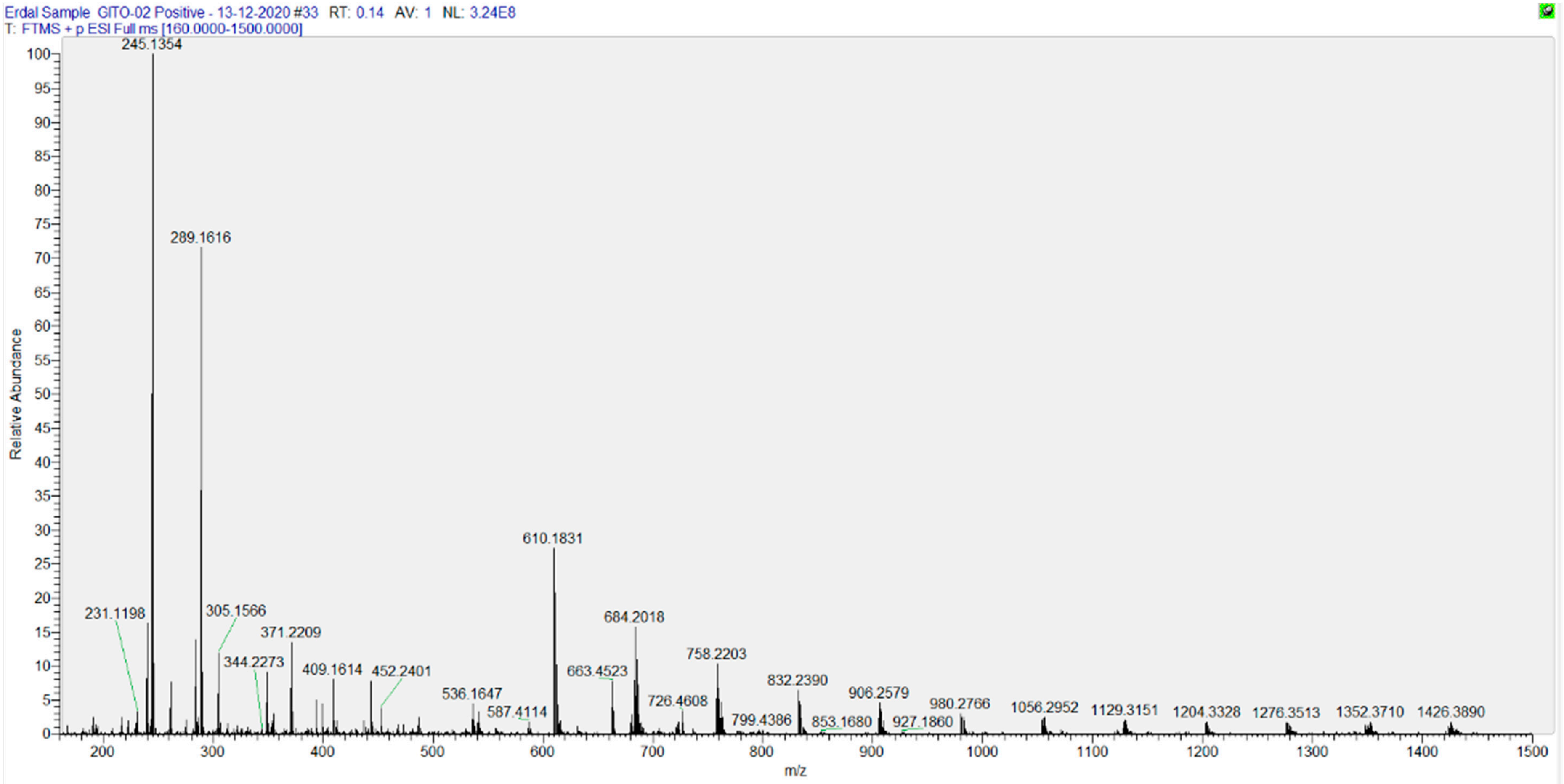

Figure S20. Positive-ion mode HR-ESI-MS spectrum of 3-oxo-gitoxigenin (**compound 2**)

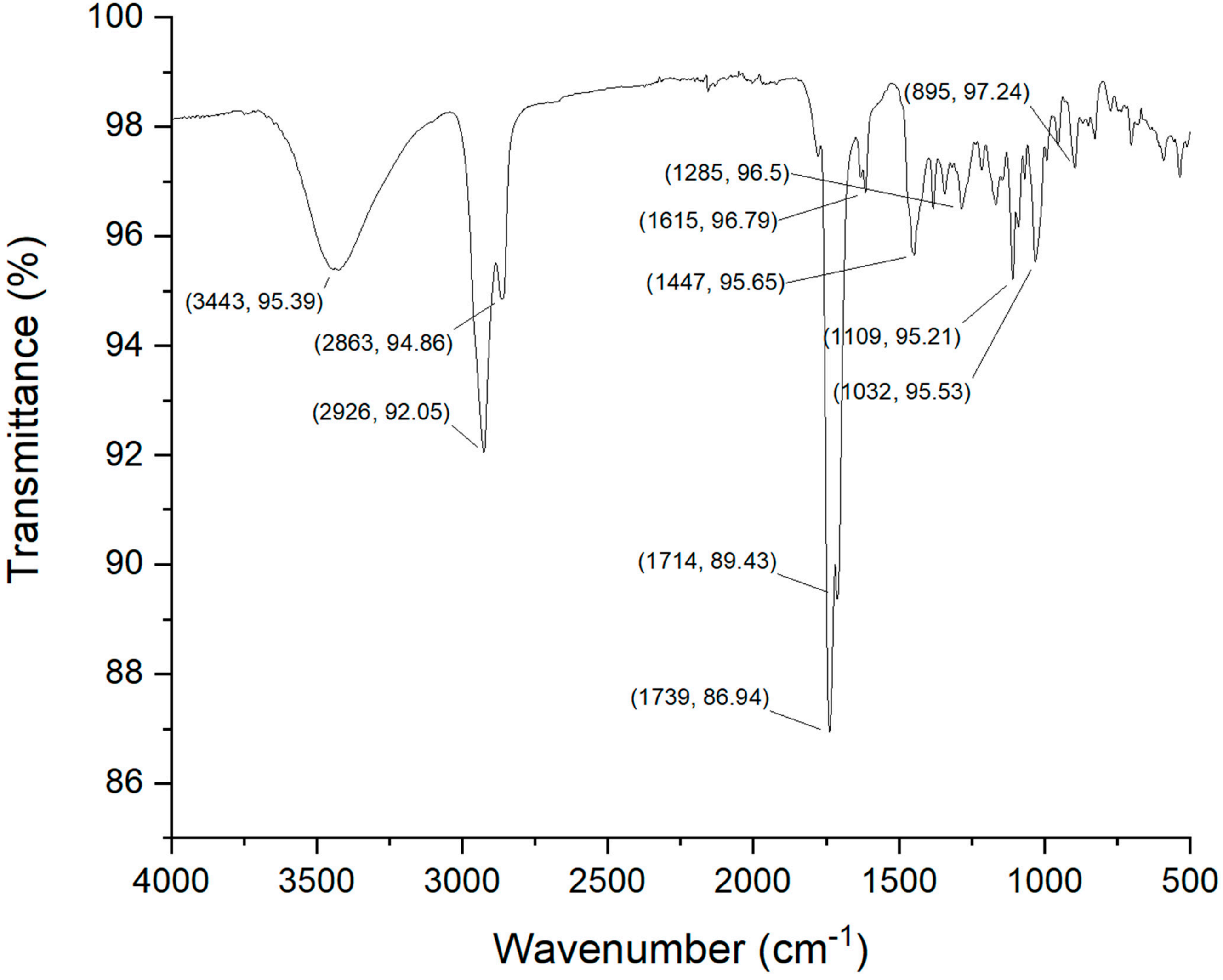

Figure S21. FT-IR Spectrum of 3-oxo-gitoxigenin (**compound 2**)

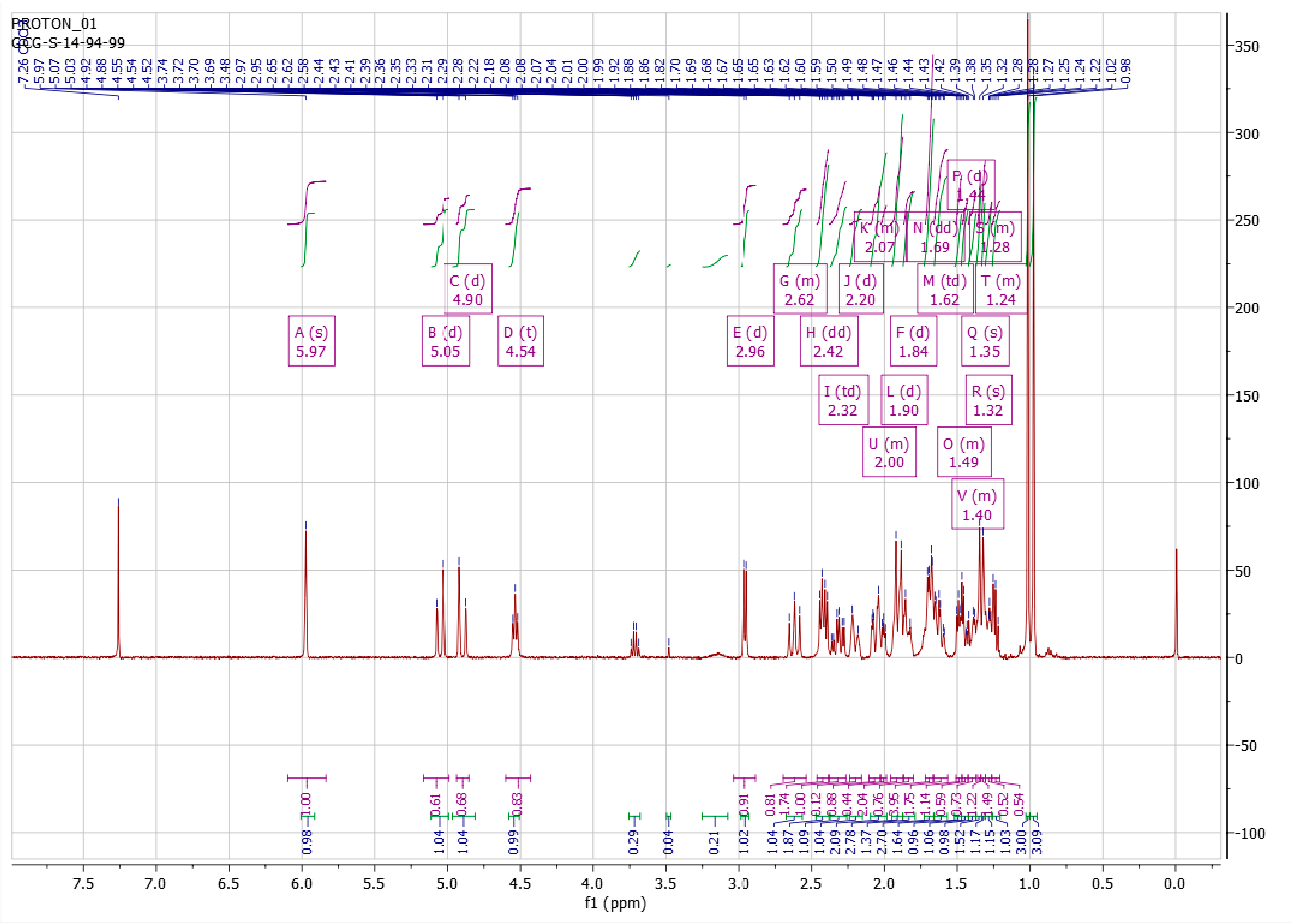

Figure S22. ^{1}H NMR Spectrum of 3-oxo-gitoxigenin (**compound 2**)

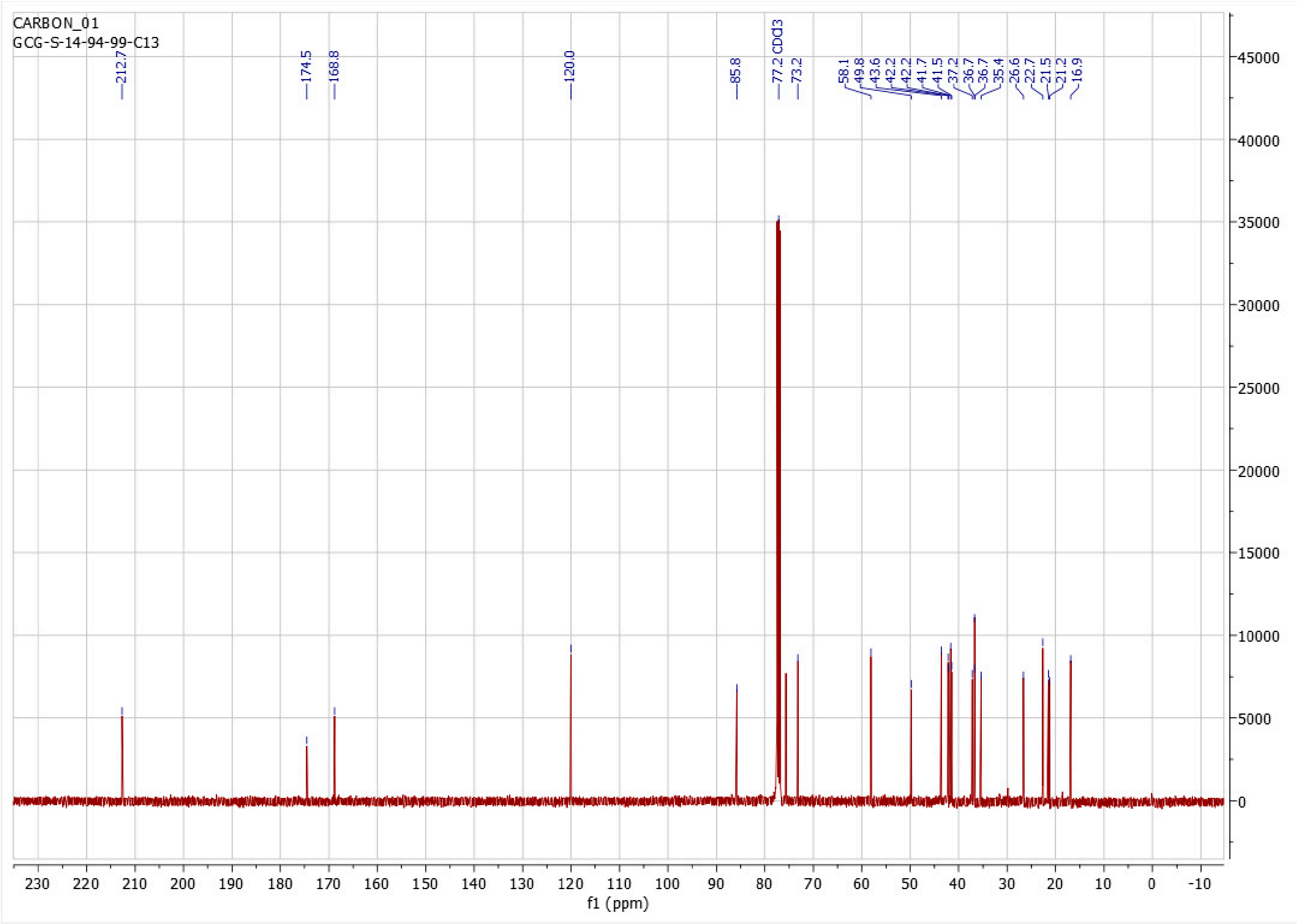

Figure S23. ^{13}C NMR Spectrum of 3-oxo-gitoxigenin (**compound 2**)

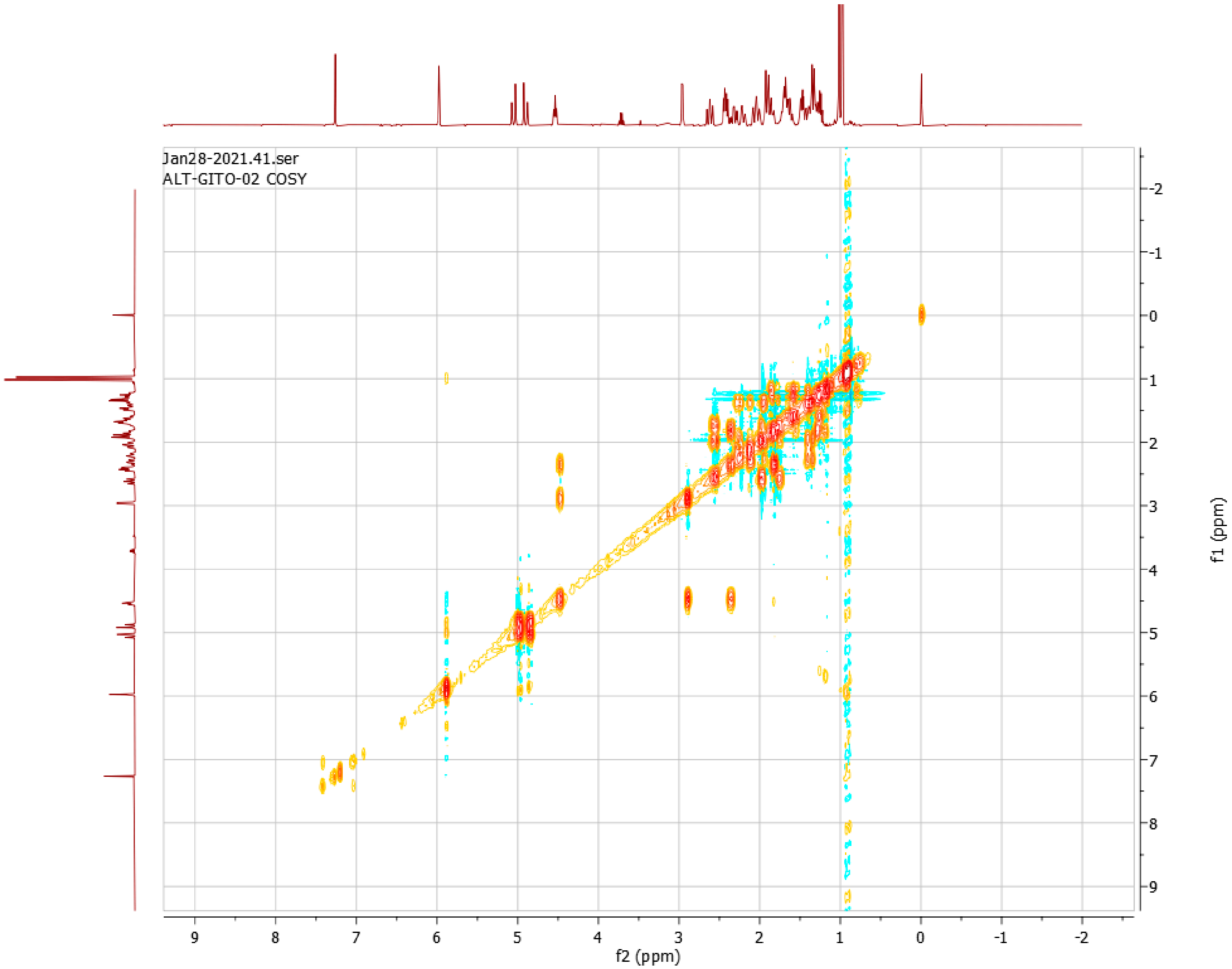

Figure S24. COSY Spectrum of 3-oxo-gitoxigenin (**compound 2**)

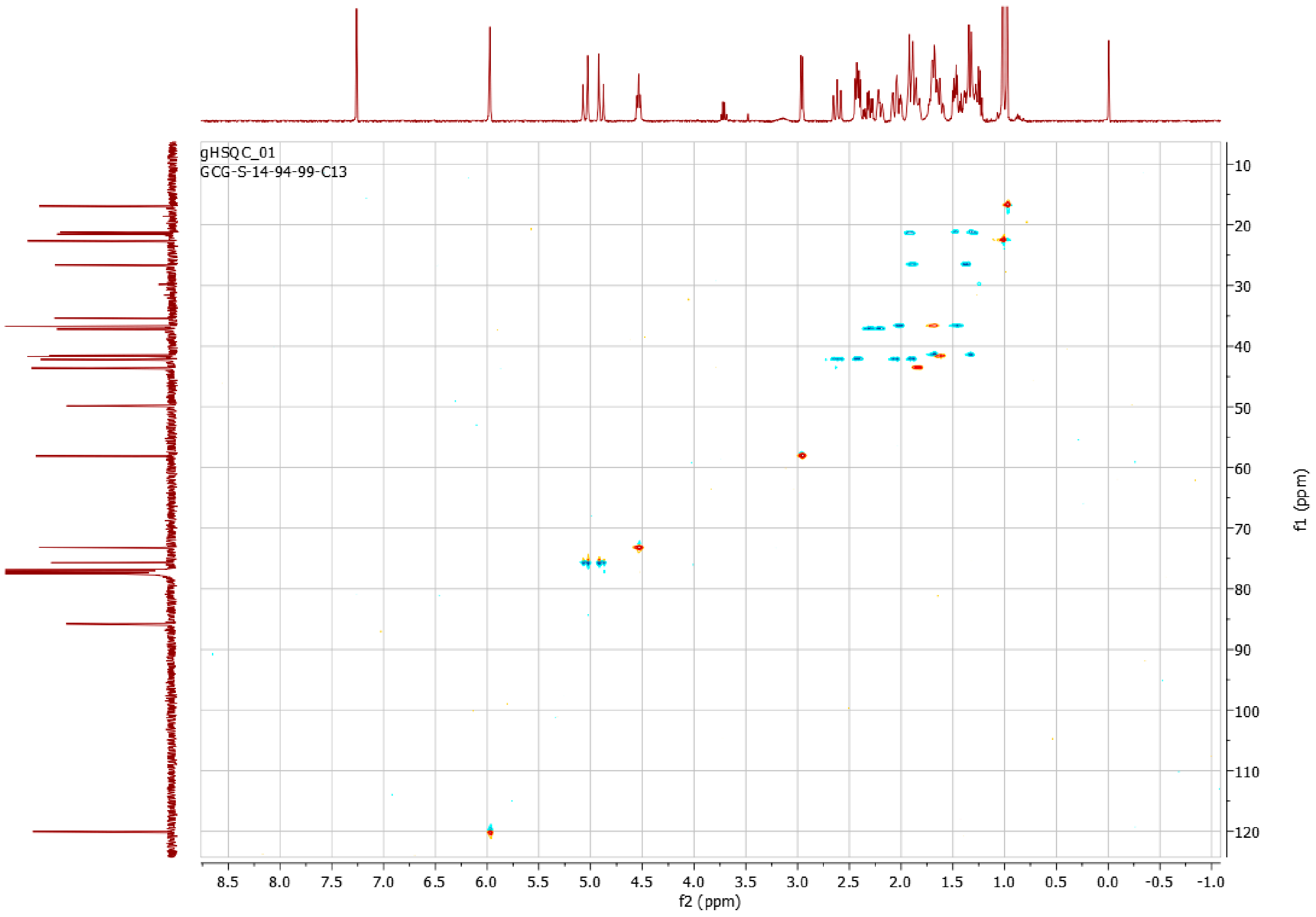

Figure S25. HSQC Spectrum of 3-oxo-gitoxigenin (**compound 2**)

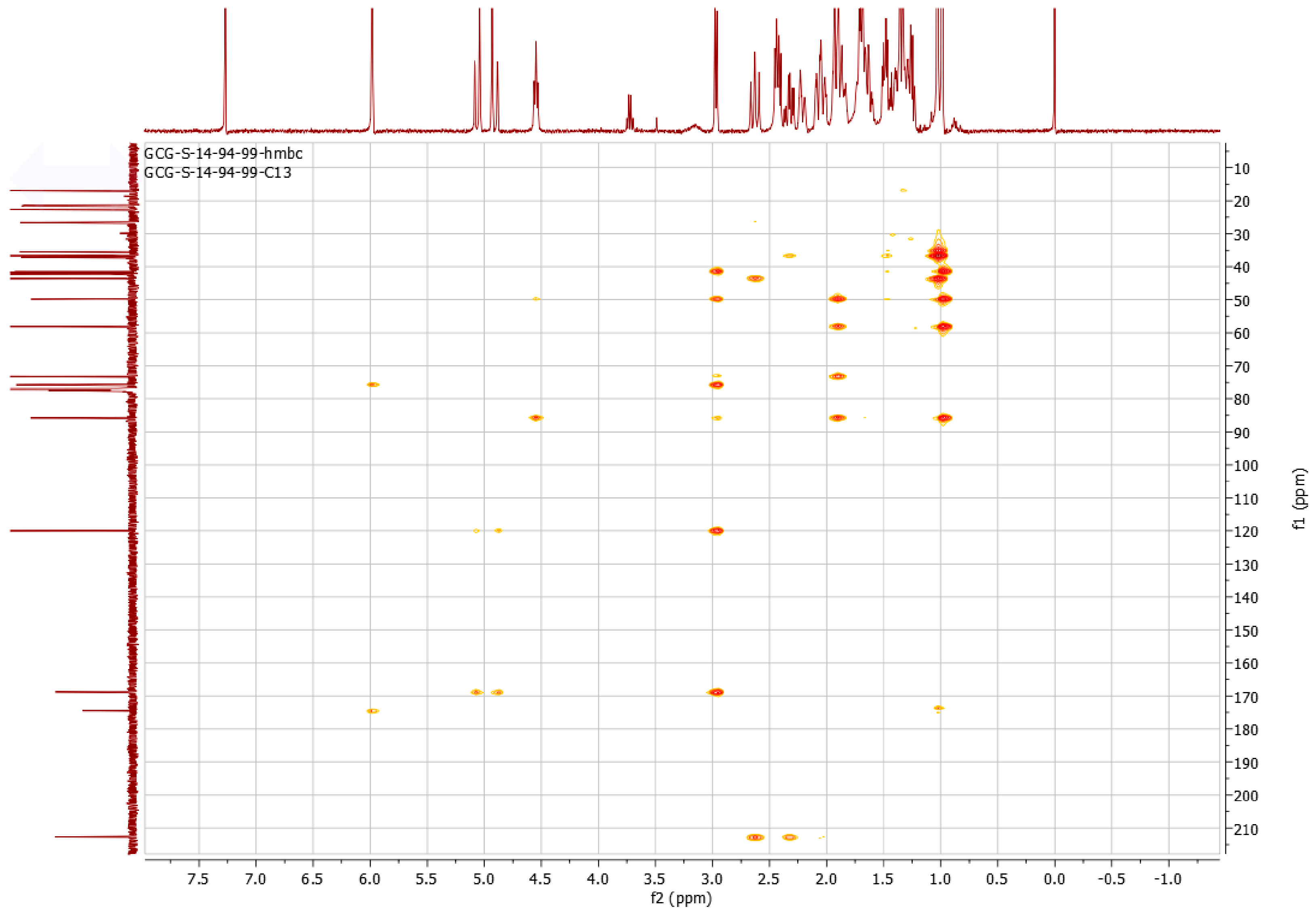

Figure S26. HMBC Spectrum of 3-oxo-gitoxigenin (**compound 2**)

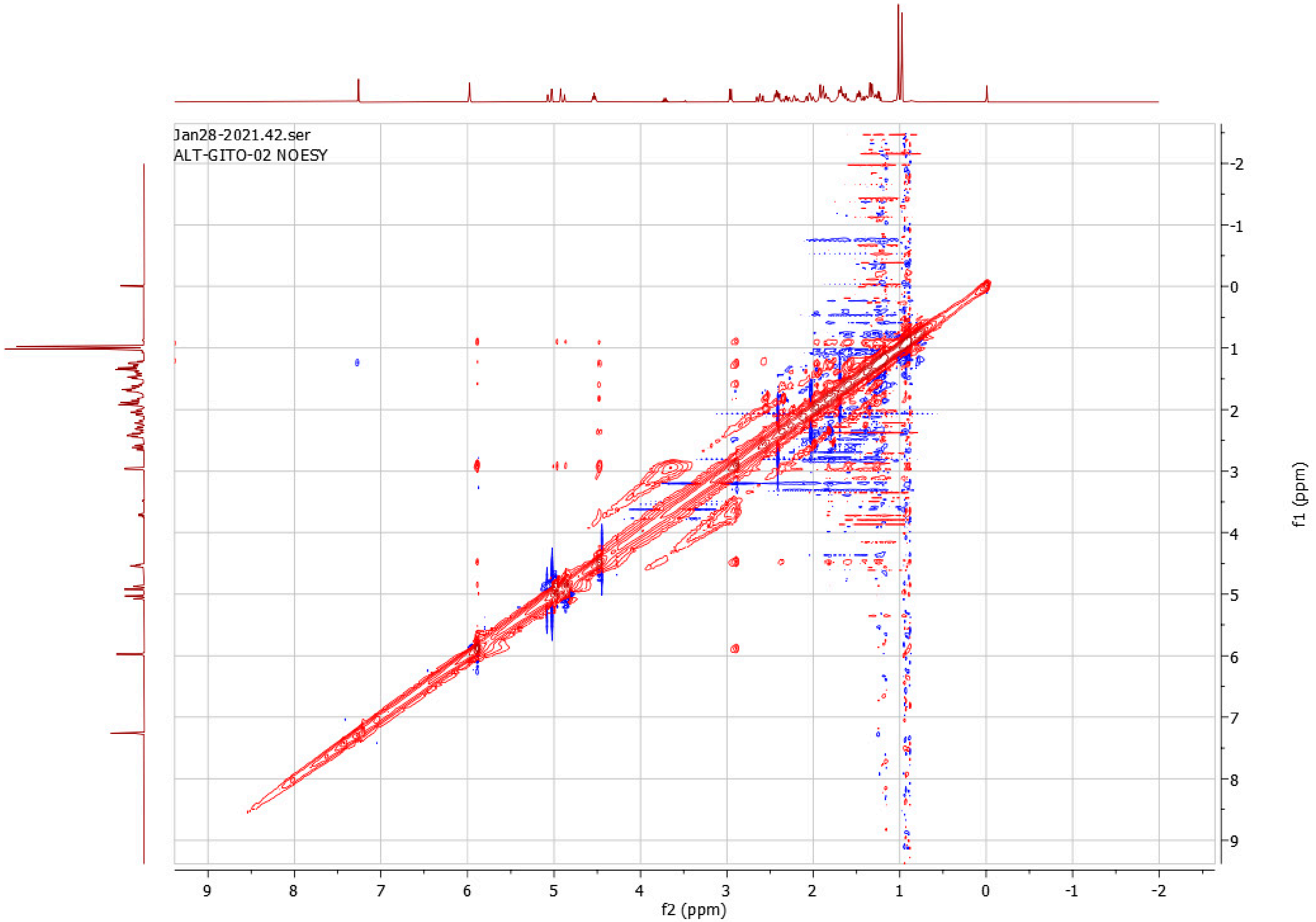

Figure S27. NOESY Spectrum of 3-oxo-gitoxigenin (**compound 2**)

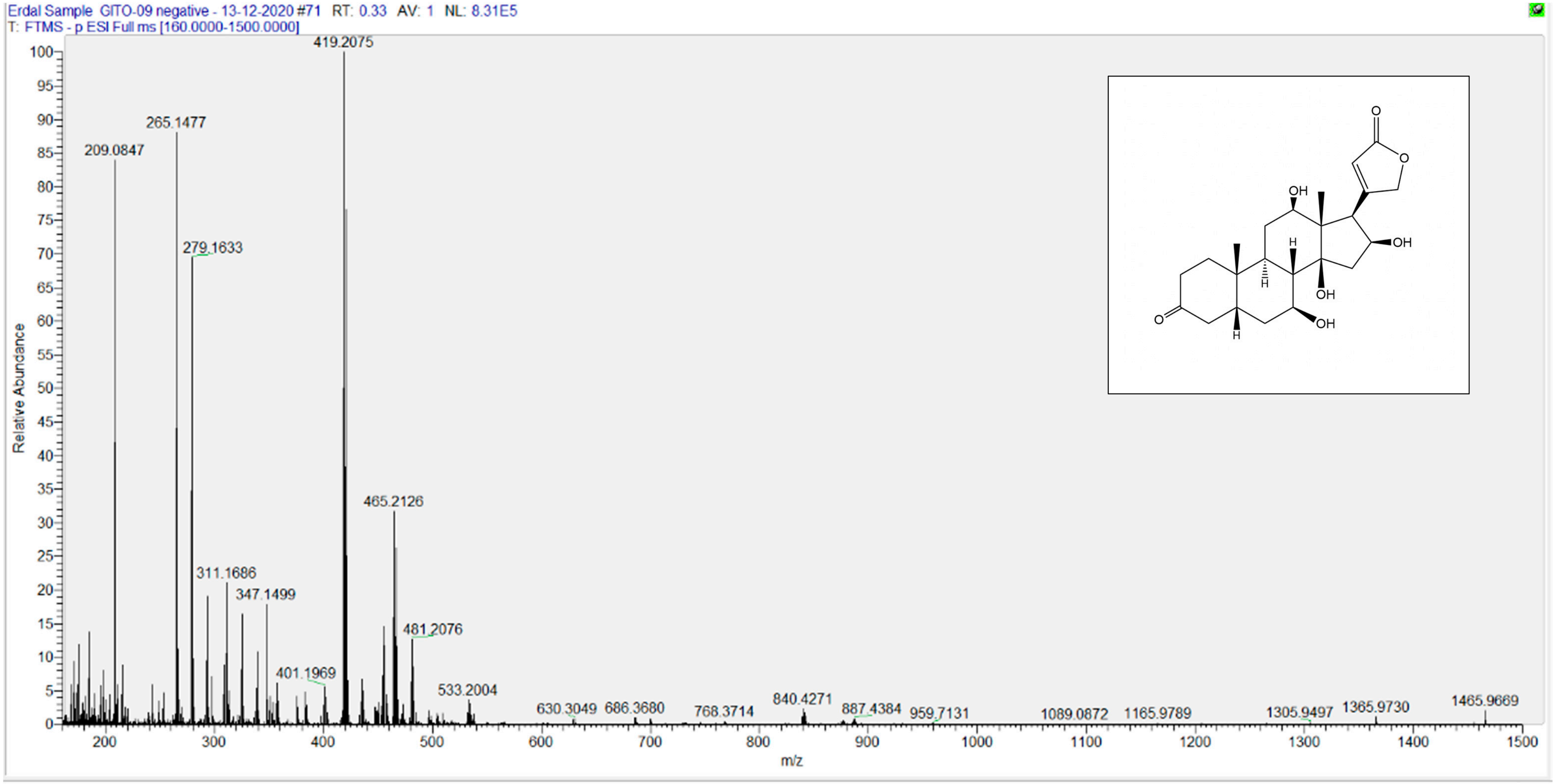

Figure S28. Negative-ion mode HR-ESI-MS spectrum of 7(β),12(β)-dihdyroxy-3-oxo-gitoxigenin (**compound 3**)

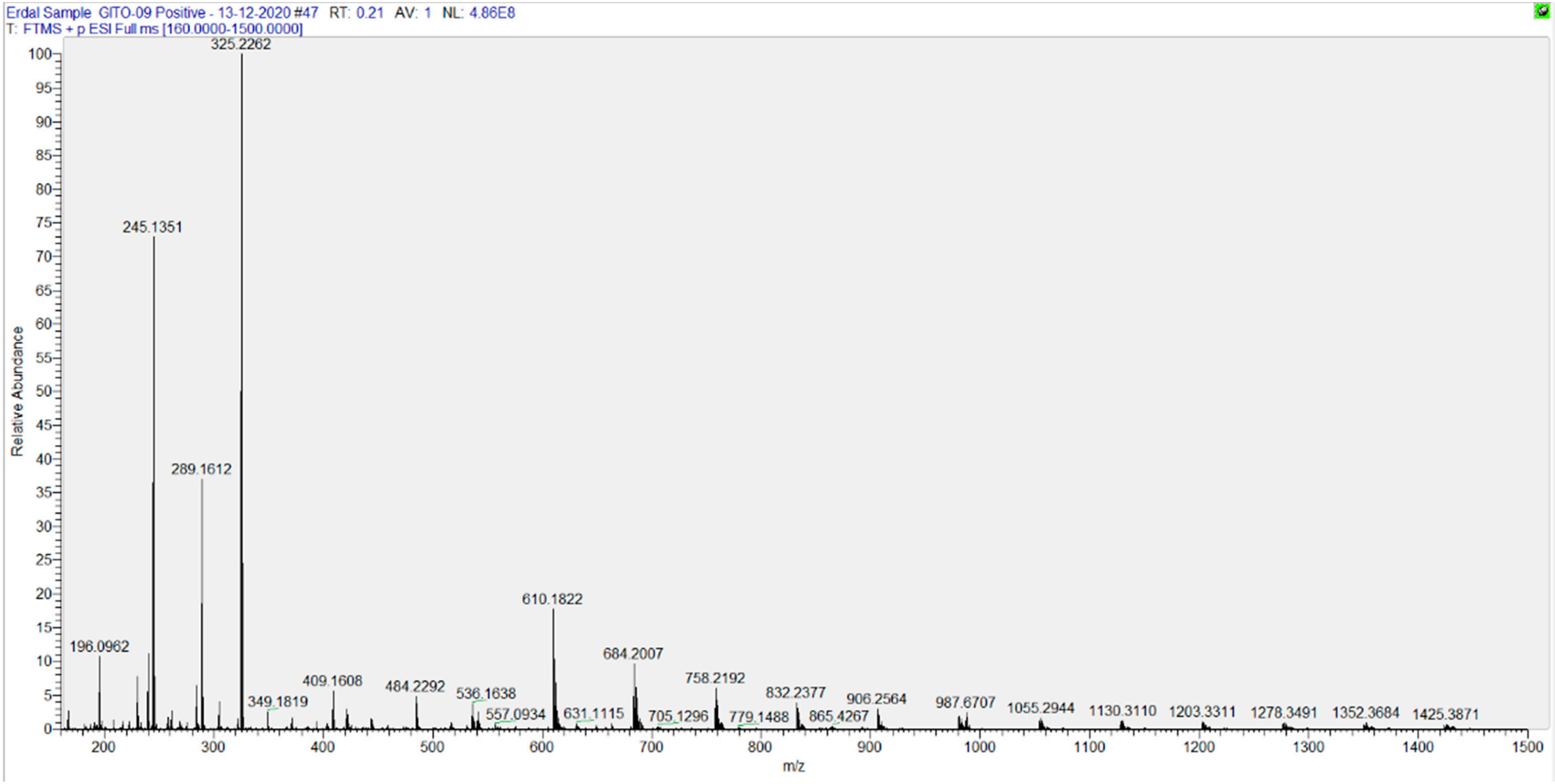

Figure S29. Positive-ion mode HR-ESI-MS spectrum of 7(*β*),12(*β*)-dihdyroxy-3-oxo-gitoxigenin (**compound 3**)

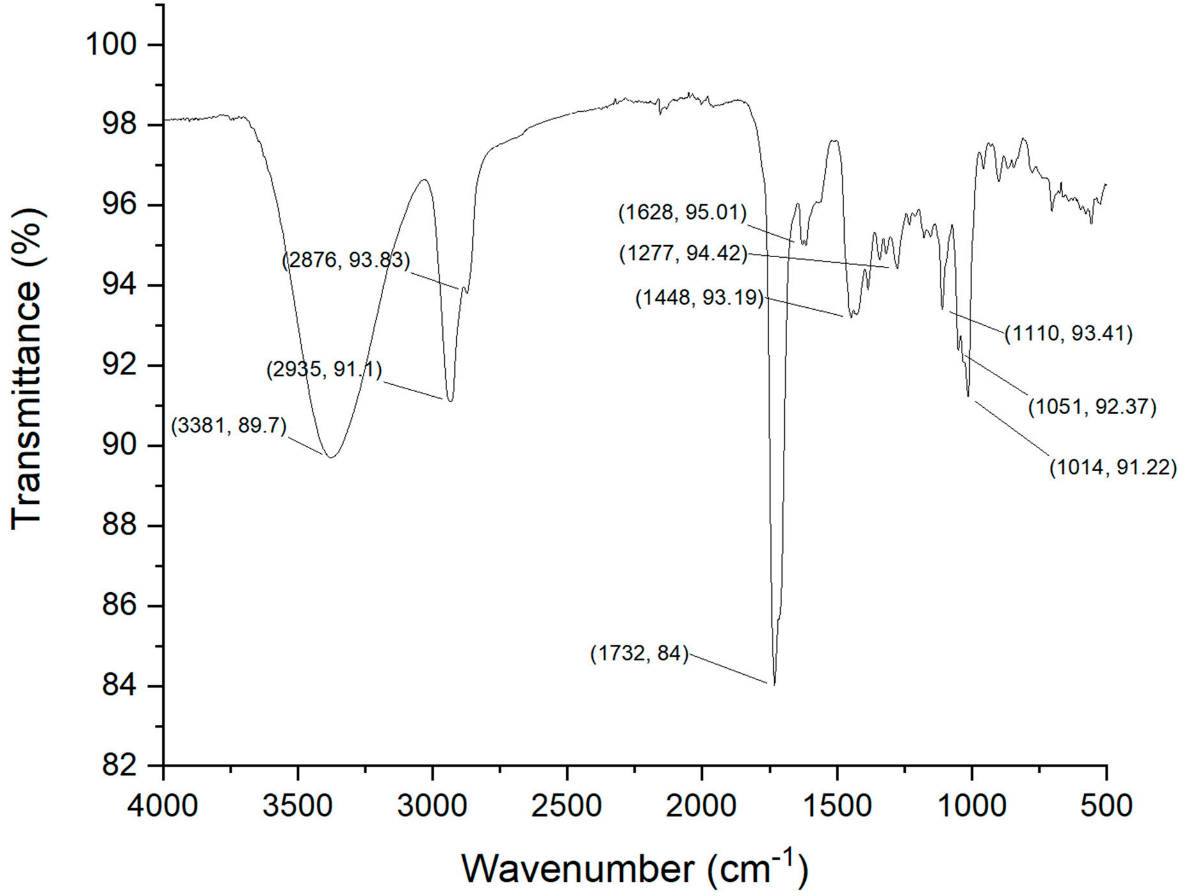

Figure S30. FT-IR Spectrum of 7(*β*),12(*β*)-dihdyroxy-3-oxo-gitoxigenin (**compound 3**)

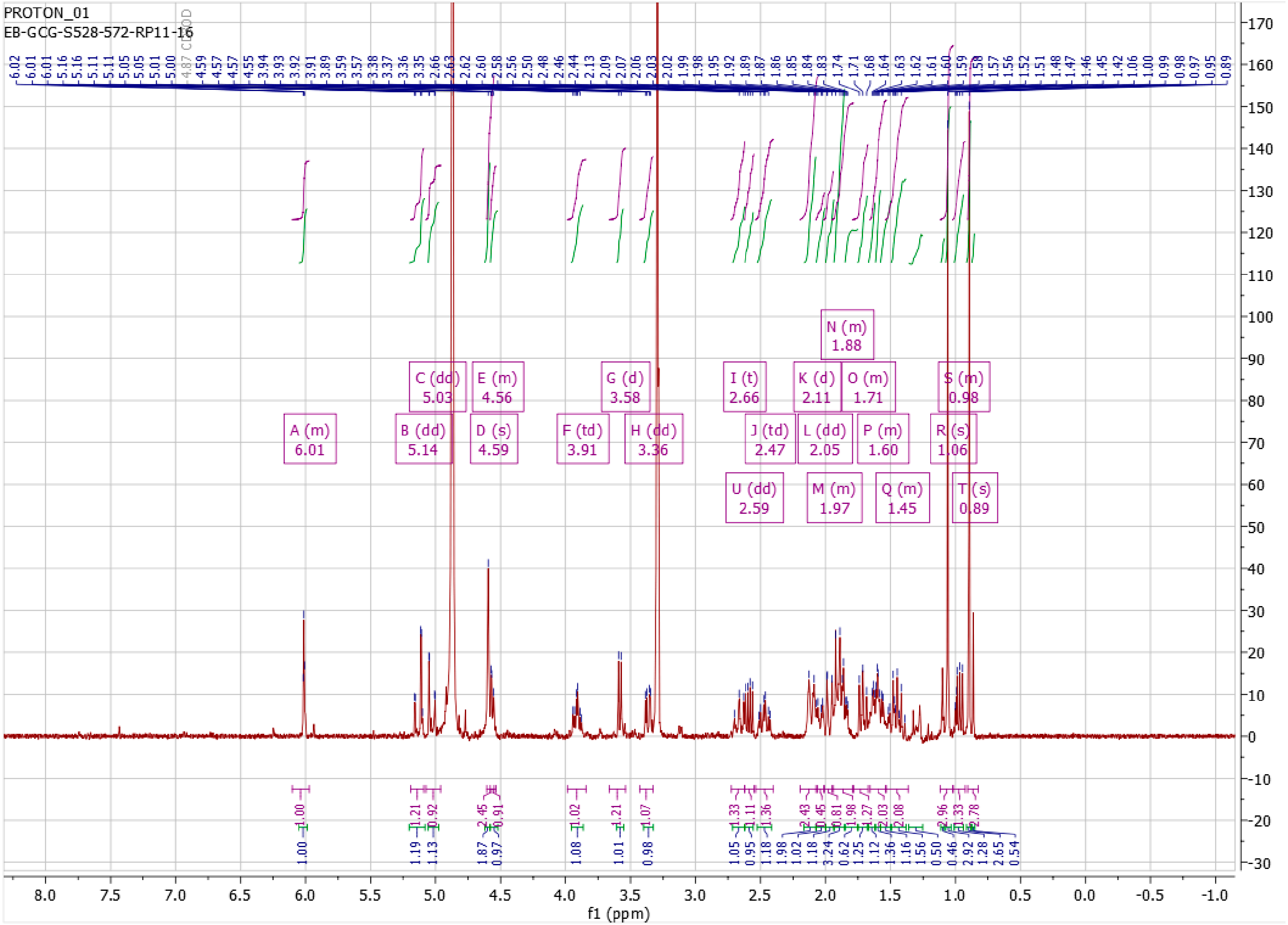

Figure S31. ^{1}H NMR Spectrum of 7(β),12(β)-dihdyroxy-3-oxo-gitoxigenin (**compound 3**)

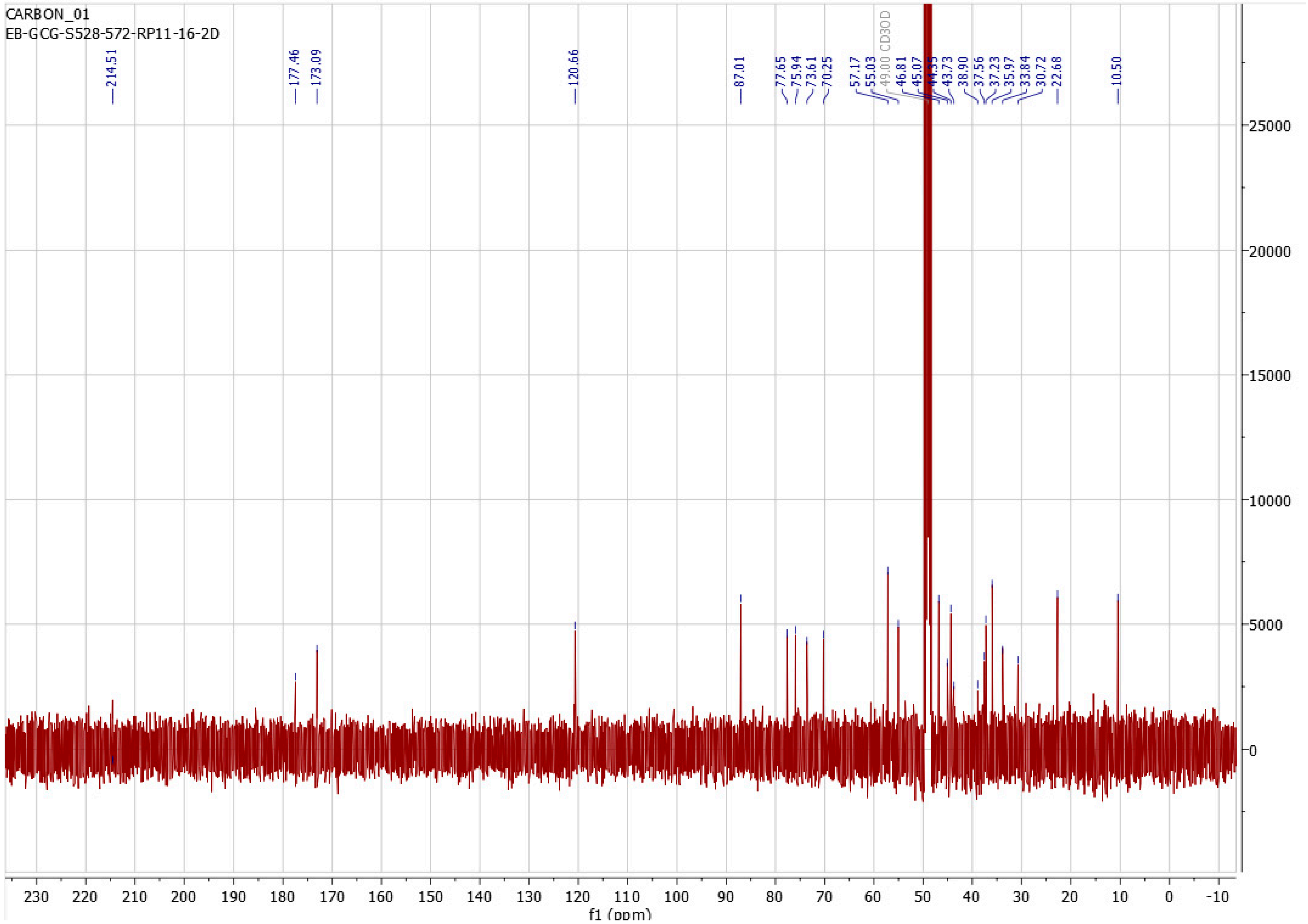

Figure S32. ^{13}C NMR Spectrum of 7(β),12(β)-dihdyroxy-3-oxo-gitoxigenin (**compound 3**)

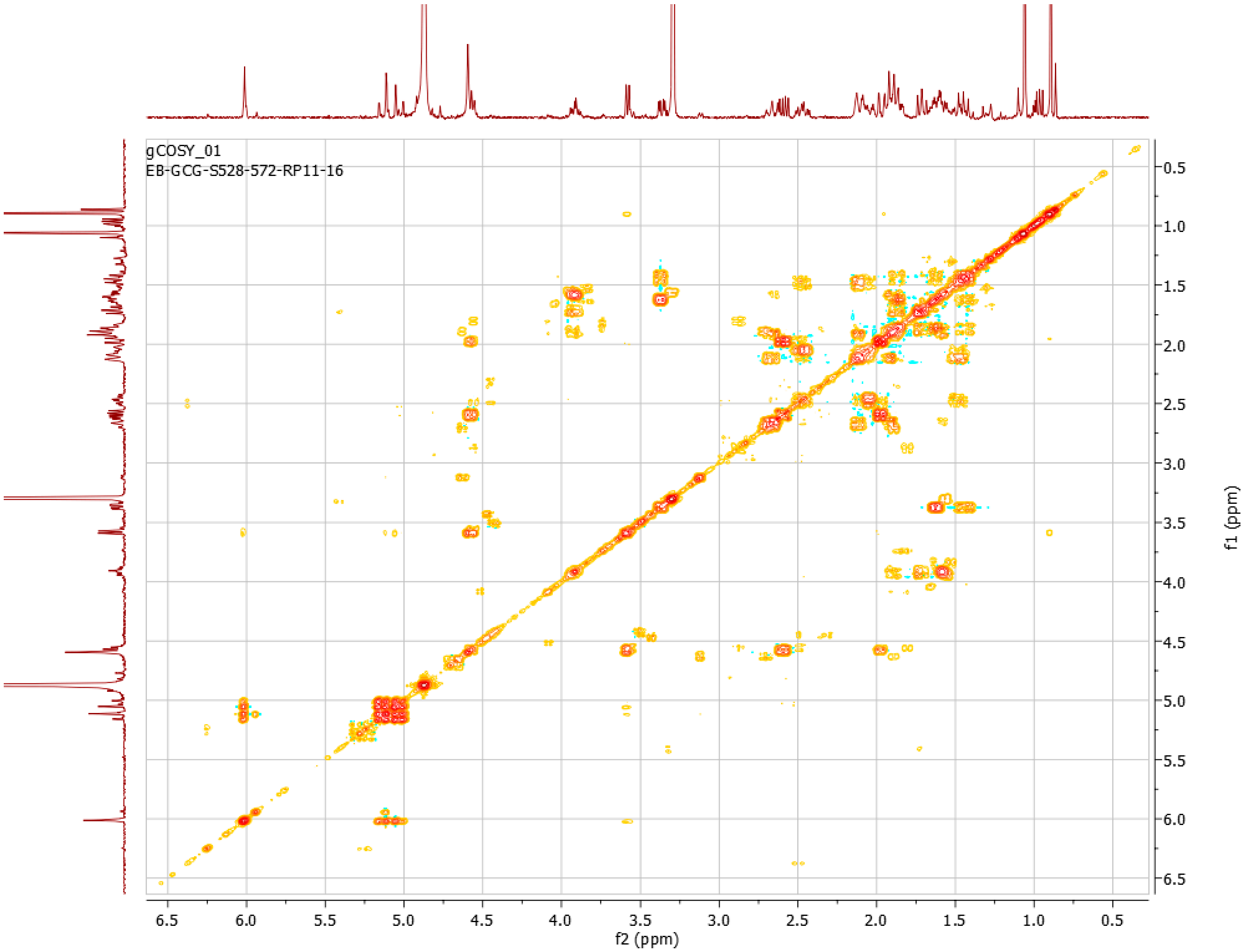

Figure S33. COSY Spectrum of 7(β),12(β)-dihdyroxy-3-oxo-gitoxigenin (**compound 3**)

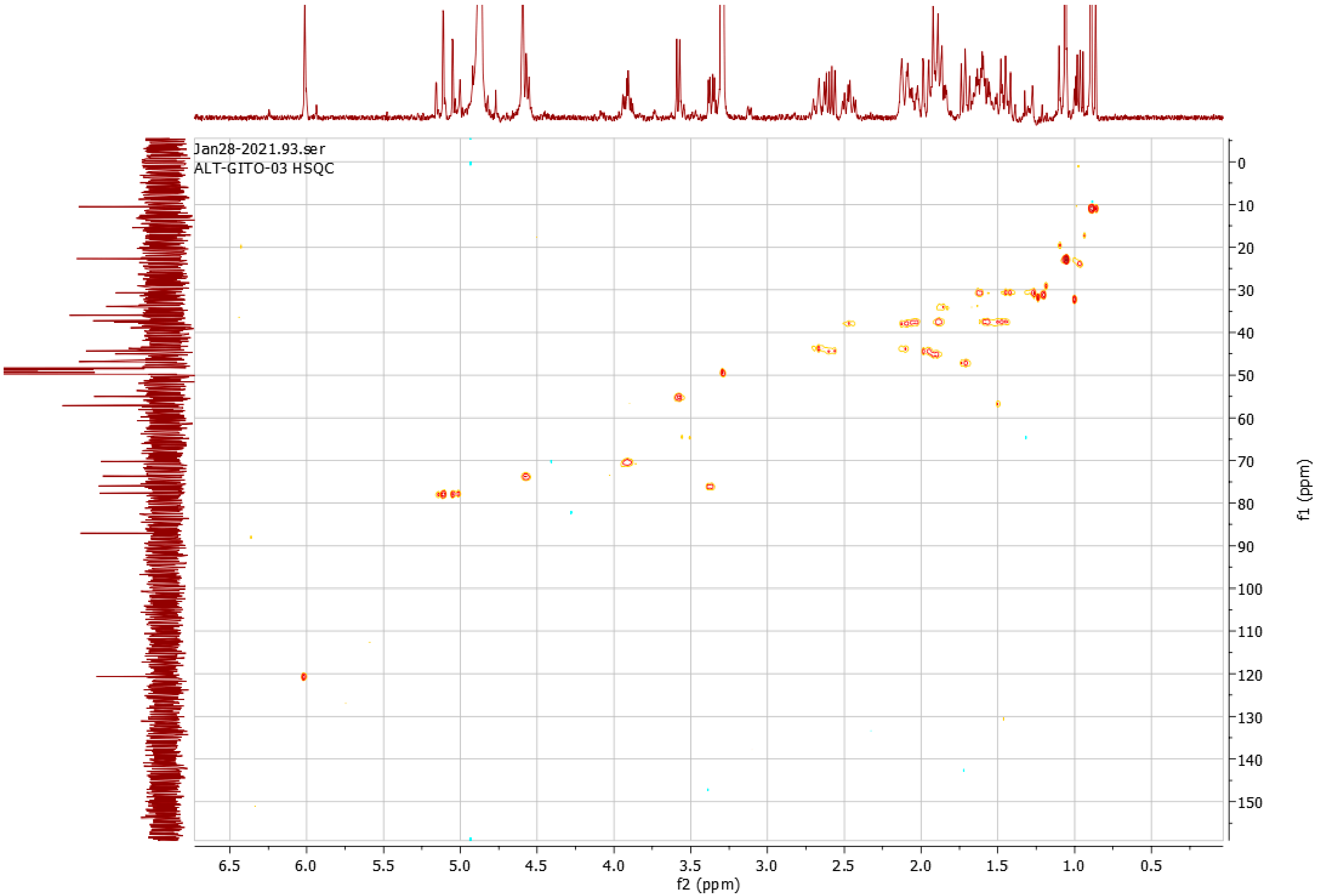

Figure S34. HSQC Spectrum of 7(β),12(β)-dihdyroxy-3-oxo-gitoxigenin (**compound 3**)

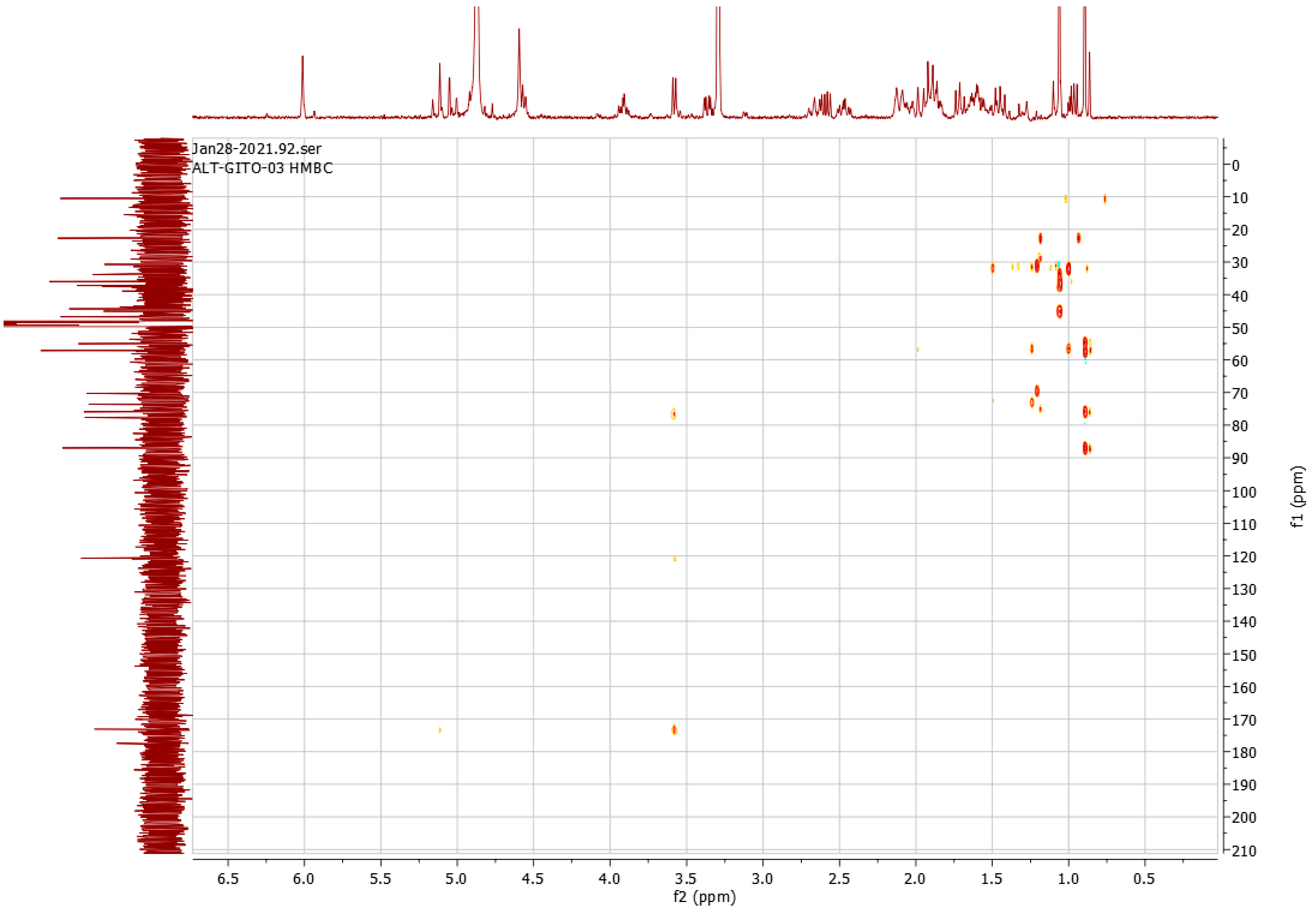

Figure S35. HMBC Spectrum of 7(*β*),12(*β*)-dihdyroxy-3-oxo-gitoxigenin (**compound 3**)

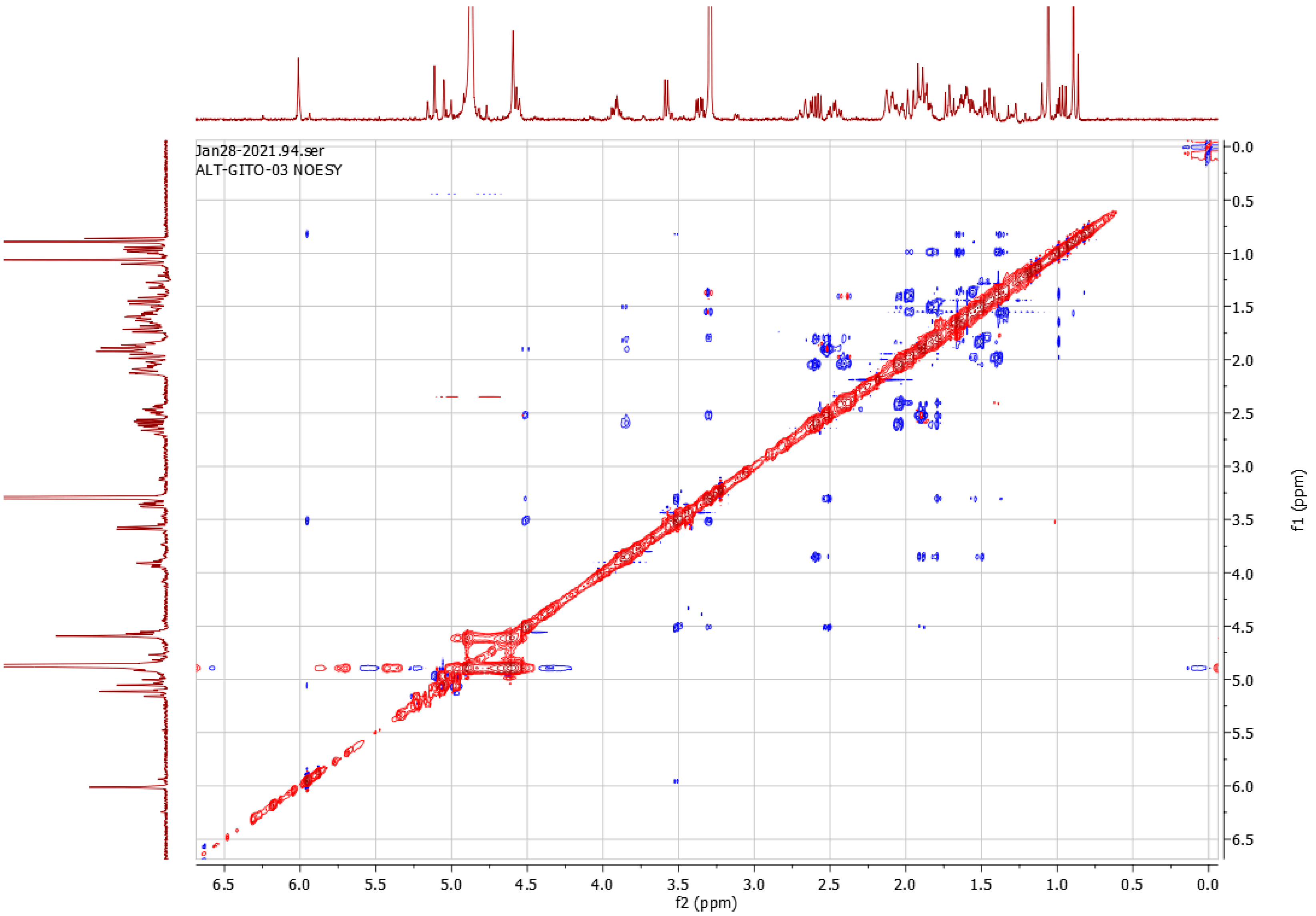

Figure S36. NOESY Spectrum of 7(β),12(β)-dihdyroxy-3-oxo-gitoxigenin (**compound 3**)

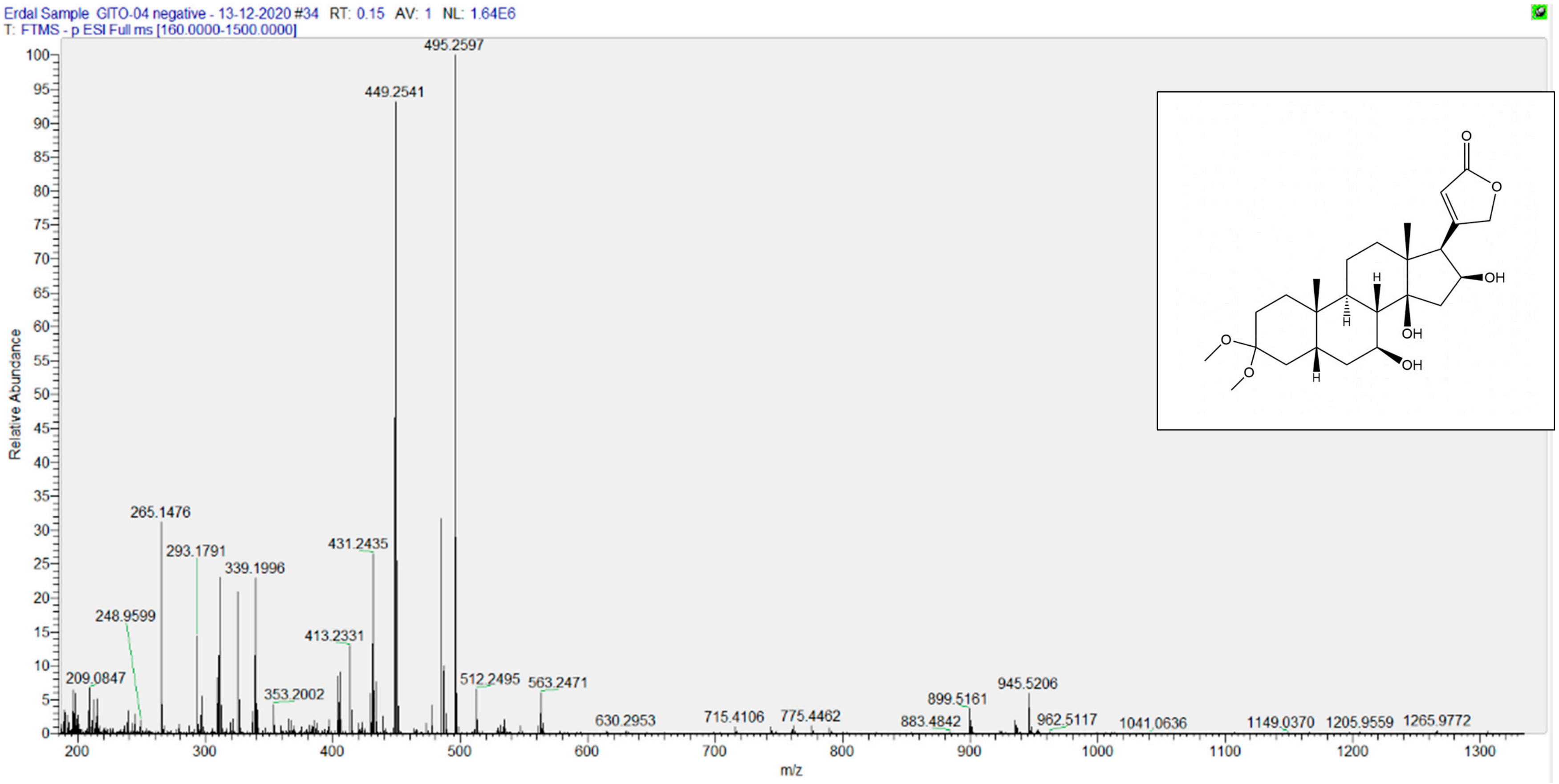

Figure S37. Negative-ion mode HR-ESI-MS spectrum of 3-dimethylacetal-7(β)-hydroxygitoxigenin (**compound 4**)

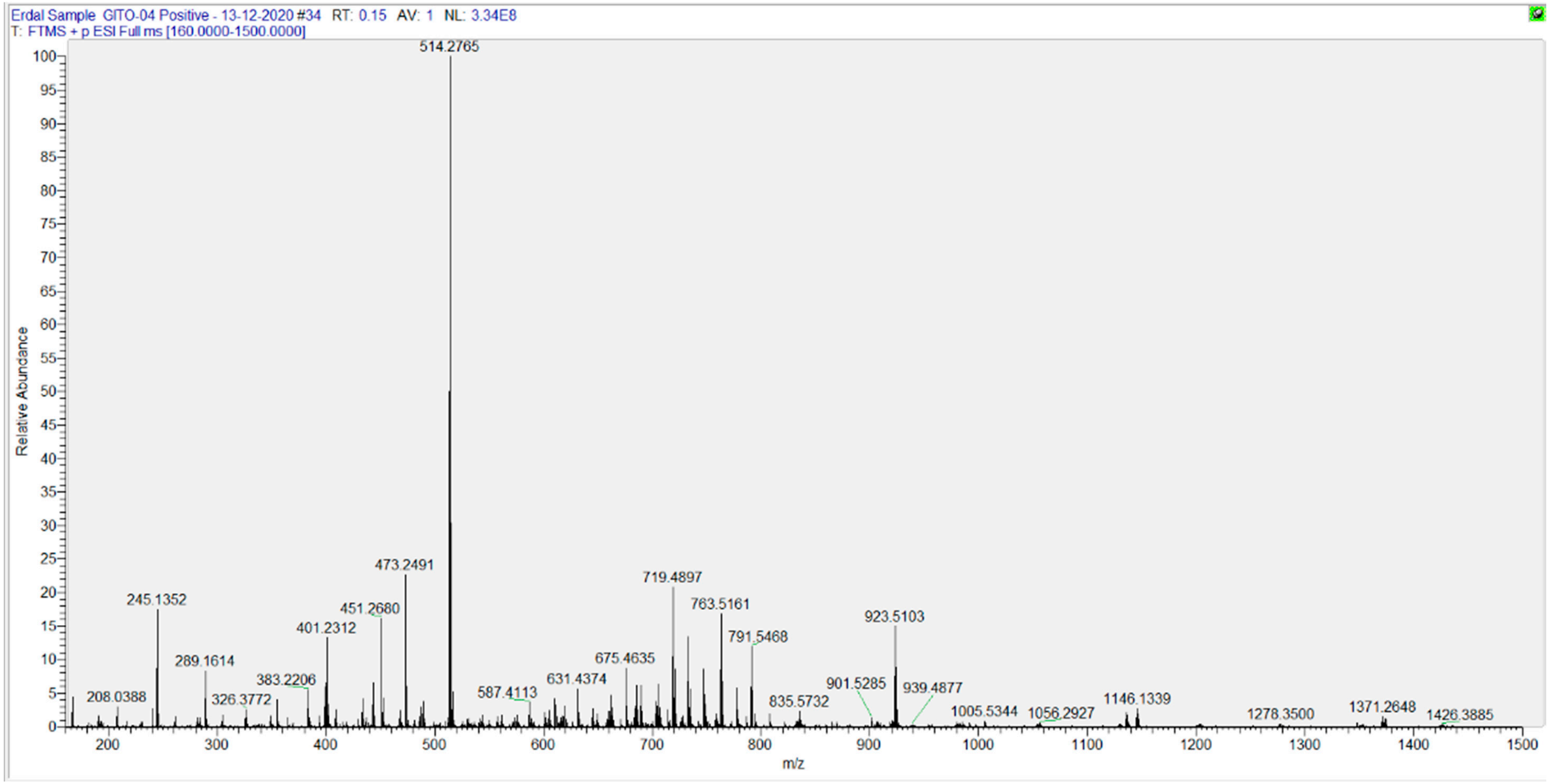

Figure S38. Positive-ion mode HR-ESI-MS spectrum of 3-dimethylacetal-7(β)-hydroxygitoxigenin (**compound 4**)

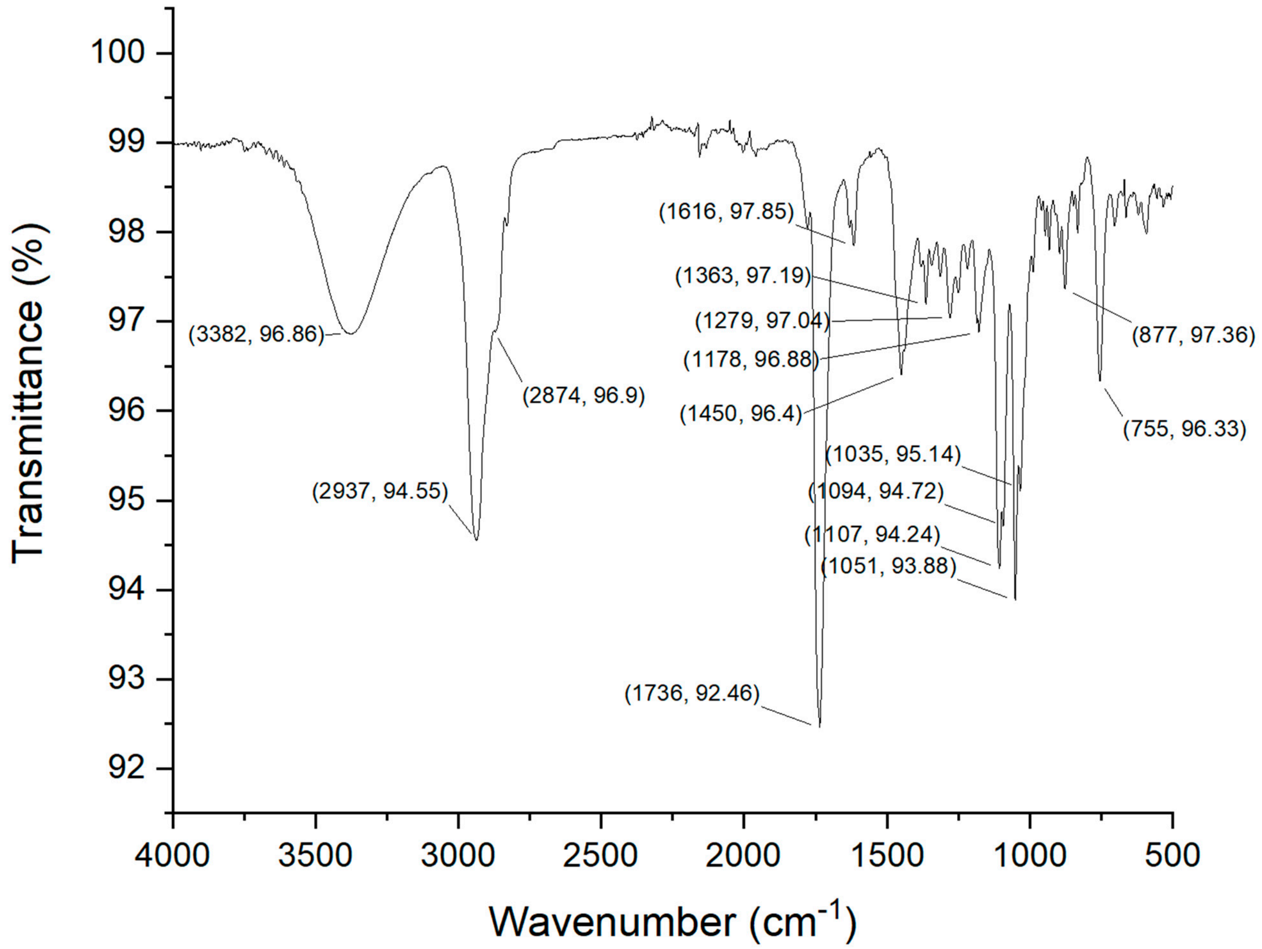

Figure S39. FT-IR spectrum of 3-dimethylacetal-7(*β*)-hydroxygitoxigenin (**compound 4**)

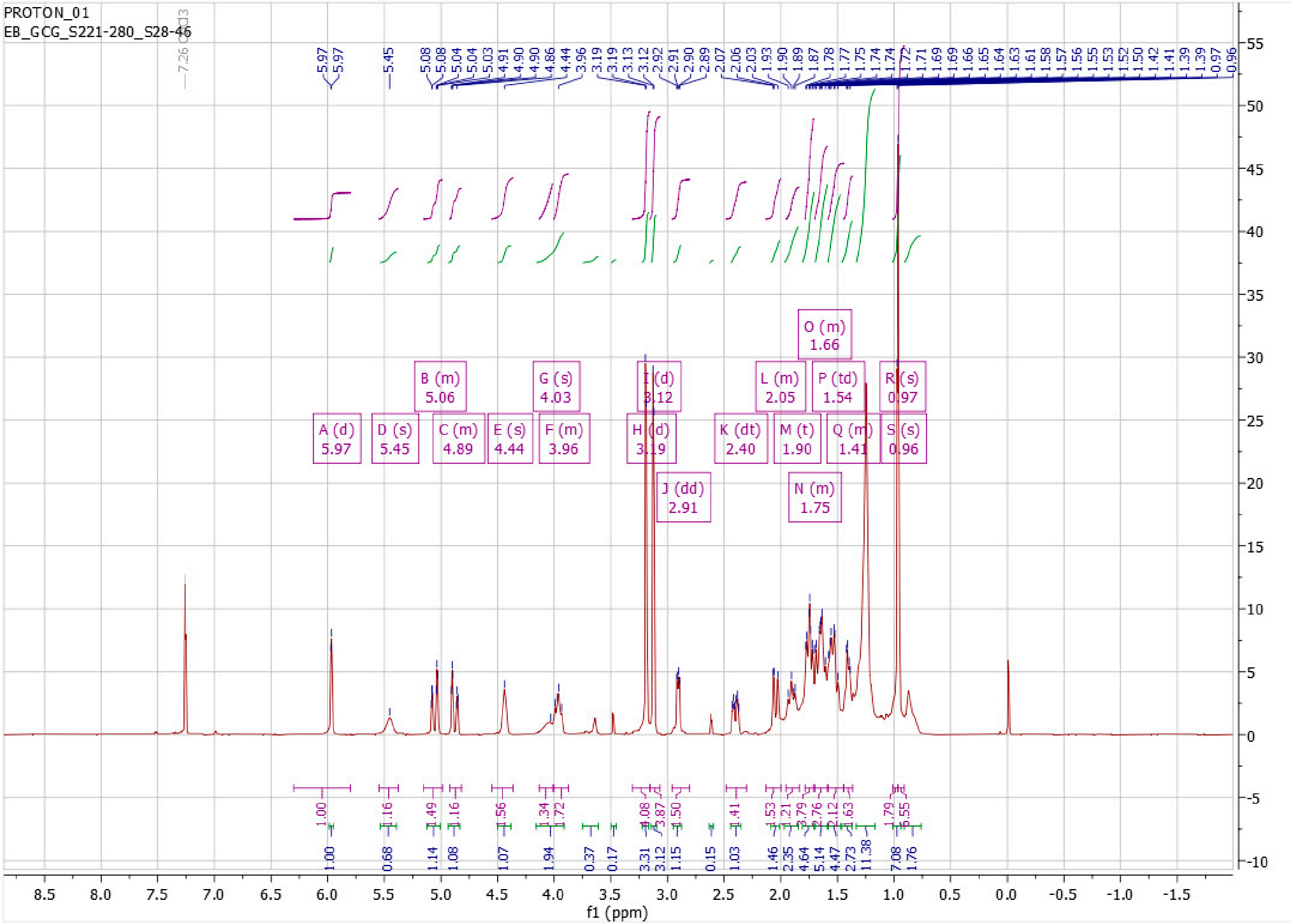

Figure S40. ^{1}H NMR Spectrum of 3-dimethylacetal-7(β)-hydroxygitoxigenin (**compound 4**)

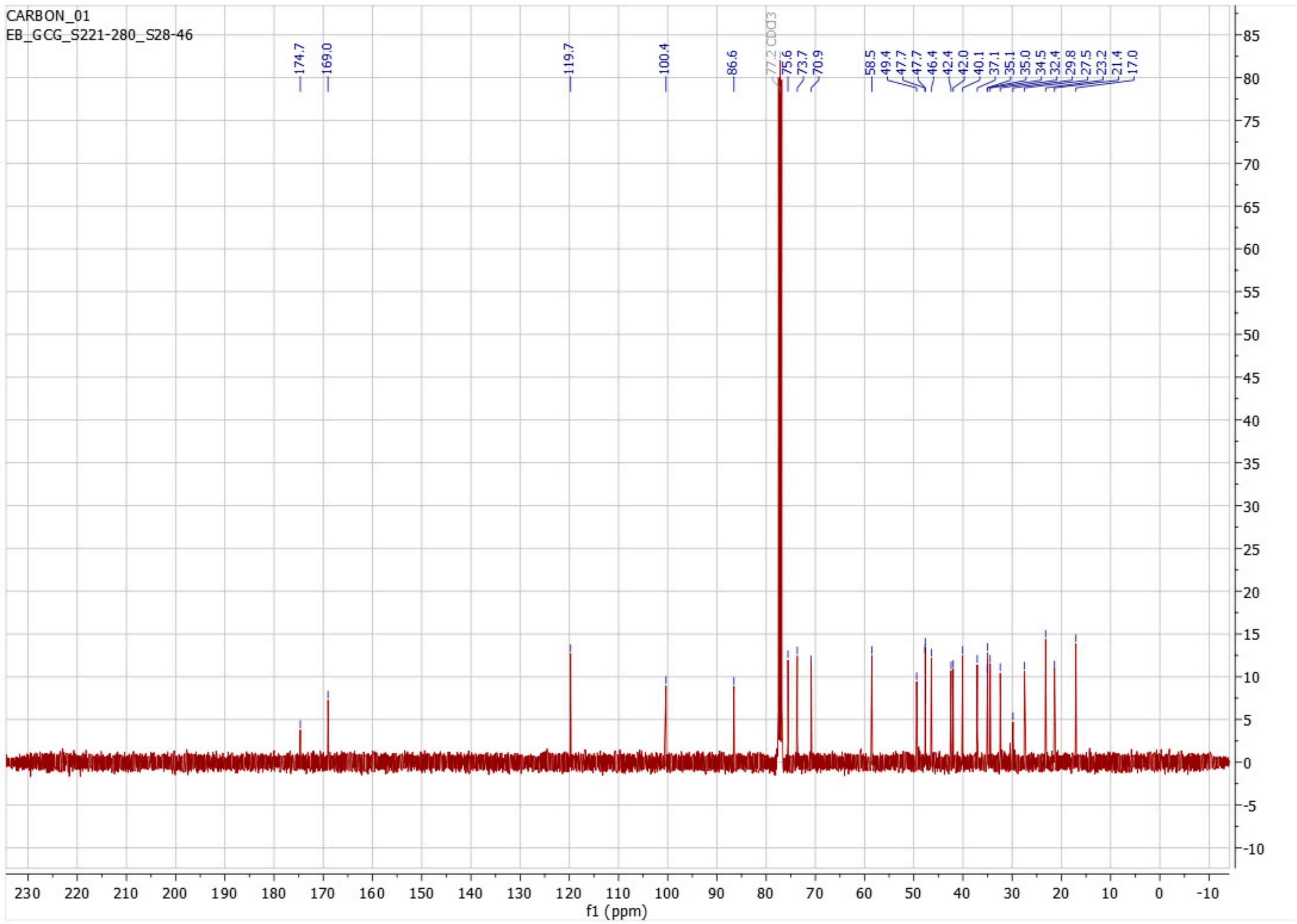

Figure S41. ^{13}C NMR Spectrum of 3-dimethylacetal-7(β)-hydroxygitoxigenin (**compound 4**)

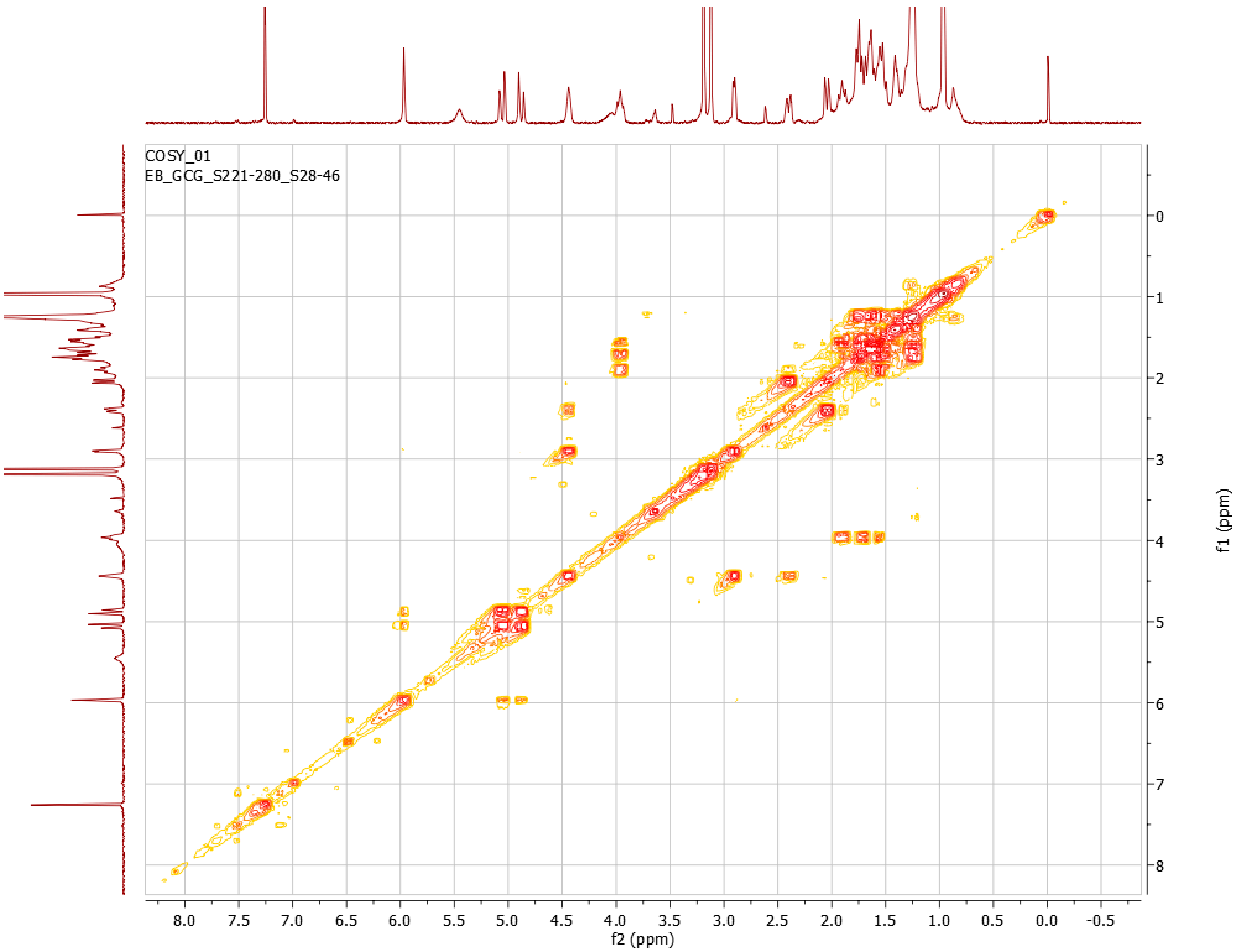

Figure S42. COSY Spectrum of 3-dimethylacetal-7(β)-hydroxygitoxigenin (**compound 4**)

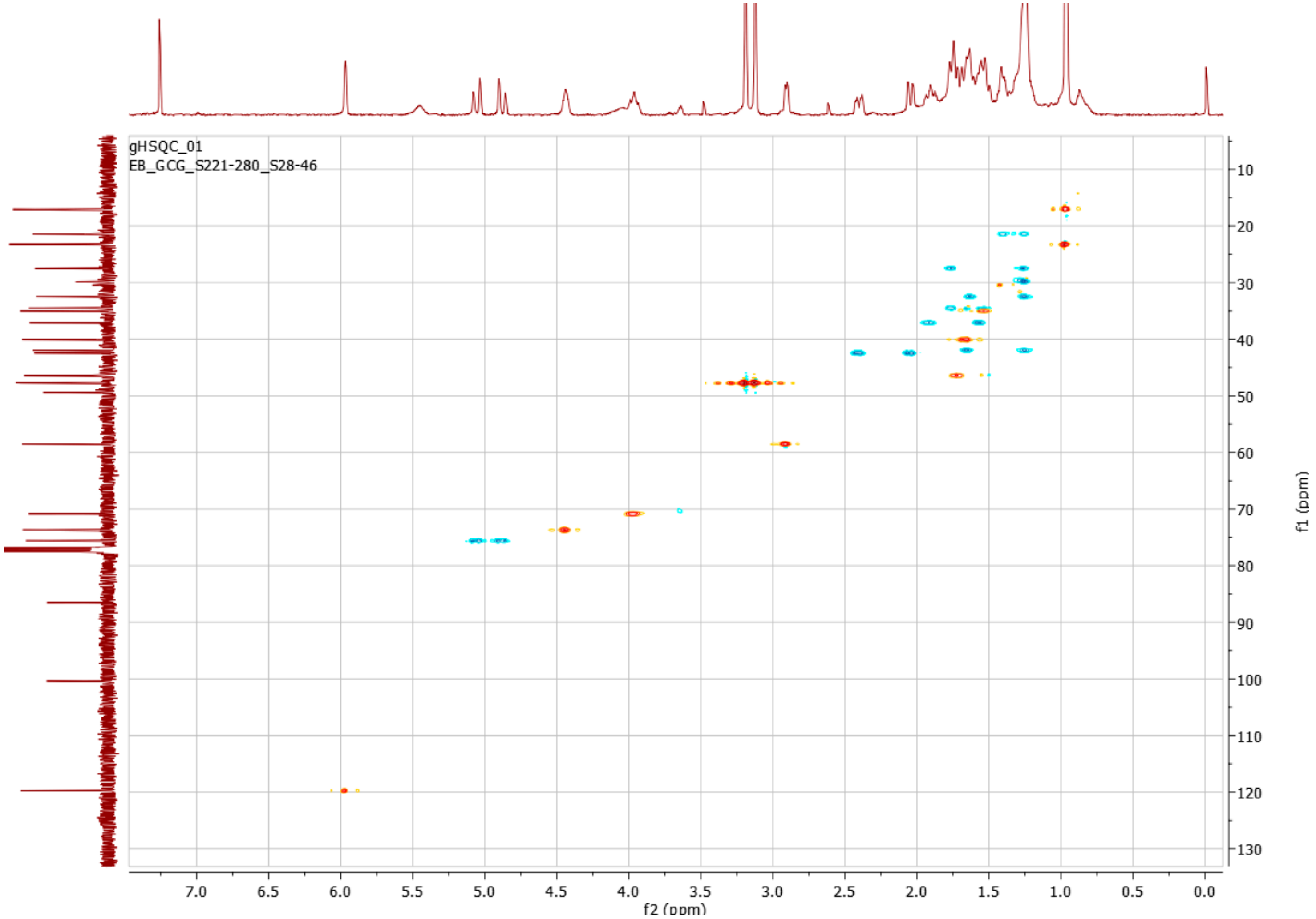

Figure S43. HSQC Spectrum of 3-dimethylacetal-7(β)-hydroxygitoxigenin (**compound 4**)

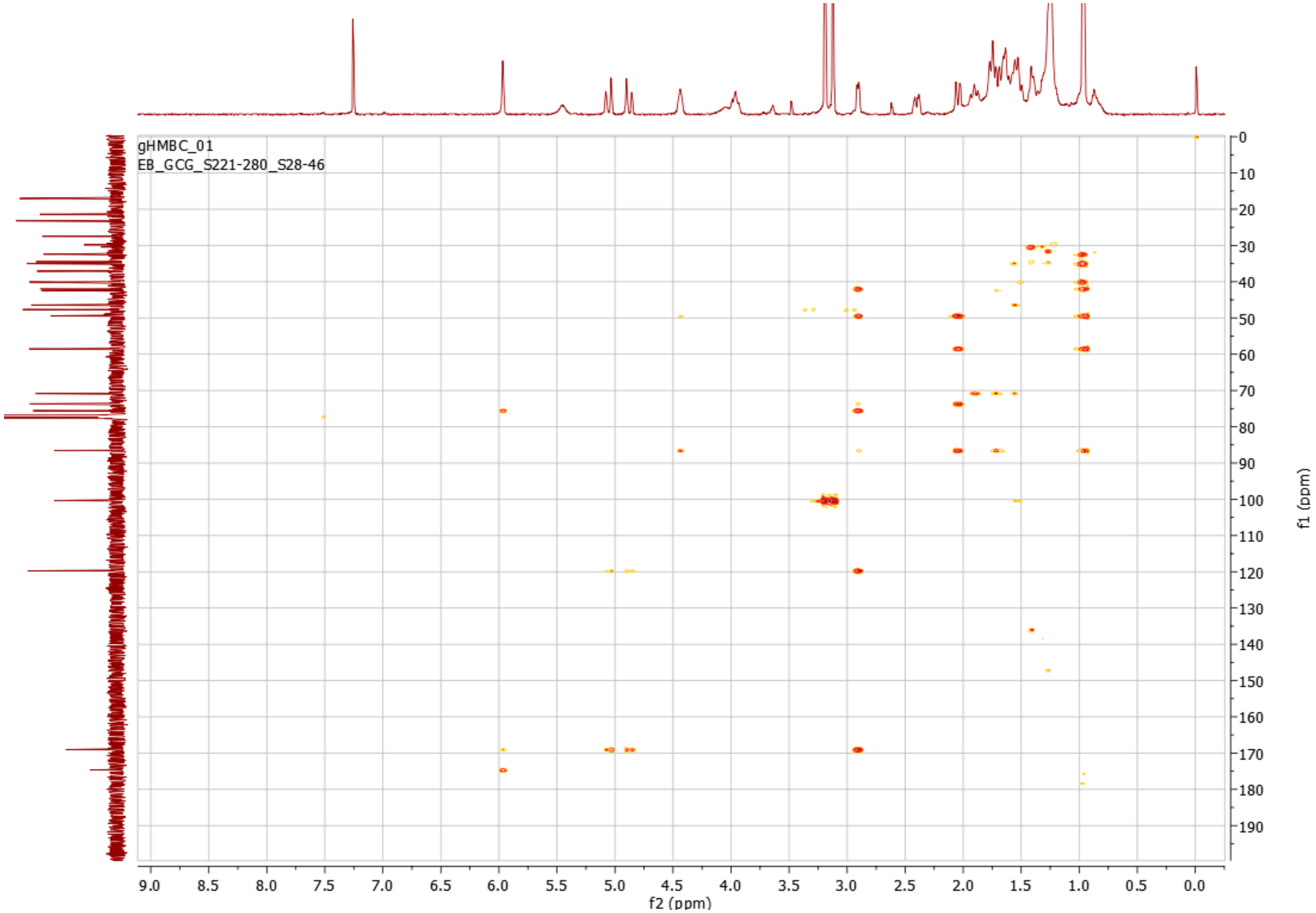

Figure S44. HMBC Spectrum of 3-dimethylacetal-7(β)-hydroxygitoxigenin (**compound 4**)

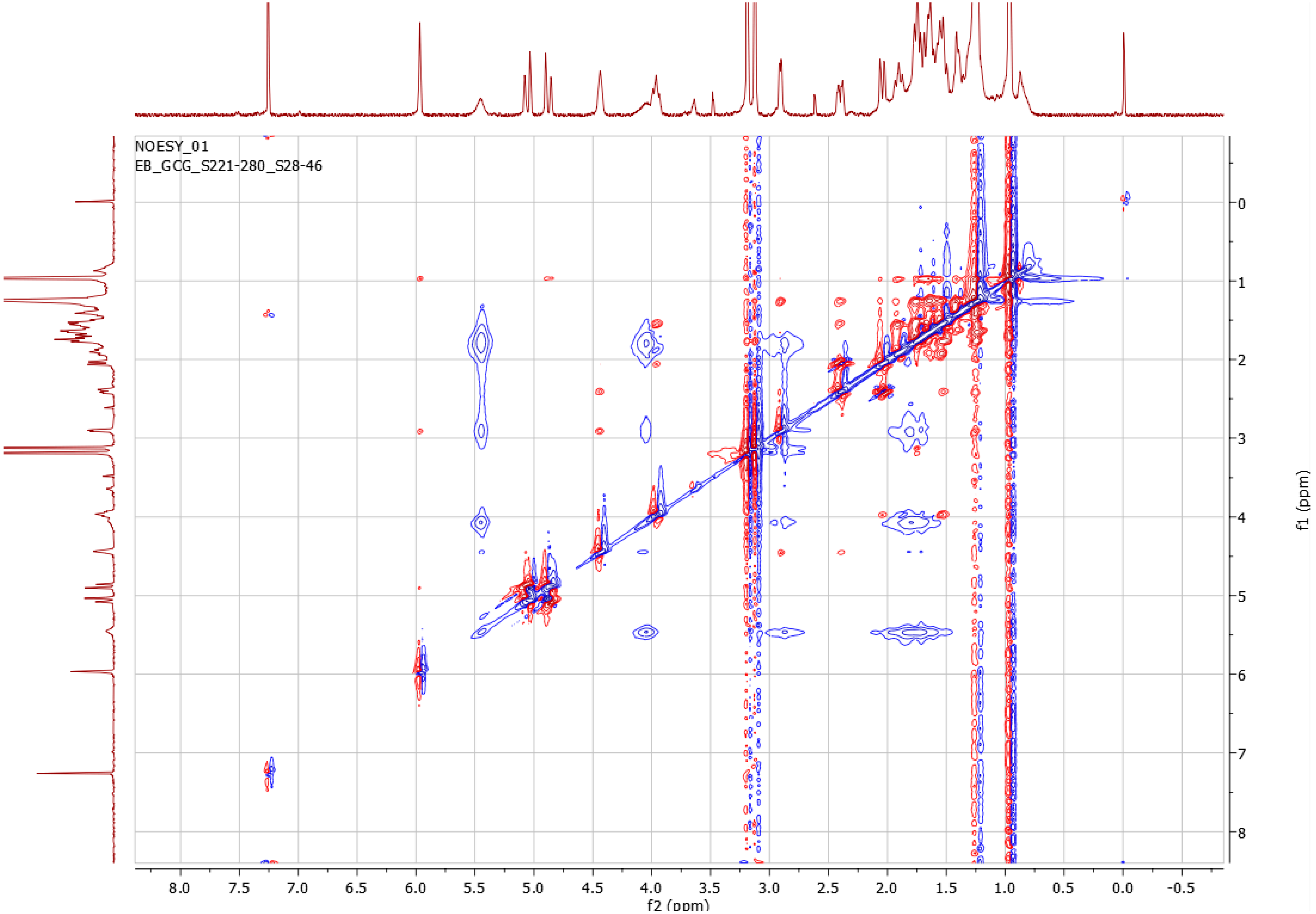

Figure S45. NOESY Spectrum of 3-dimethylacetal-7(β)-hydroxygitoxigenin (**compound 4**)

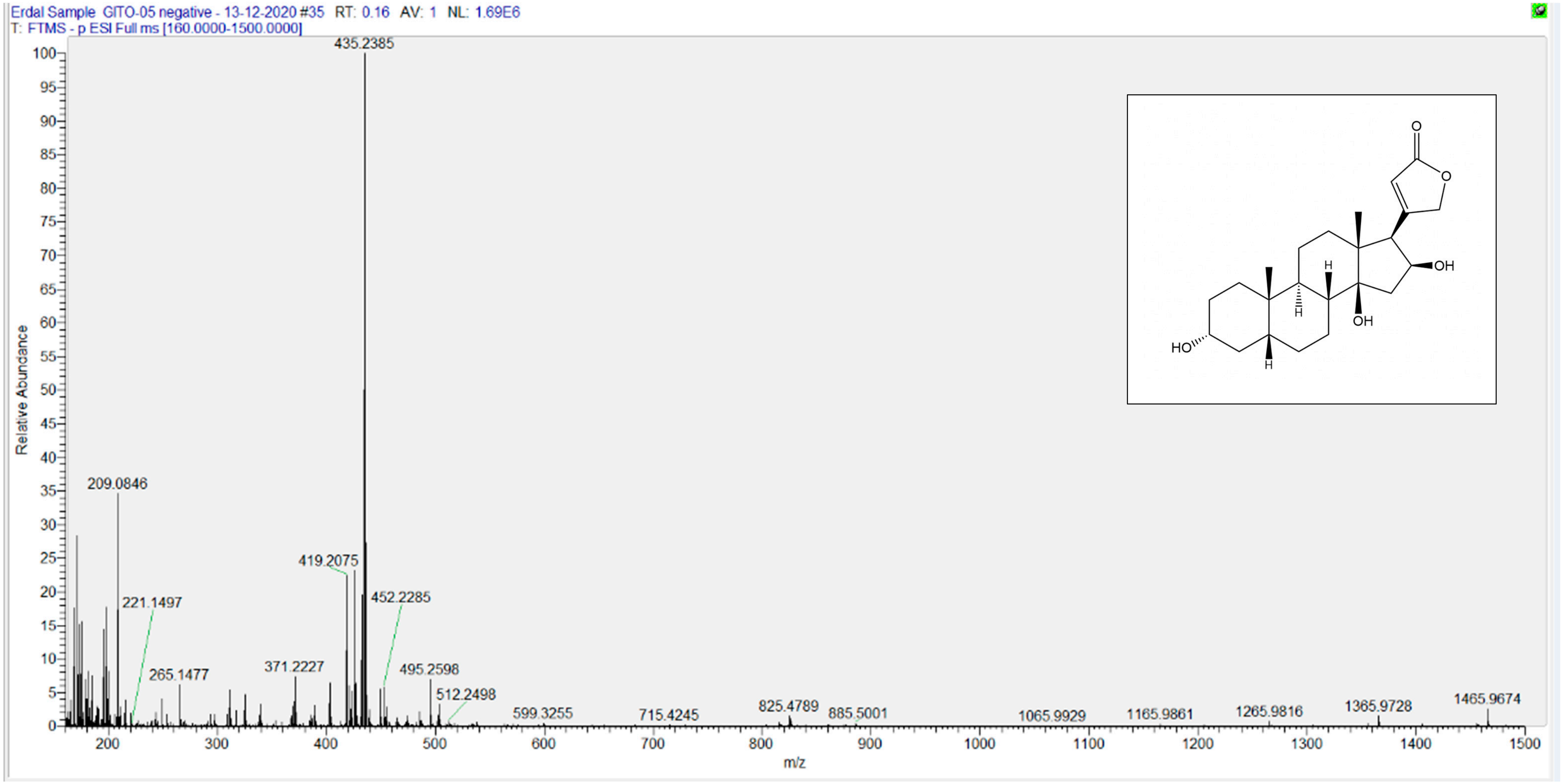

Figure S46. Negative-ion mode HR-ESI-MS spectrum of 3-epi-gitoxigenin (**compound 5**)

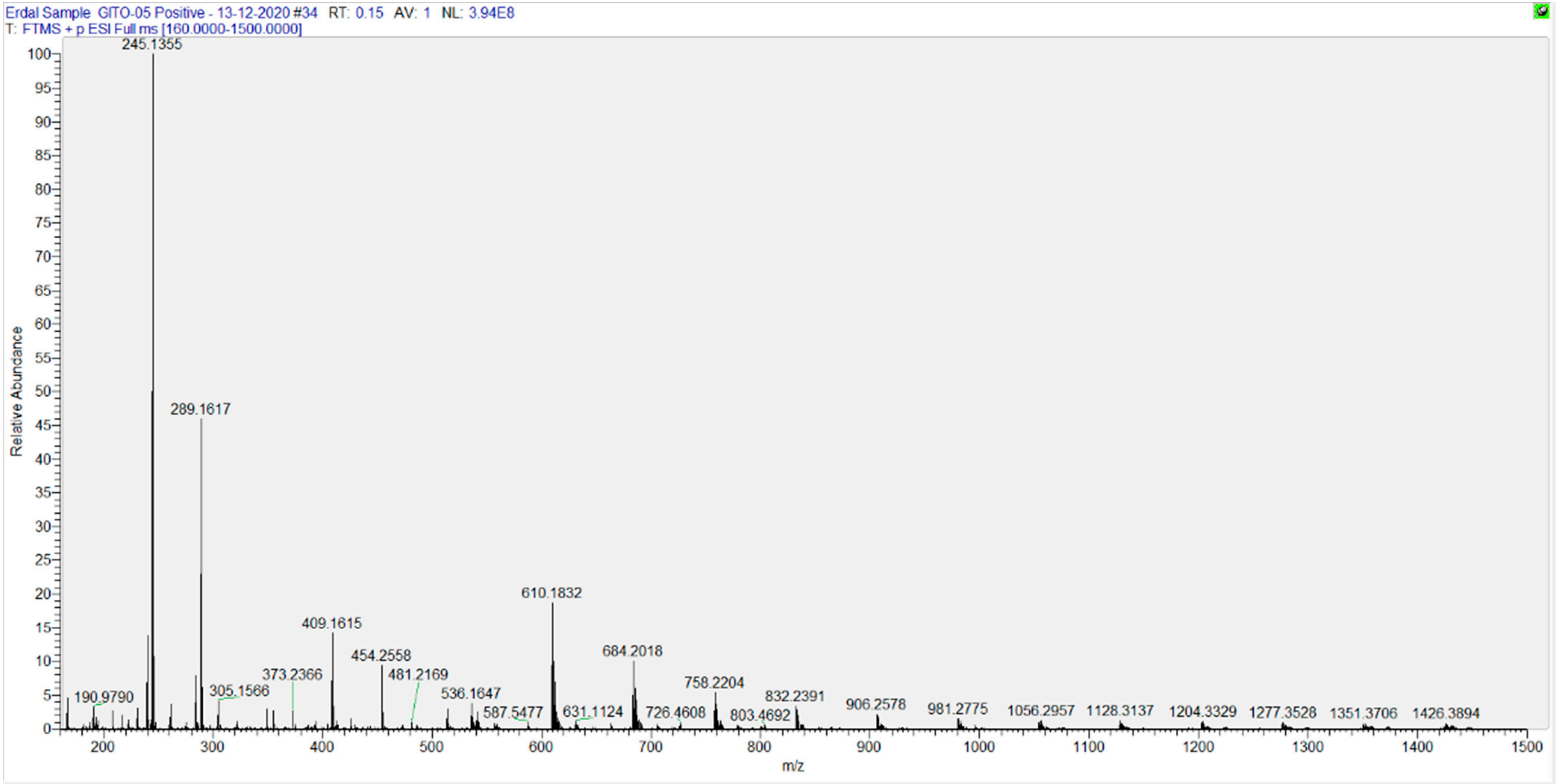

Figure S47. Positive-ion mode HR-ESI-MS spectrum of 3-epi-gitoxigenin (**compound 5**)

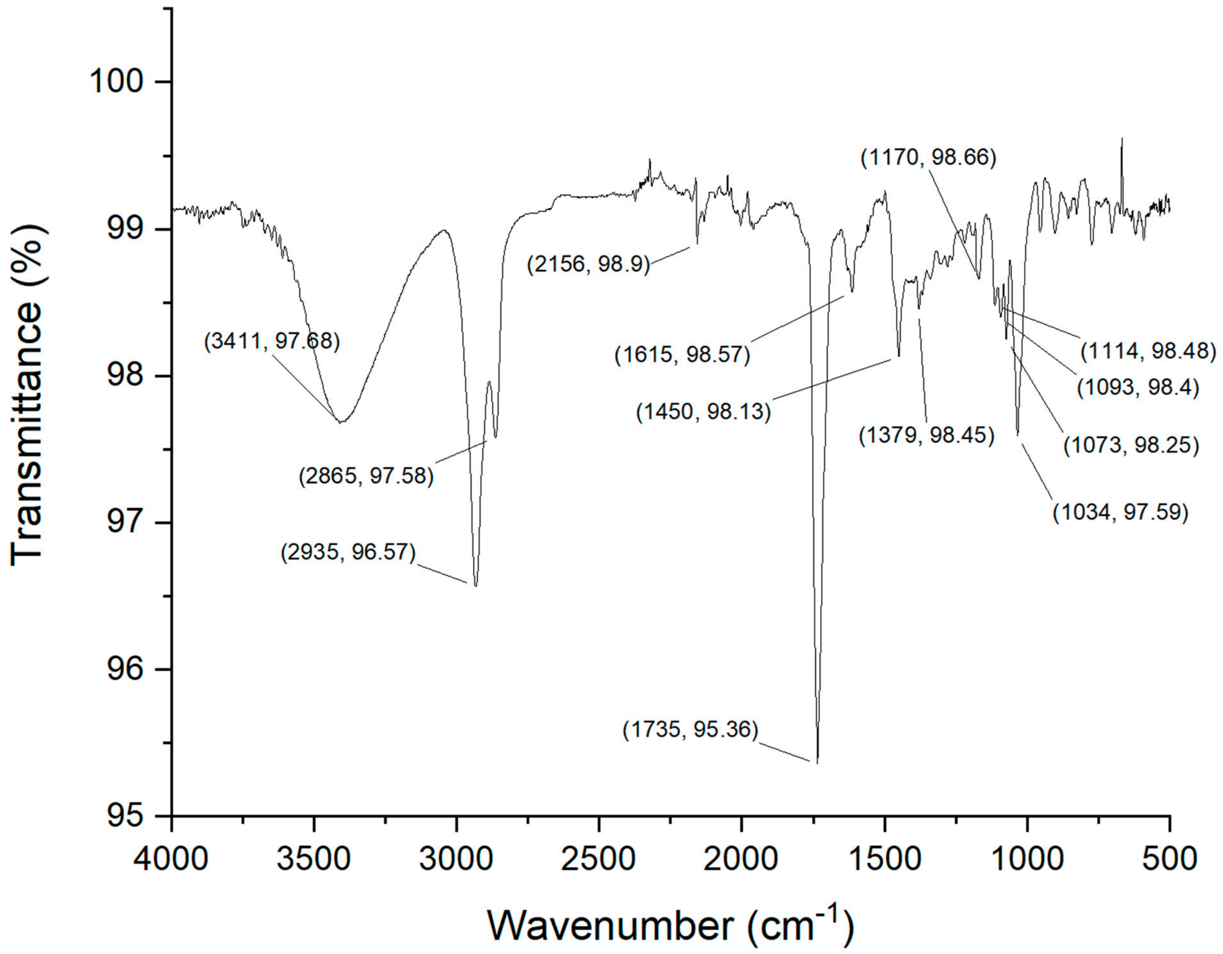

Figure S48. FT-IR Spectrum of 3-epi-gitoxigenin (**compound 5**)

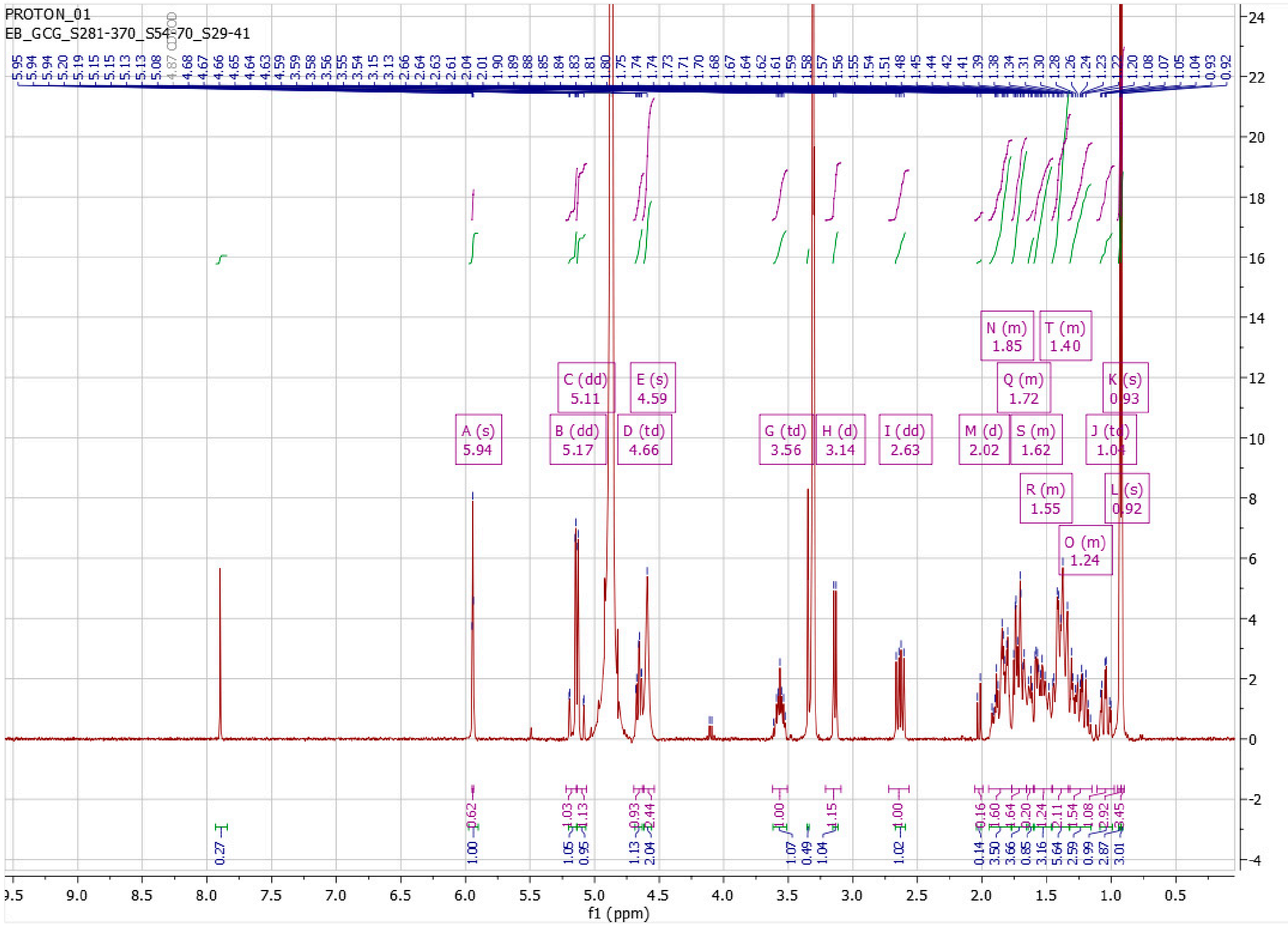

Figure S49. ^{1}H NMR Spectrum of 3-epi-gitoxigenin (**compound 5**)

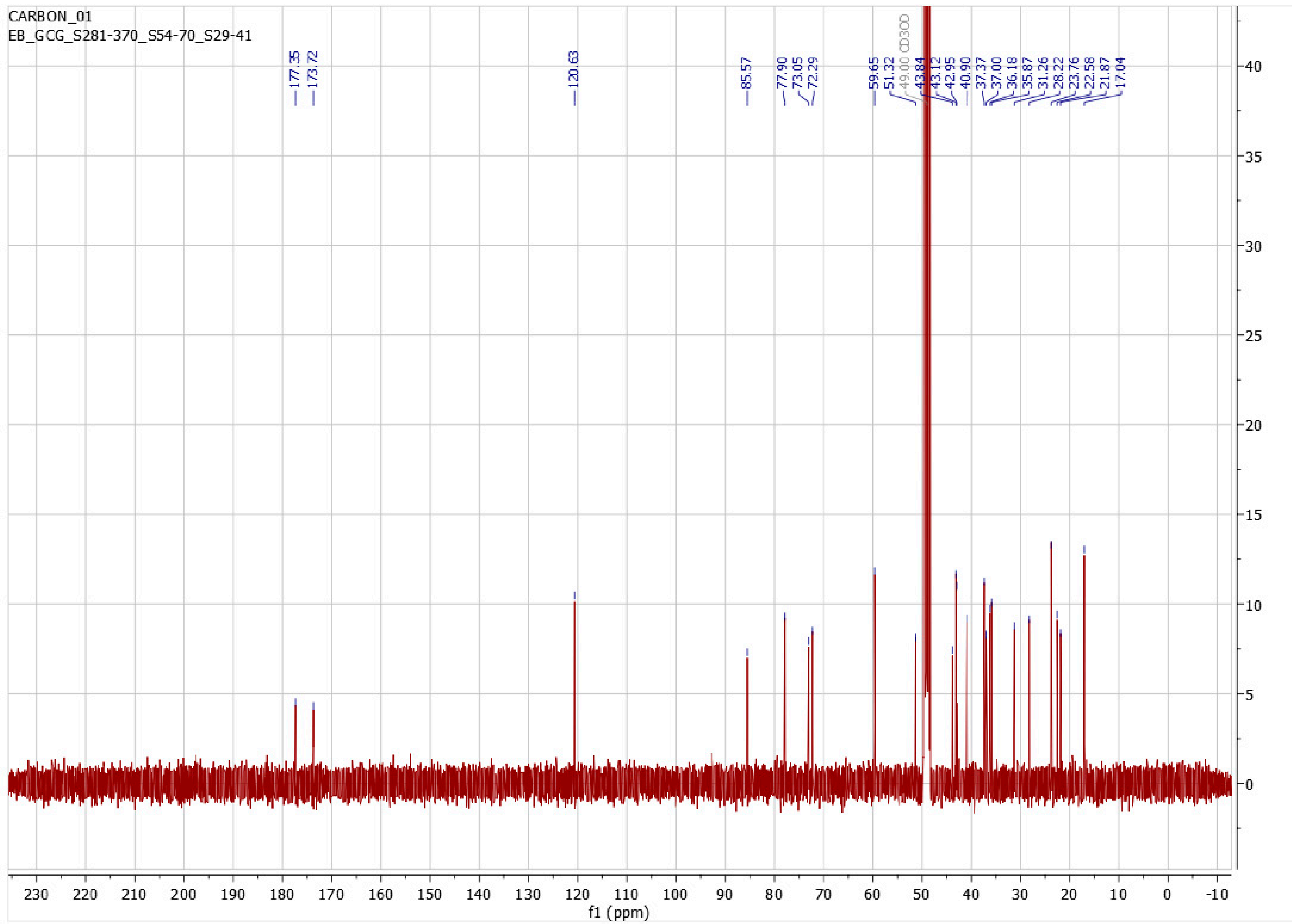

Figure S50. ^{13}C NMR Spectrum of 3-epi-gitoxigenin (**compound 5**)

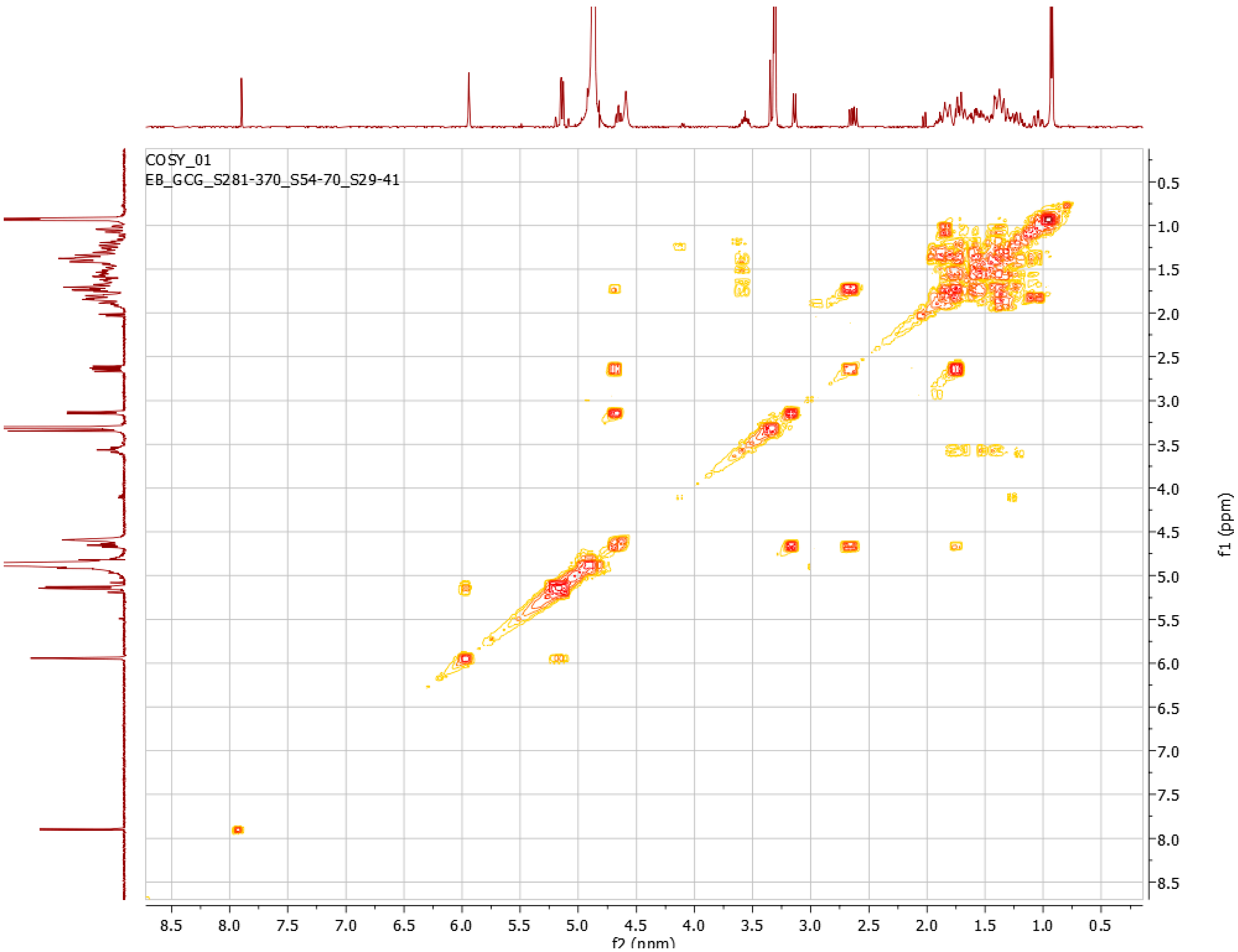

Figure S51. COSY Spectrum of 3-epi-gitoxigenin (**compound 5**)

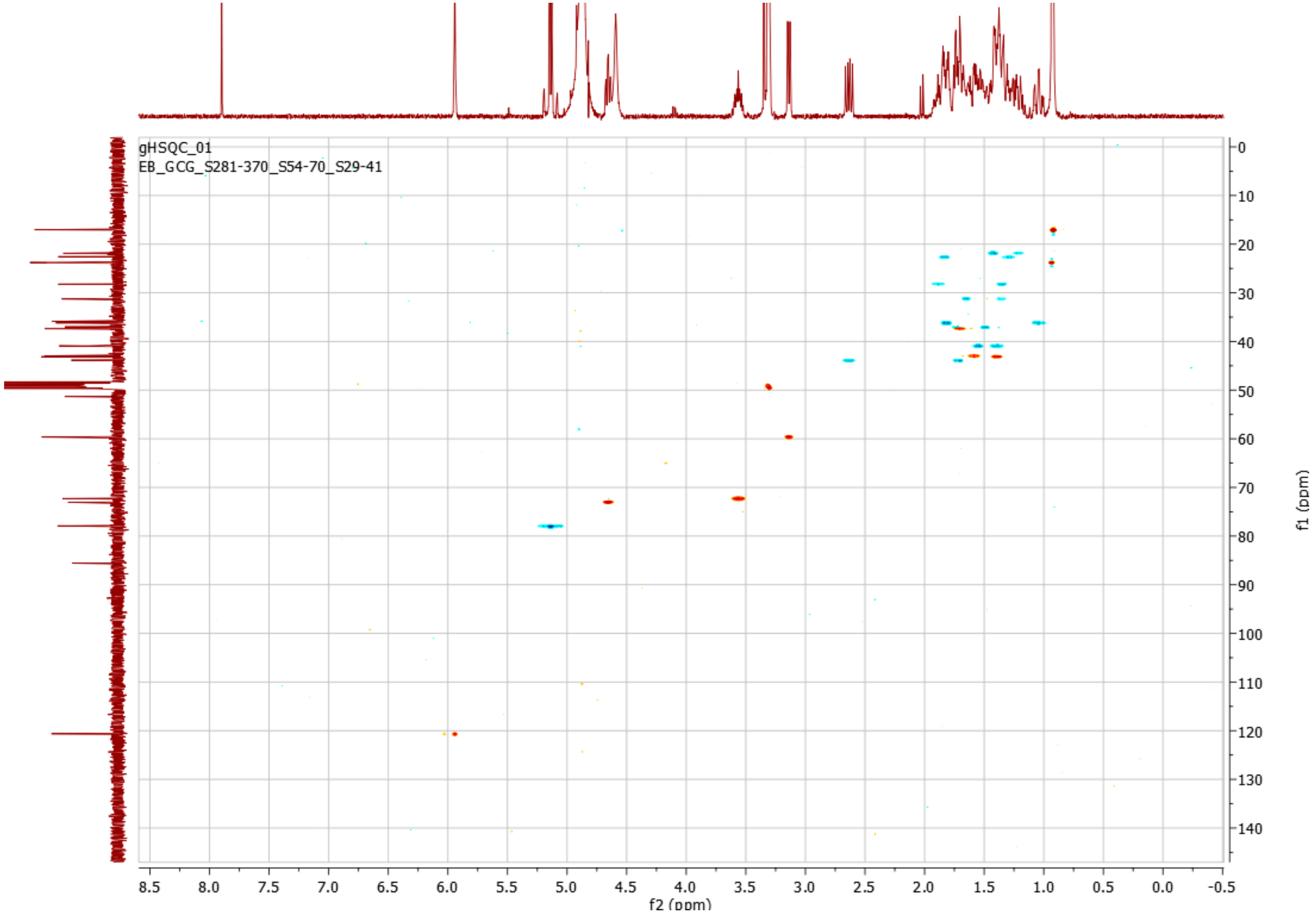

Figure S52. HSQC Spectrum of 3-epi-gitoxigenin (**compound 5**)

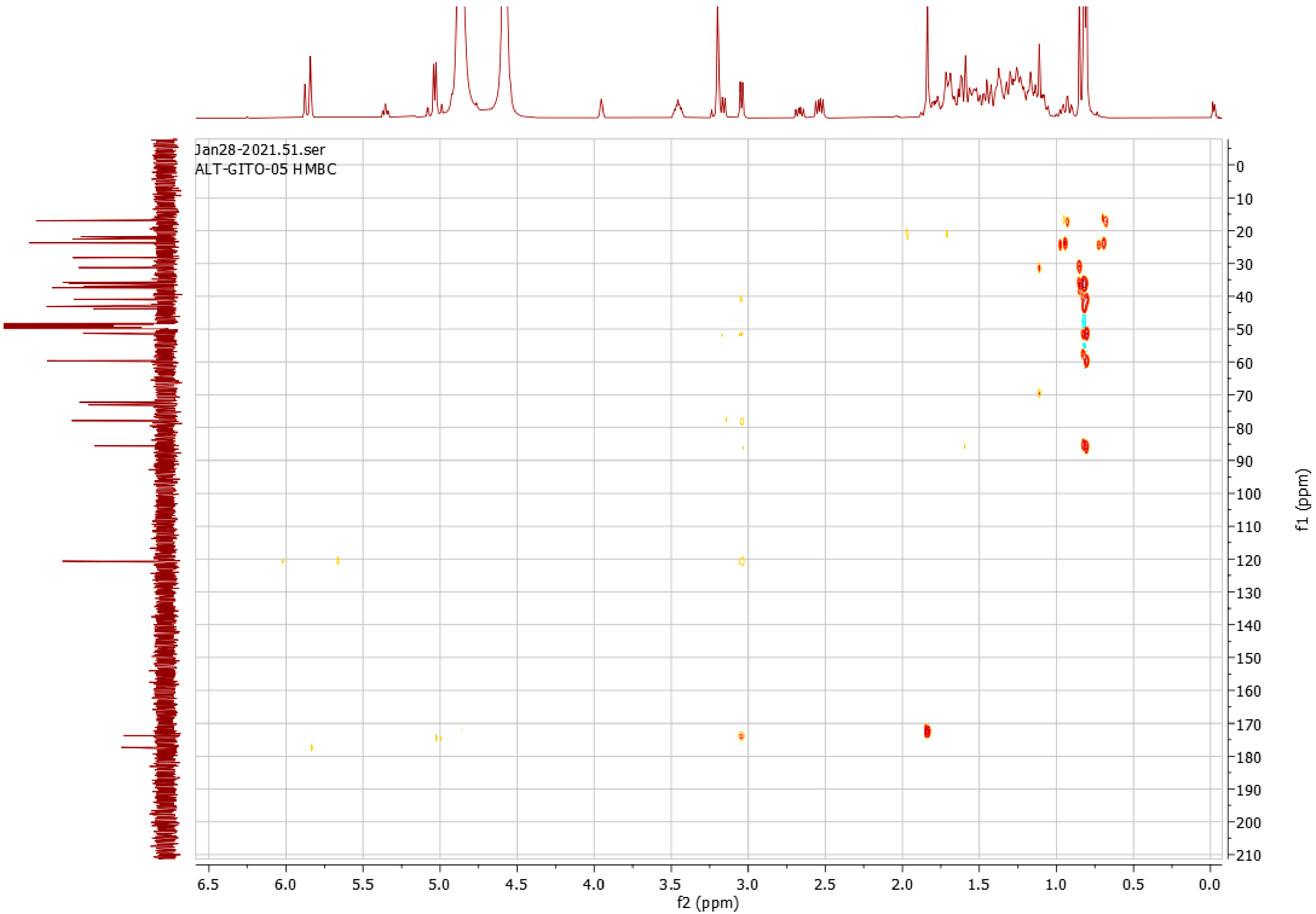

Figure S53. HMBC Spectrum of 3-epi-gitoxigenin (**compound 5**)

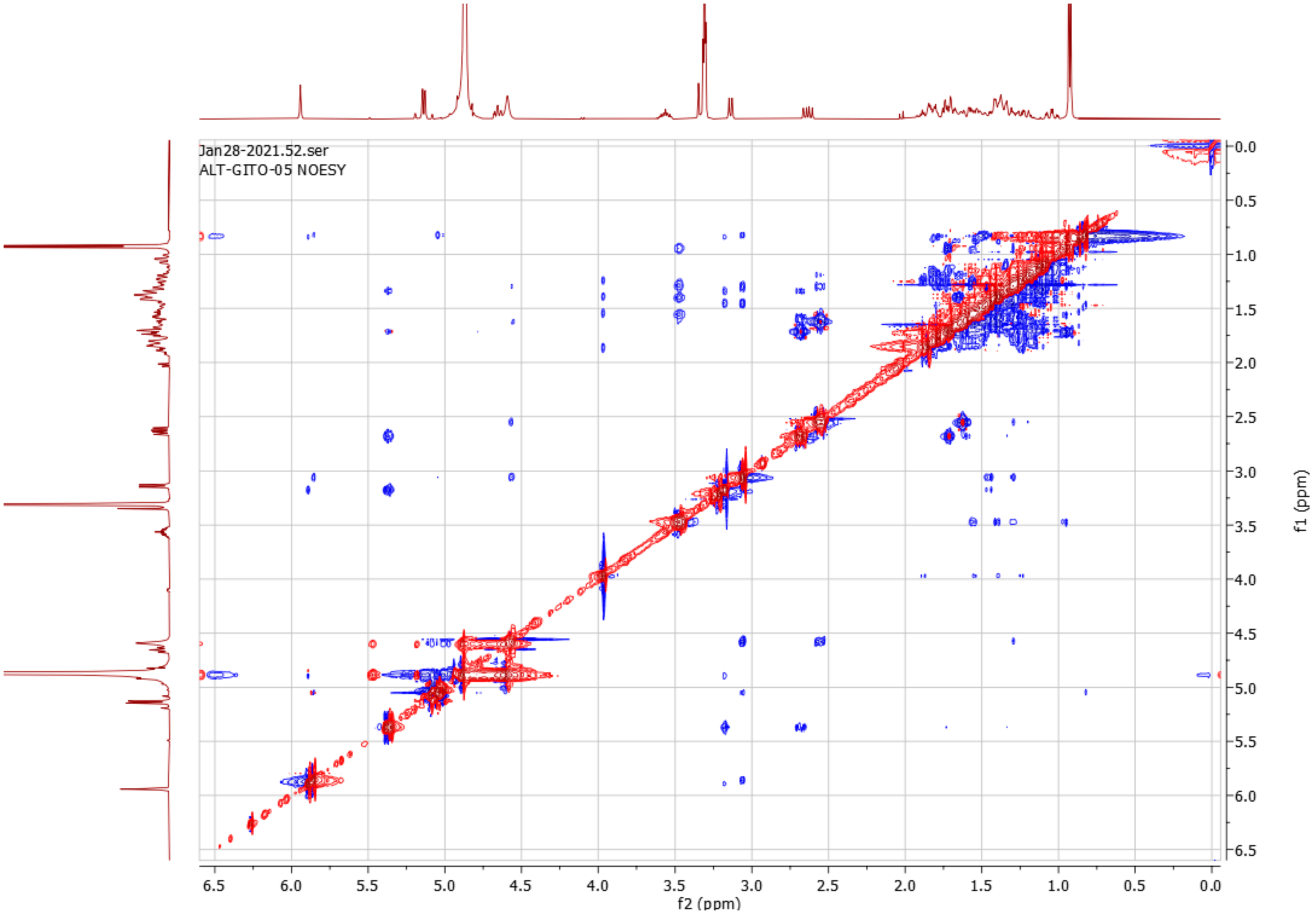

Figure S54. NOESY Spectrum of 3-epi-gitoxigenin (**compound 5**)

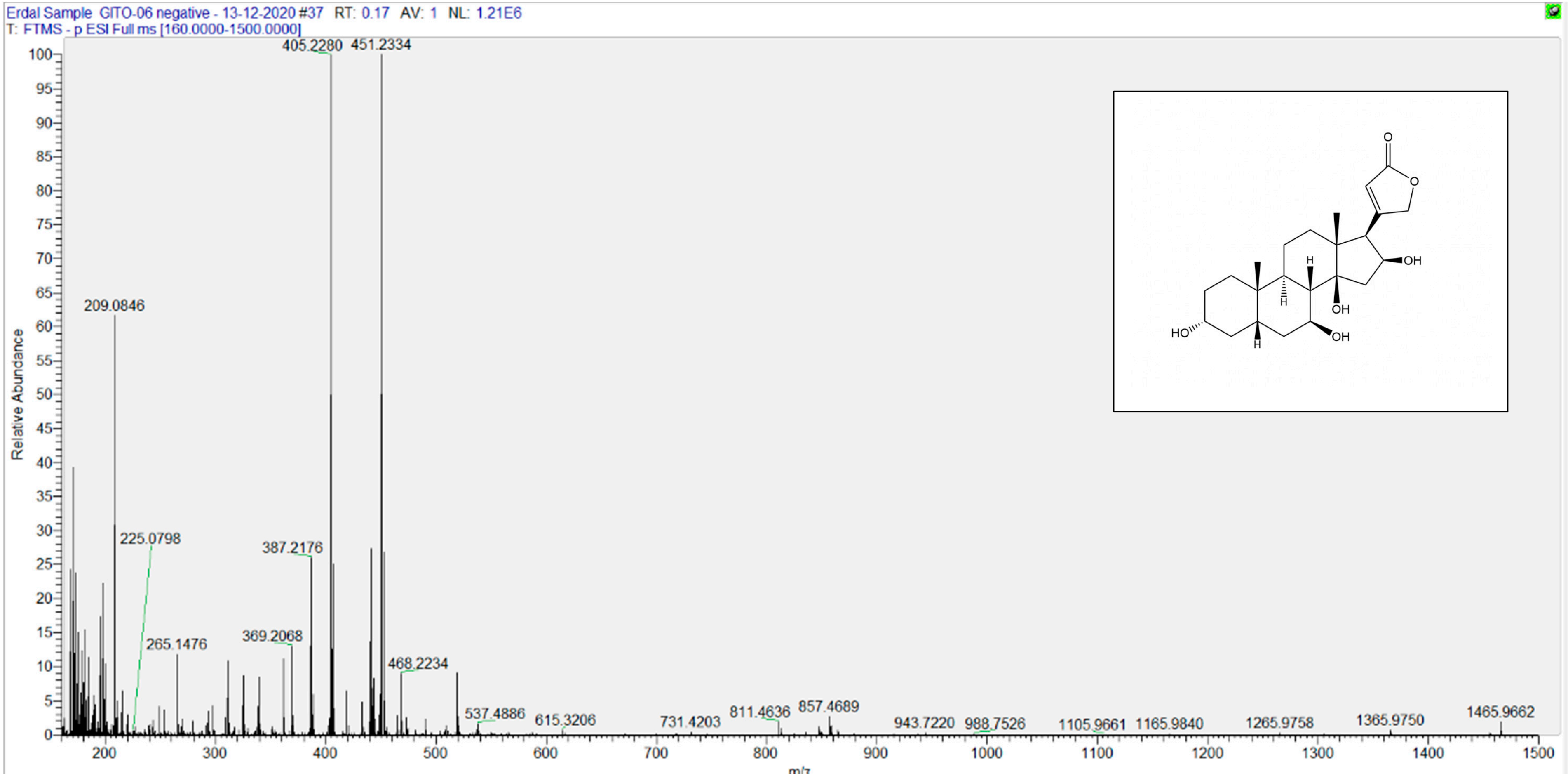

Figure S55. Negative-ion mode HR-ESI-MS spectrum of 7(β)-hydroxy-3-epi-gitoxigenin (**compound 6**)

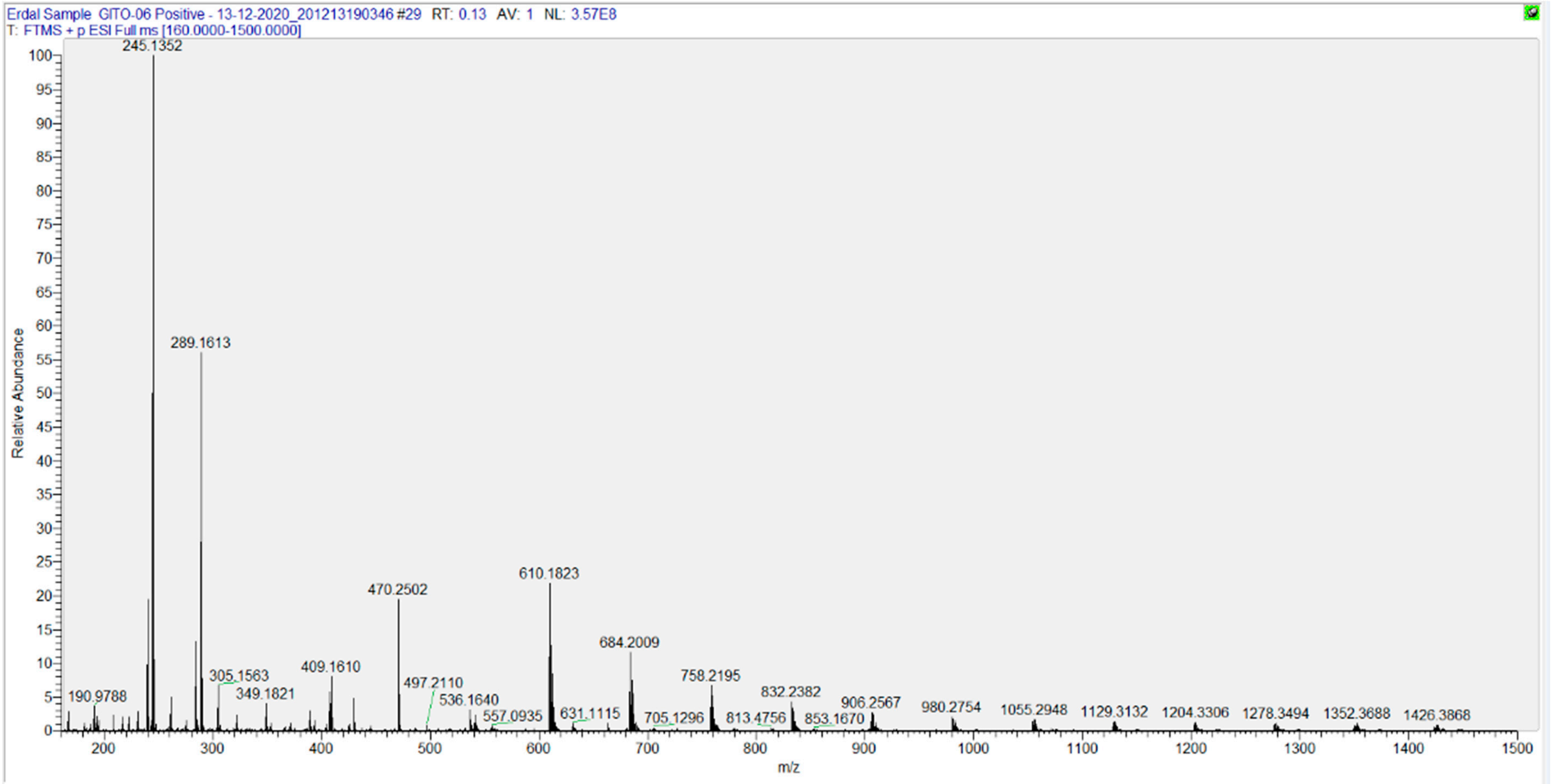

Figure S56. Positive-ion mode HR-ESI-MS spectrum of 7(β)-hydroxy-3-epi-gitoxigenin (**compound 6**)

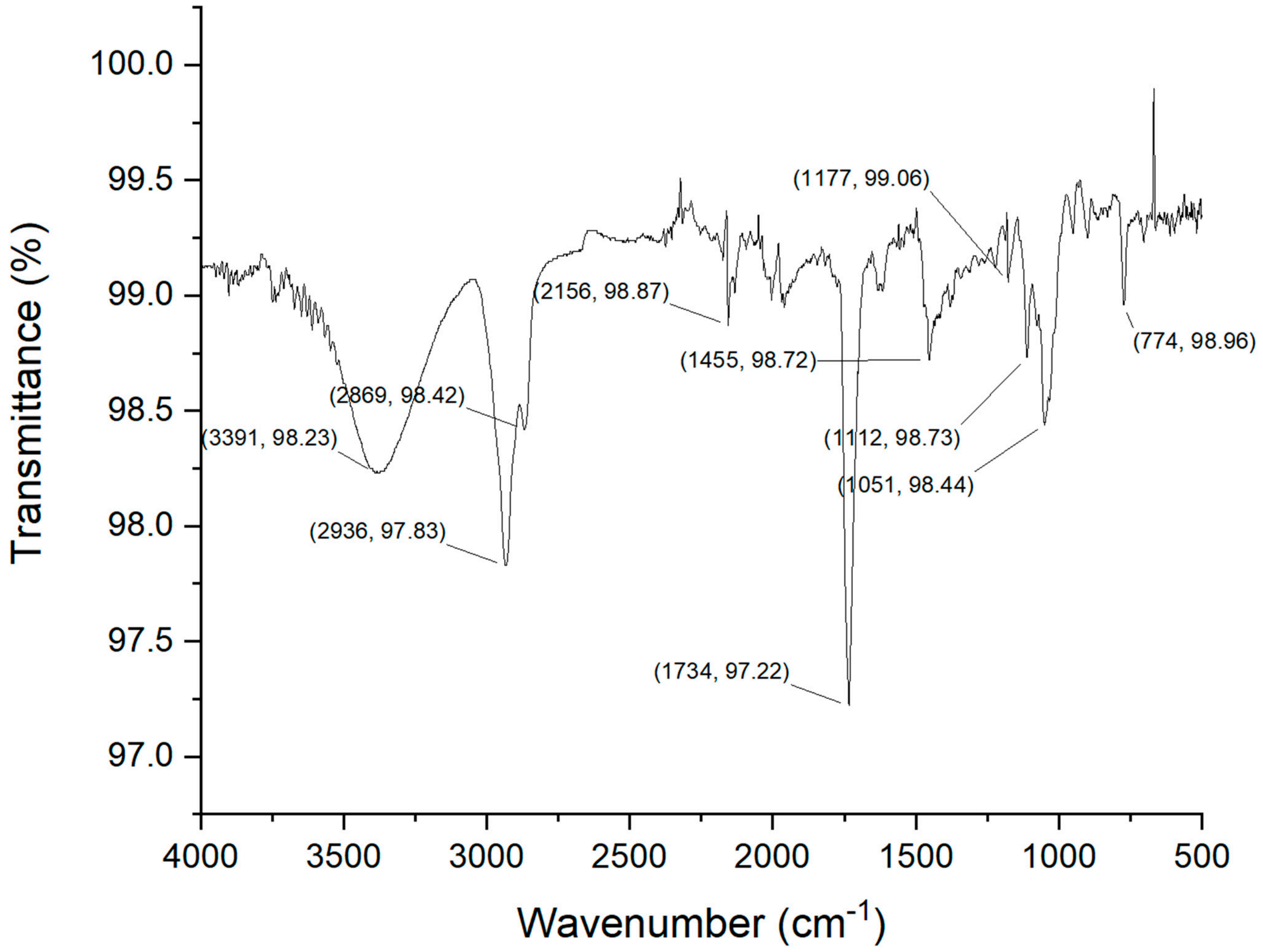

Figure S57: FT-IR Spectrum of 7(β)-hydroxy-3-epi-gitoxigenin (**compound 6**)

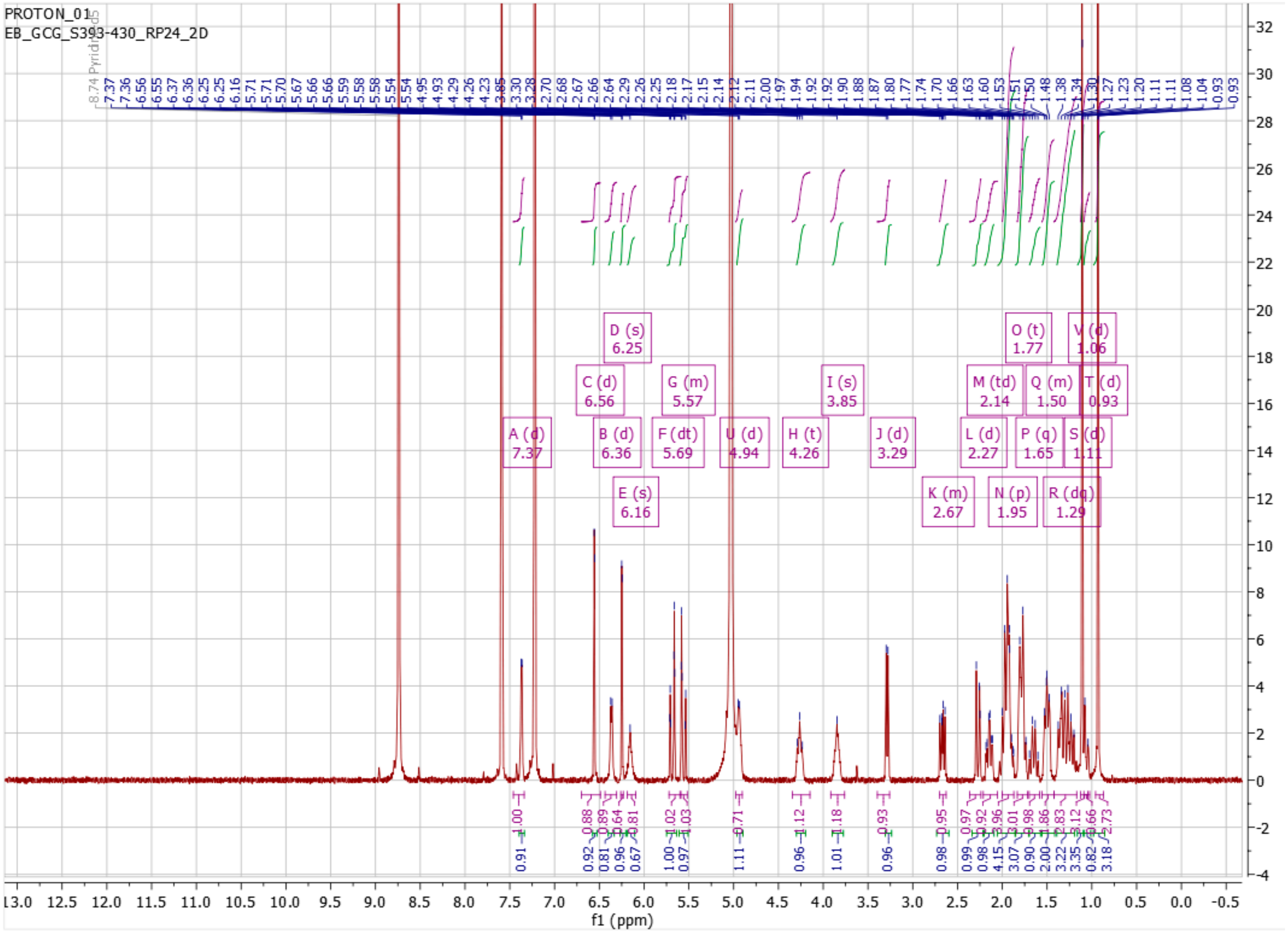

Figure S58. ^{1}H NMR Spectrum of 7(β)-hydroxy-3-epi-gitoxigenin (**compound 6**)

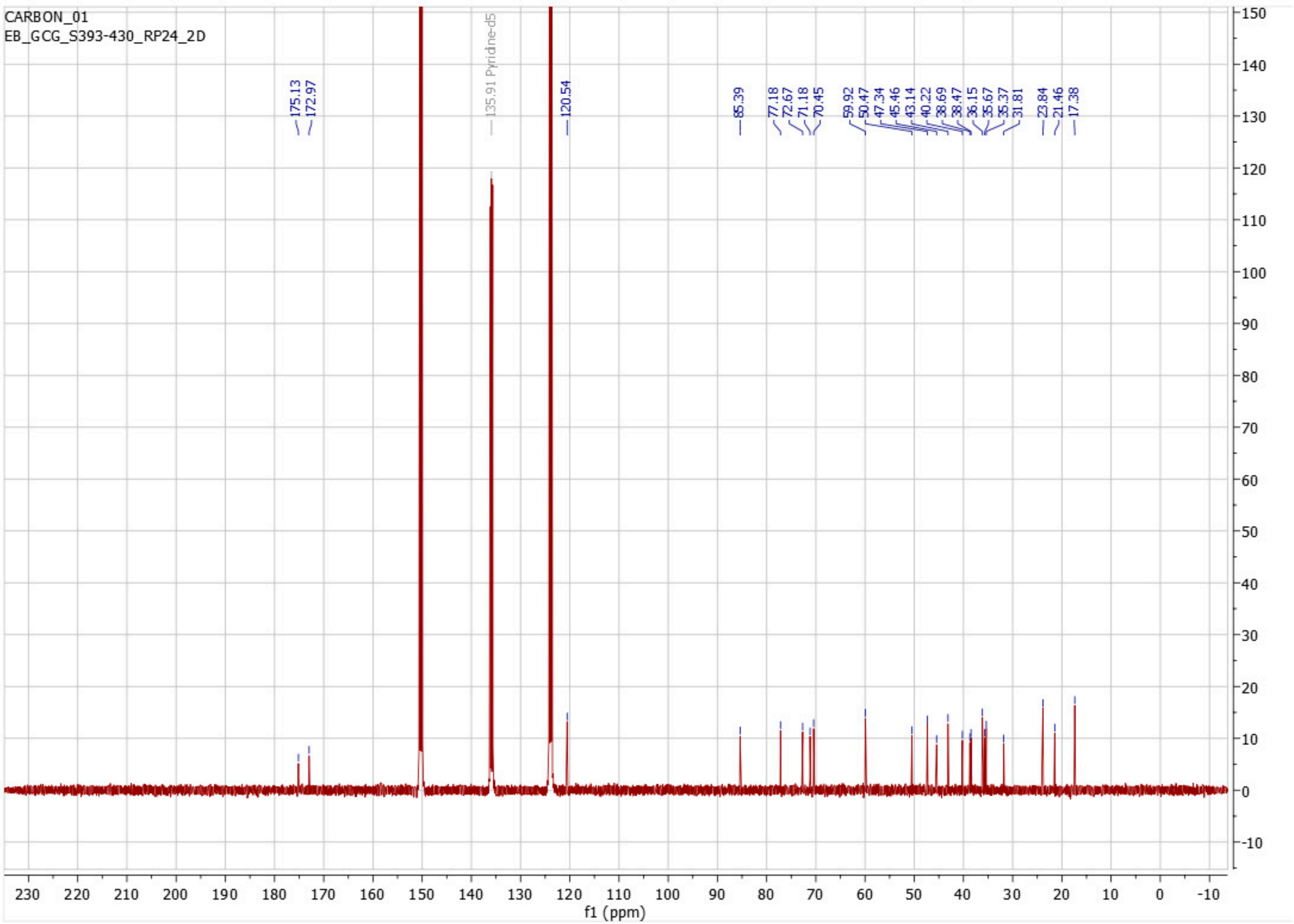

Figure S59. ^{13}C NMR Spectrum of 7(*β*)-hydroxy-3-epi-gitoxigenin (**compound 6**)

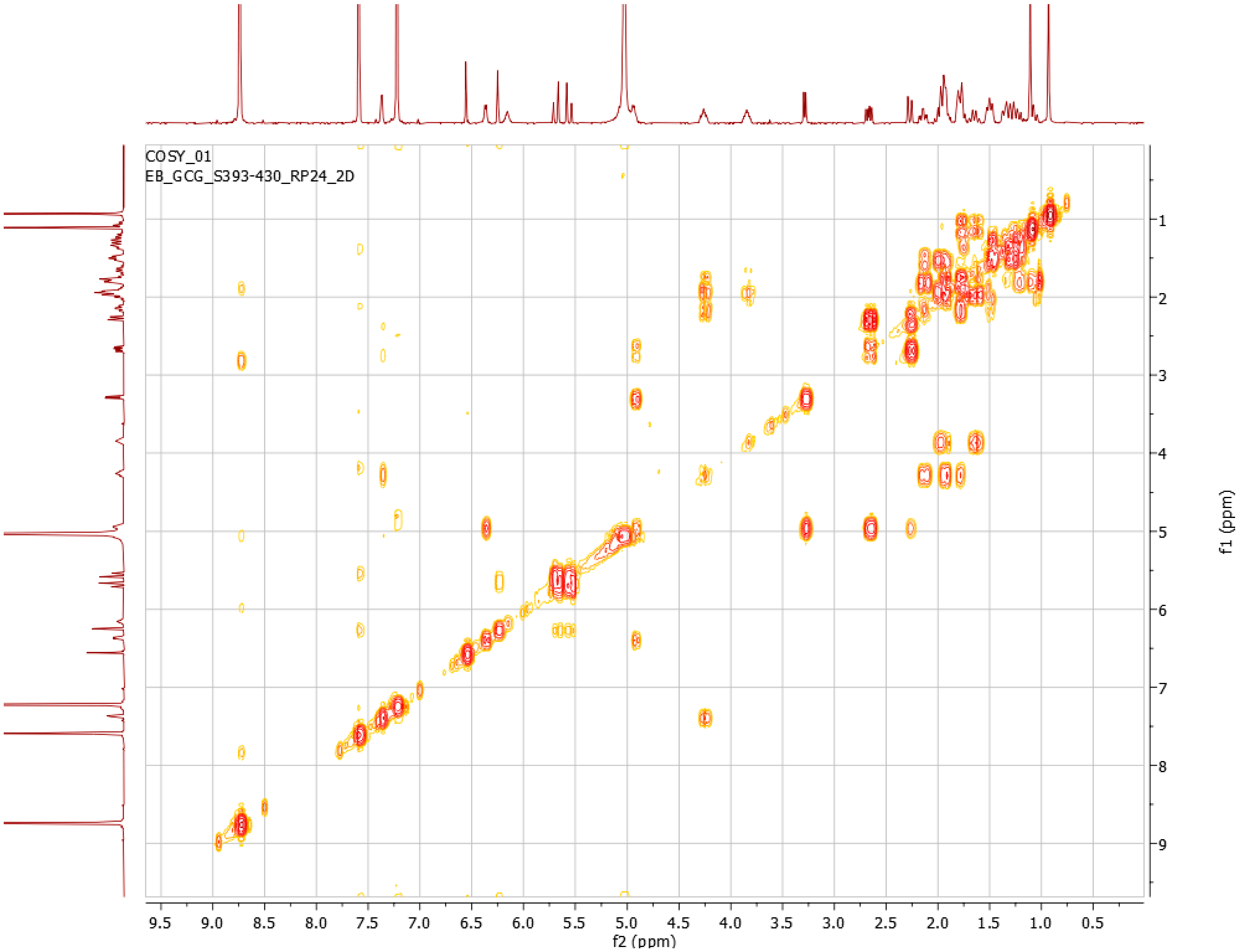

Figure S60. COSY Spectrum of 7(β)-hydroxy-3-epi-gitoxigenin (**compound 6**)

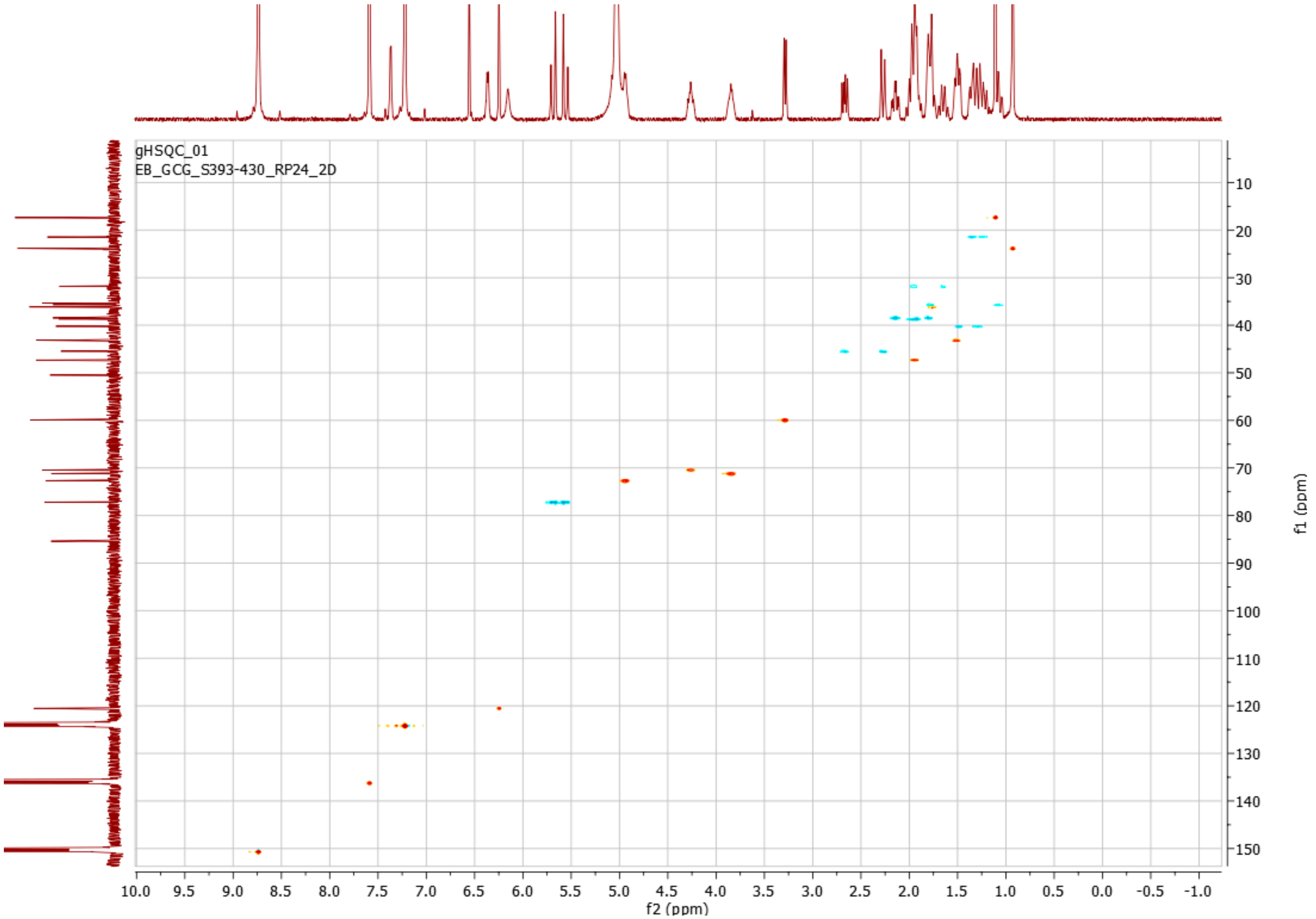

Figure S61. HSQC Spectrum of 7(β)-hydroxy-3-epi-gitoxigenin (**compound 6**)

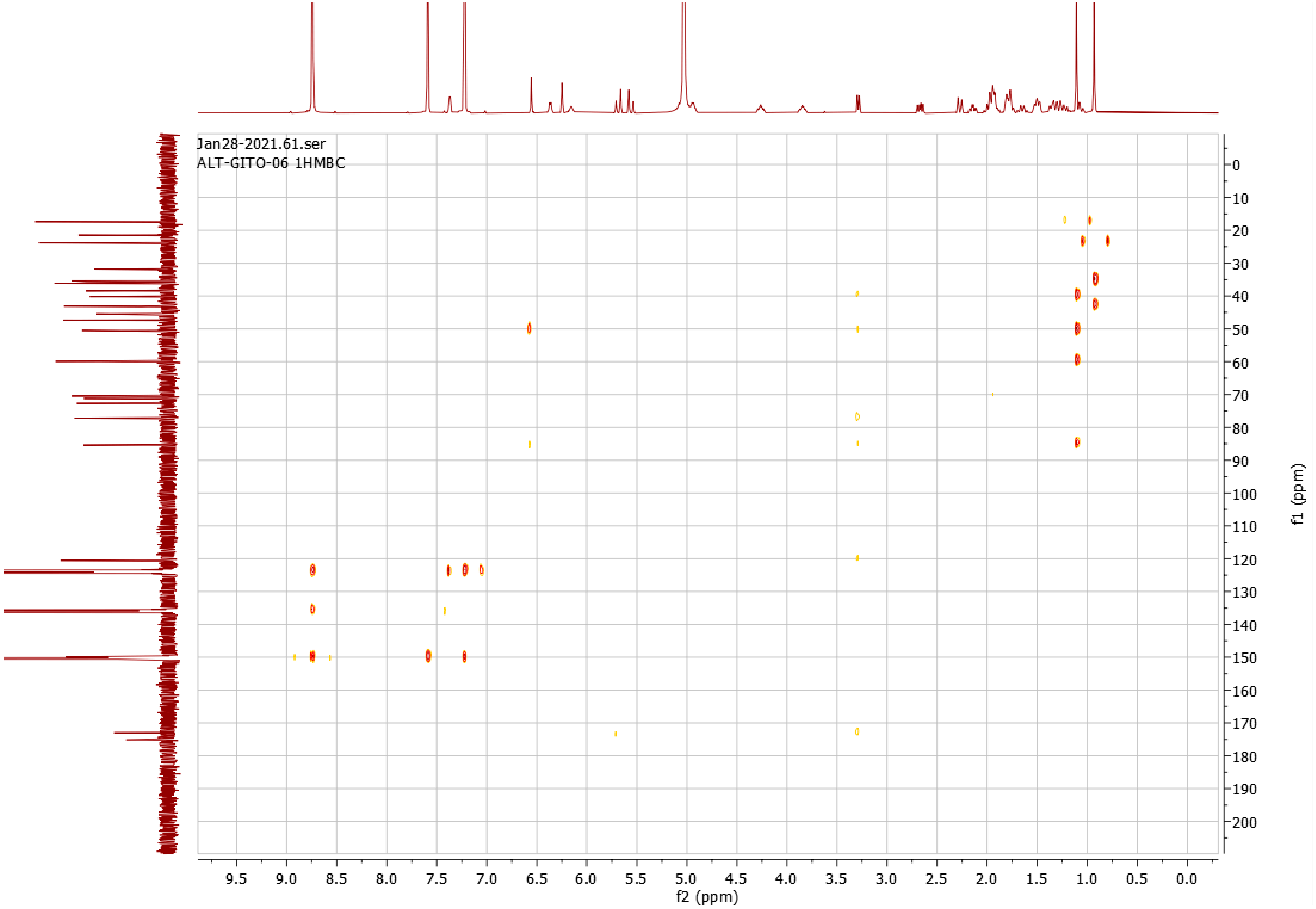

Figure S62. HMBC Spectrum of 7(β)-hydroxy-3-epi-gitoxigenin (**compound 6**)

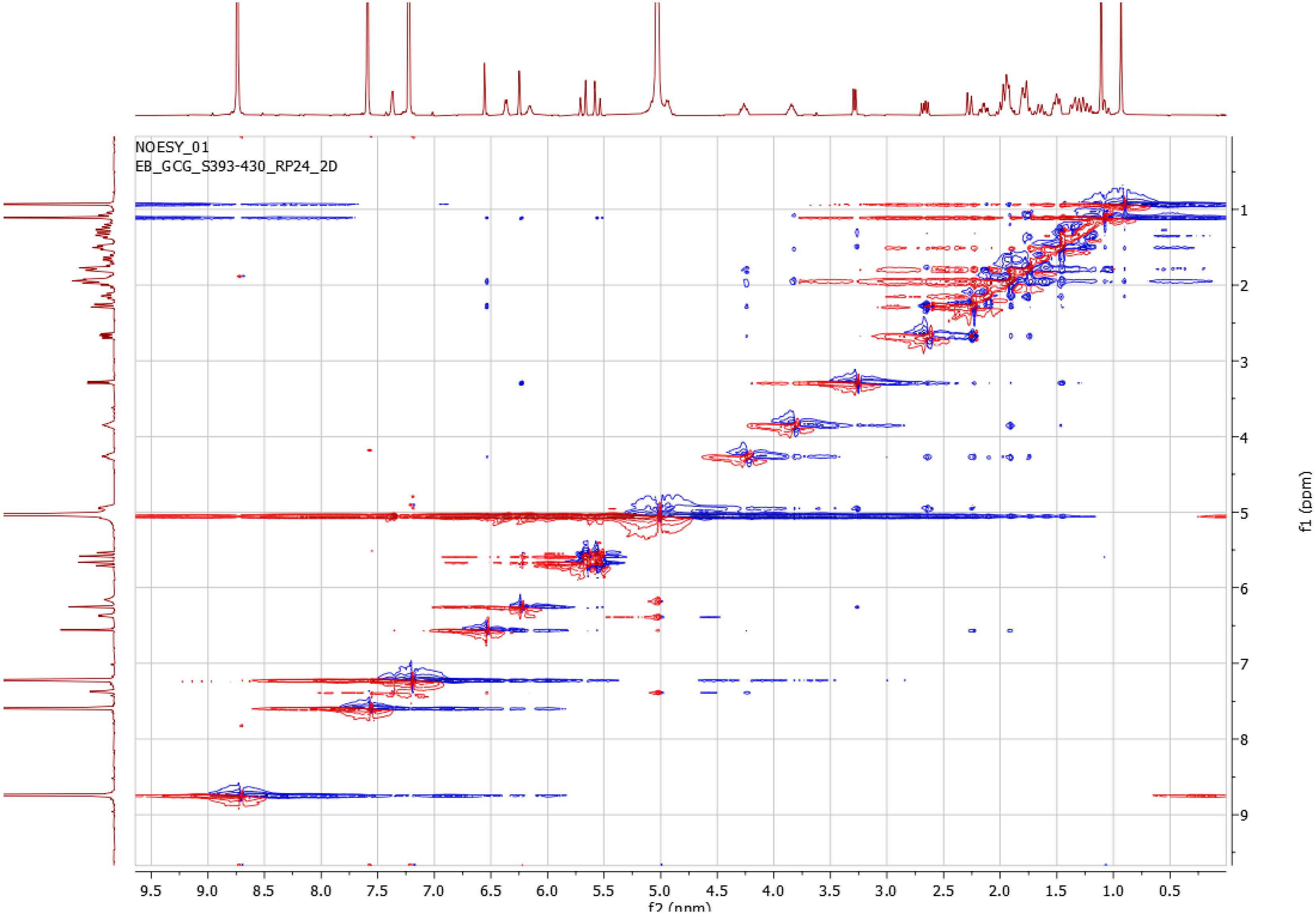

Figure S63. NOESY Spectrum of 7(β)-hydroxy-3-epi-gitoxigenin (**compound 6**)

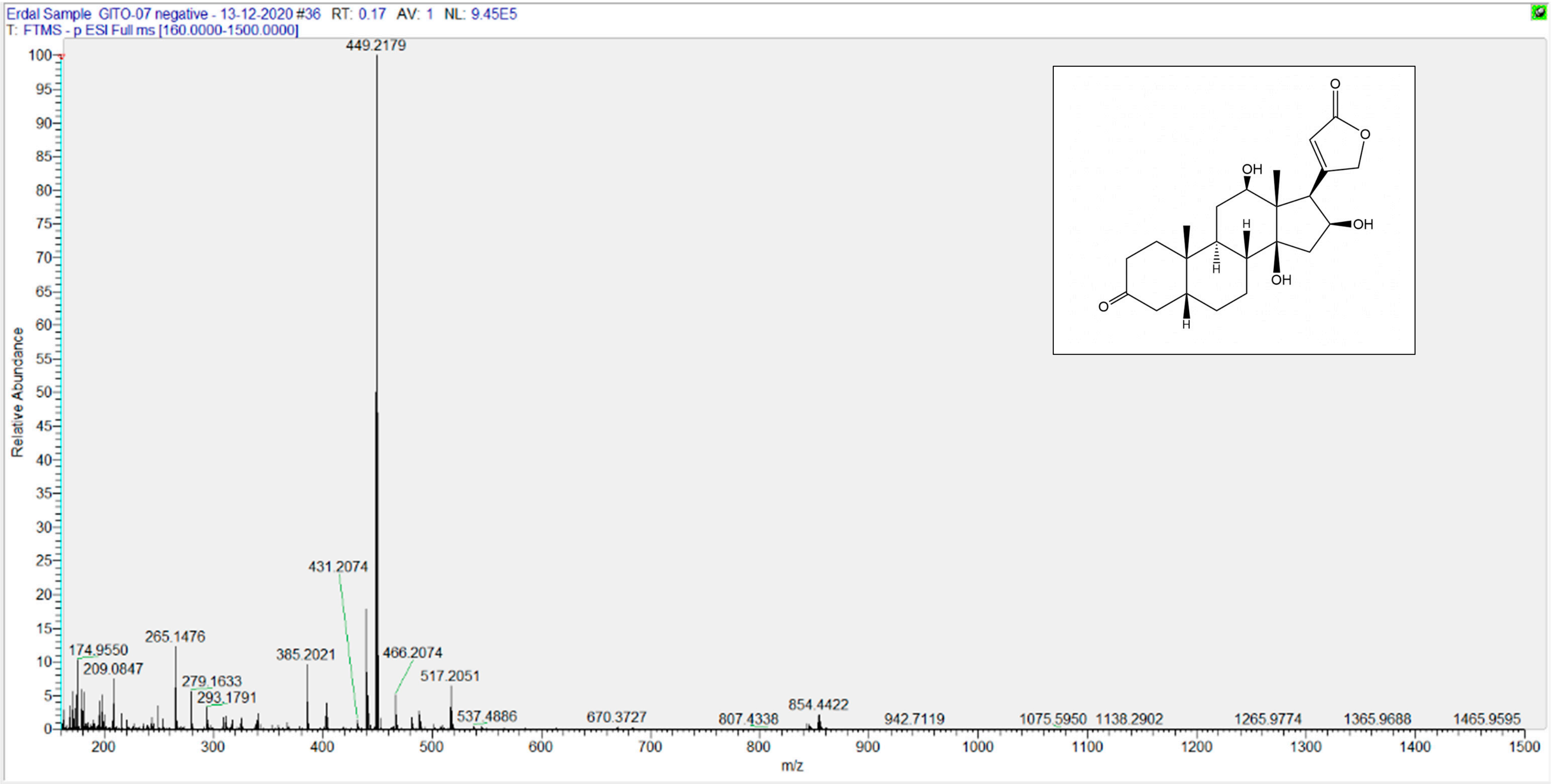

Figure S64. Negative-ion mode HR-ESI-MS spectrum of 3-oxo-diginatigenin (**compound 7**)

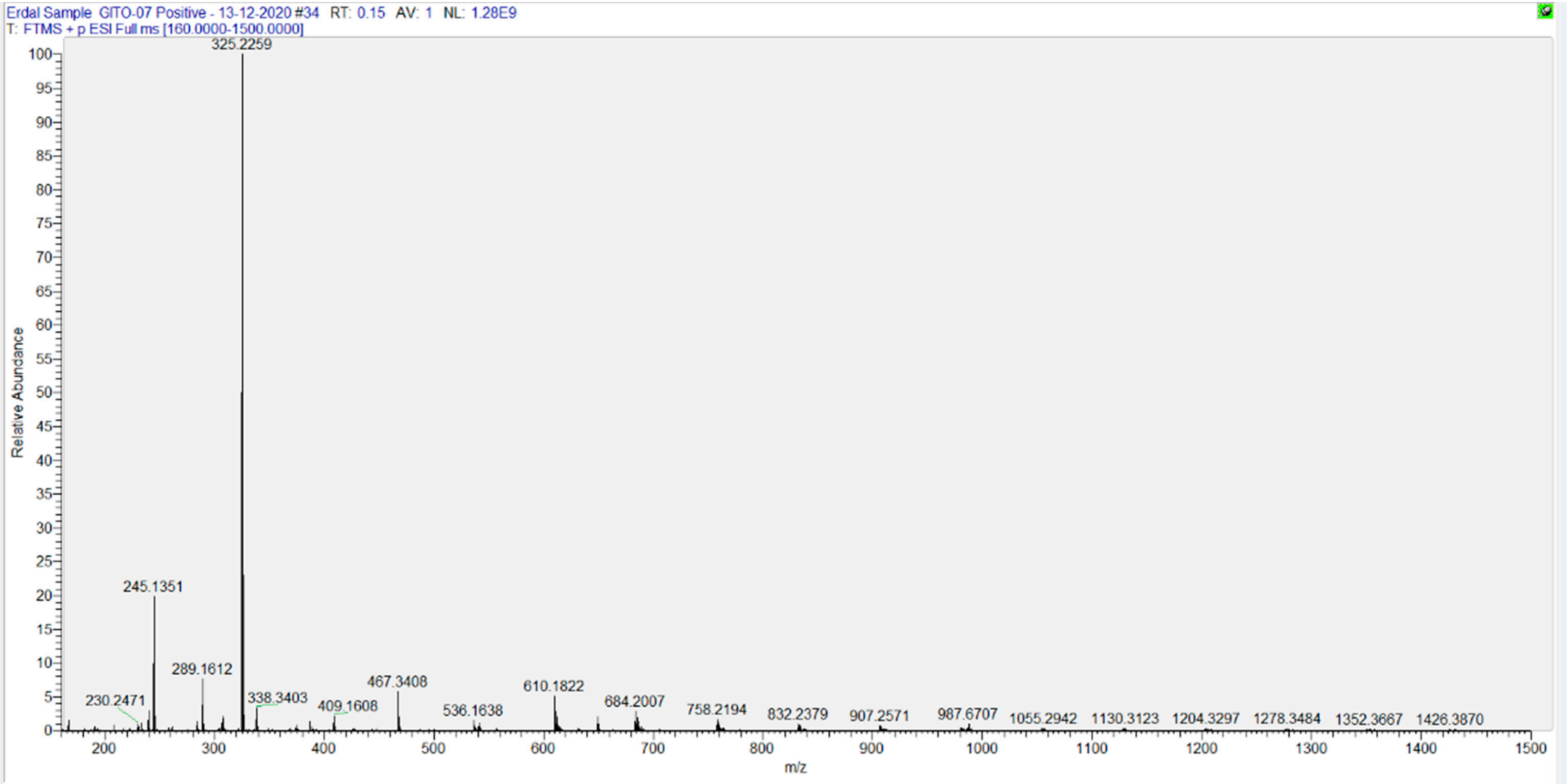

Figure S65. Positive-ion mode HR-ESI-MS spectrum of 3-oxo-diginatigenin (**compound 7**)

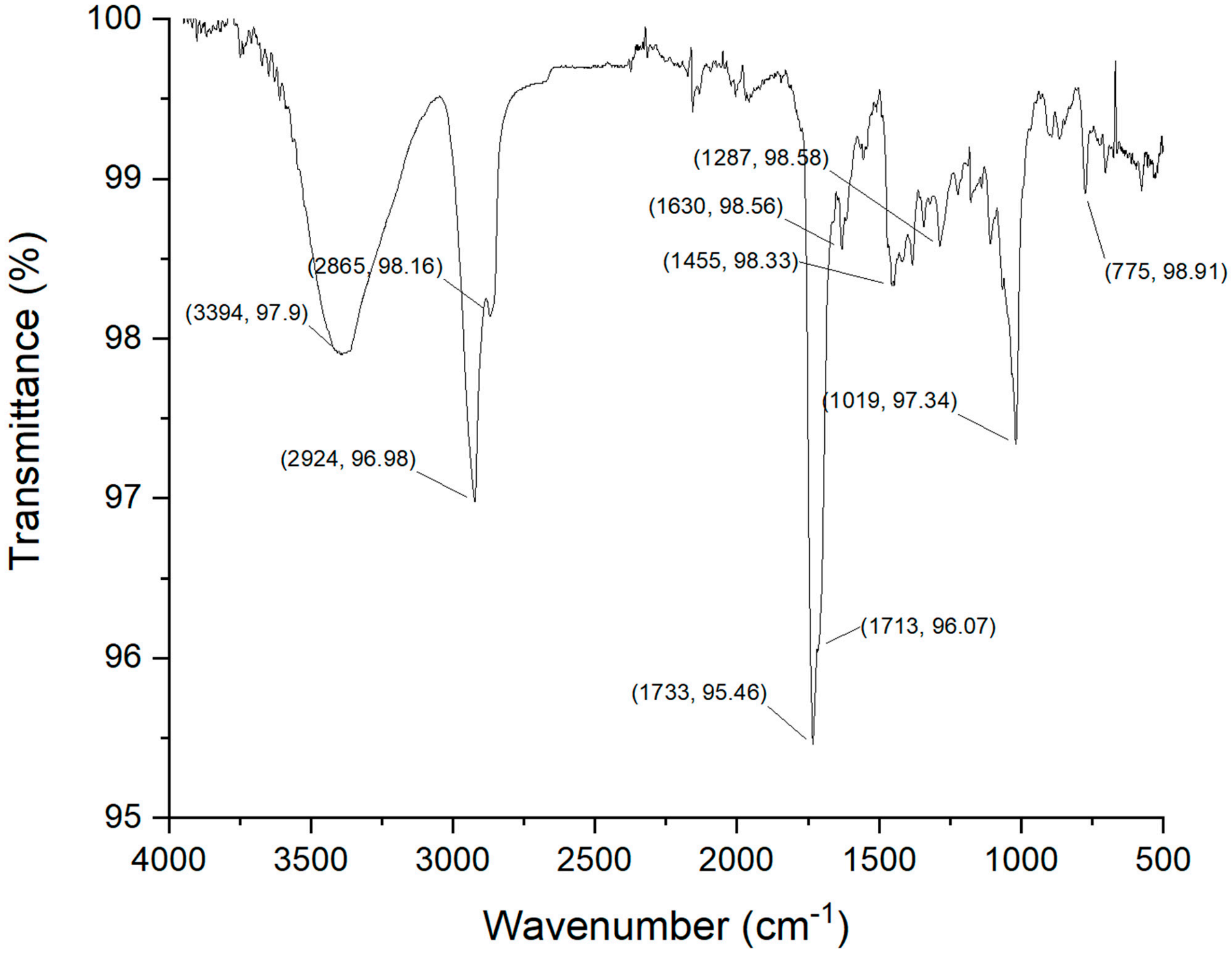

Figure S66. FT-IR Spectrum of 3-oxo-diginatigenin (**compound 7**)

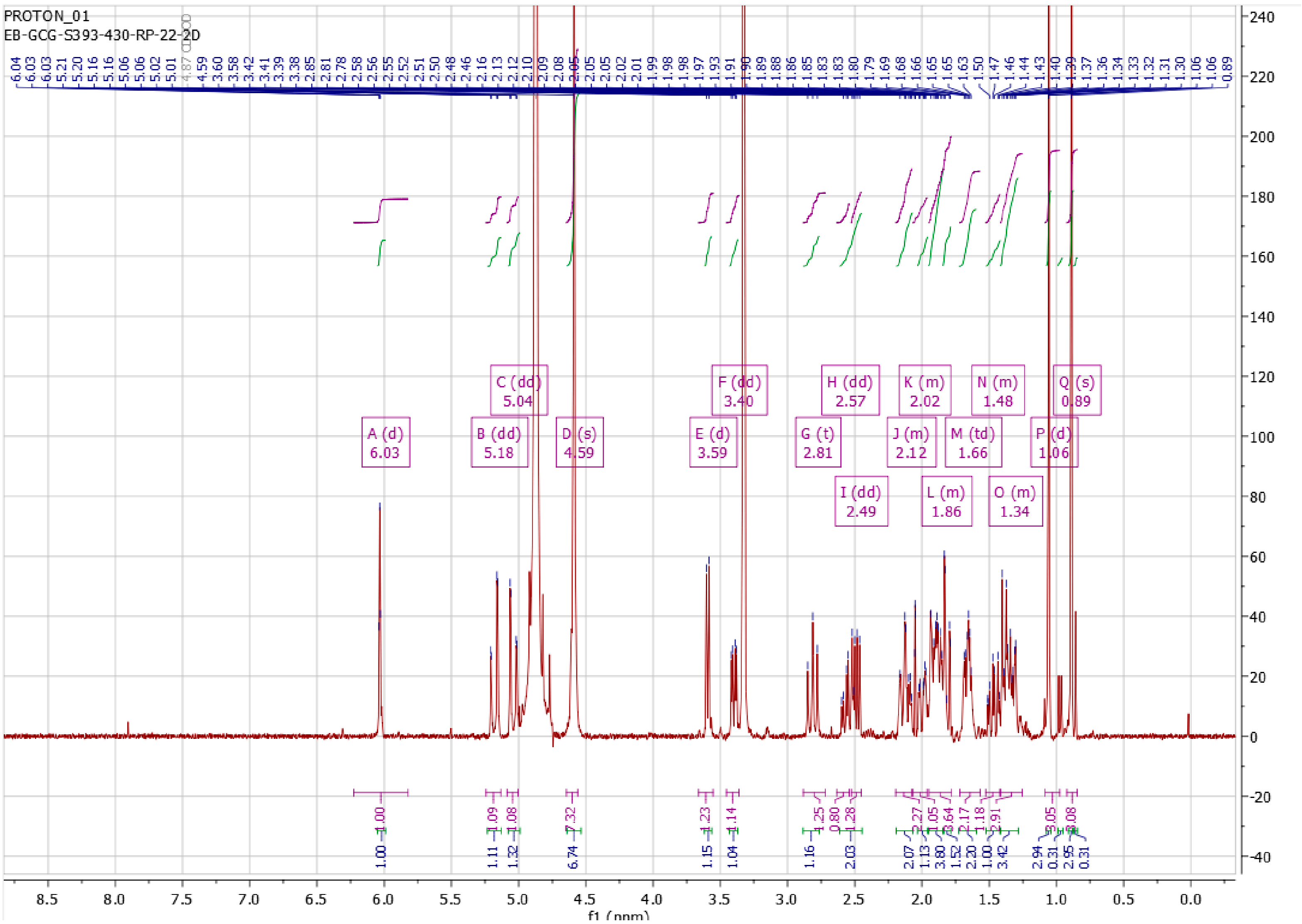

Figure S67. ^{1}H NMR Spectrum of 3-oxo-diginatigenin (**compound 7**)

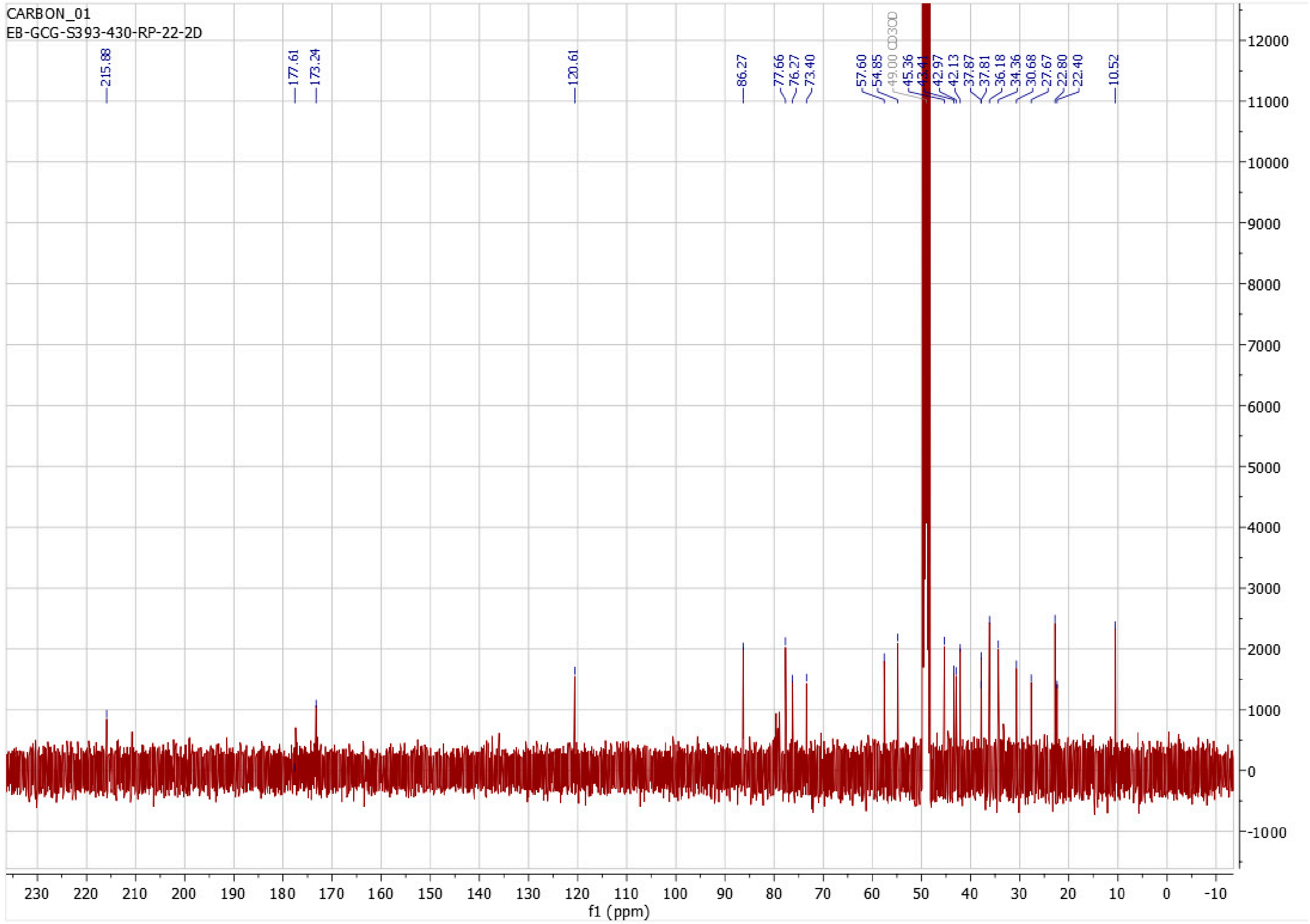

Figure S68. ^{13}C NMR Spectrum of 3-oxo-diginatigenin (**compound 7**)

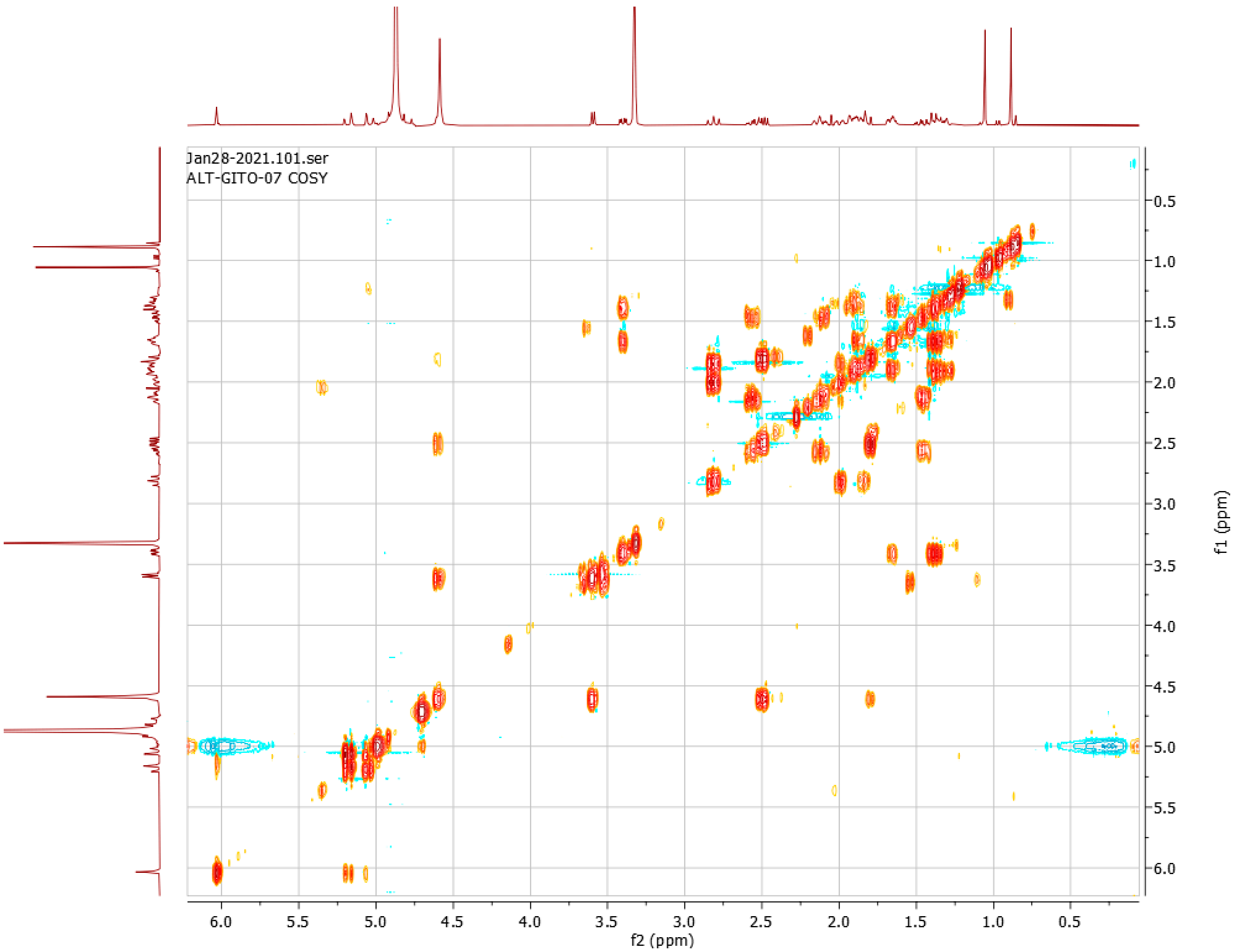

Figure S69. COSY Spectrum of 3-oxo-diginatigenin (**compound 7**)

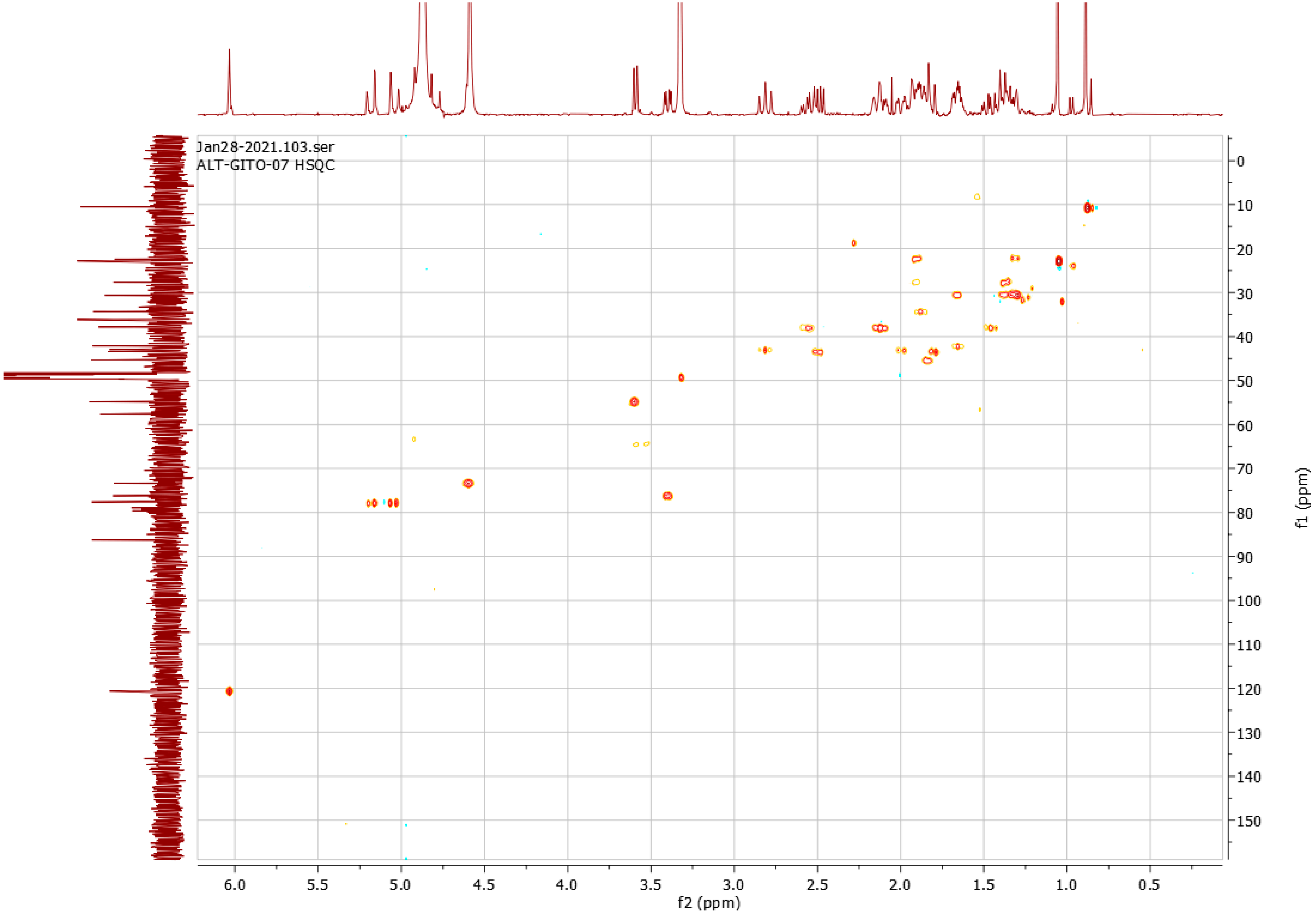

Figure S70. HSQC Spectrum of 3-oxo-diginatigenin (**compound 7**)

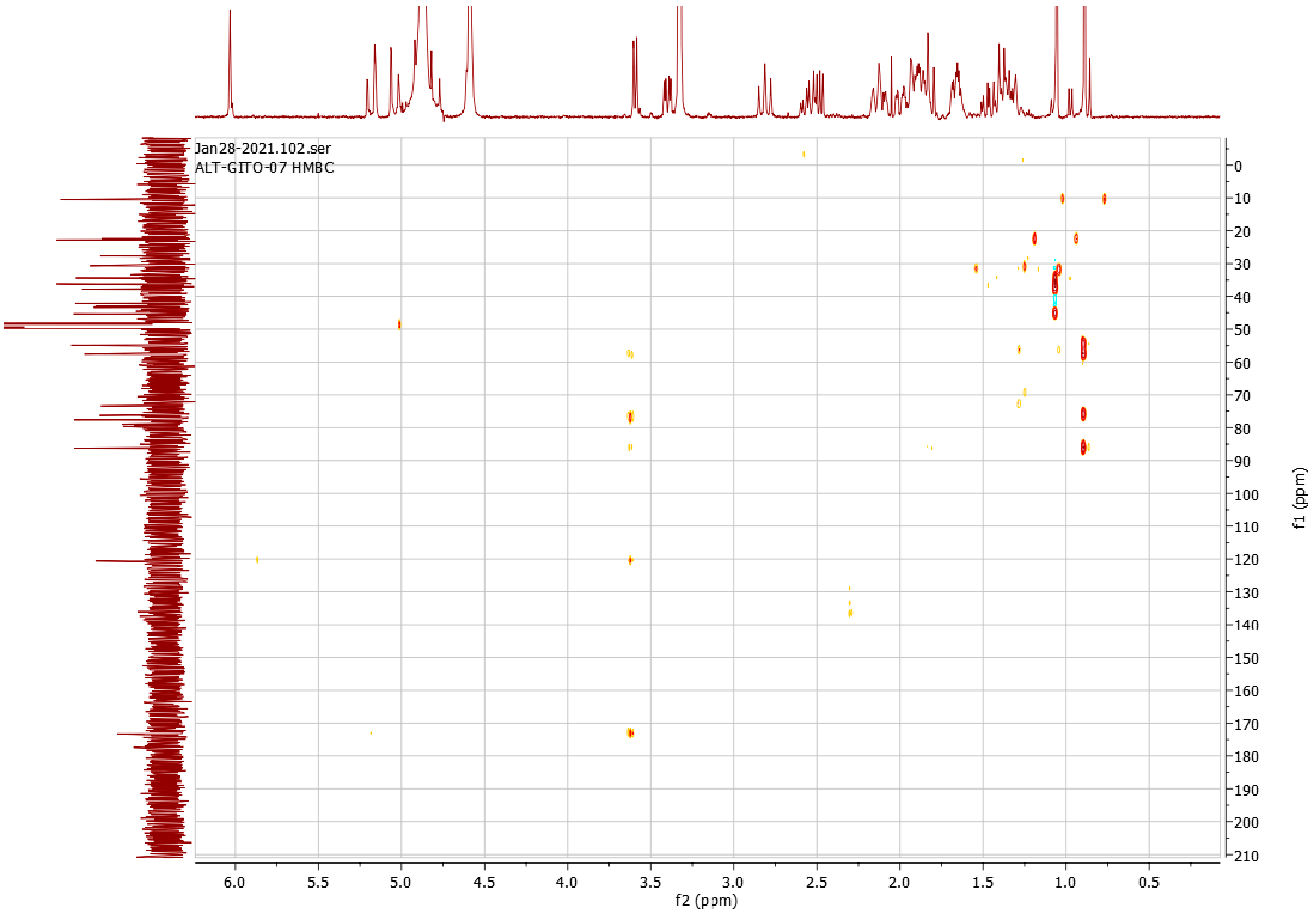

Figure S71. HMBC Spectrum of 3-oxo-diginatigenin (**compound 7**)

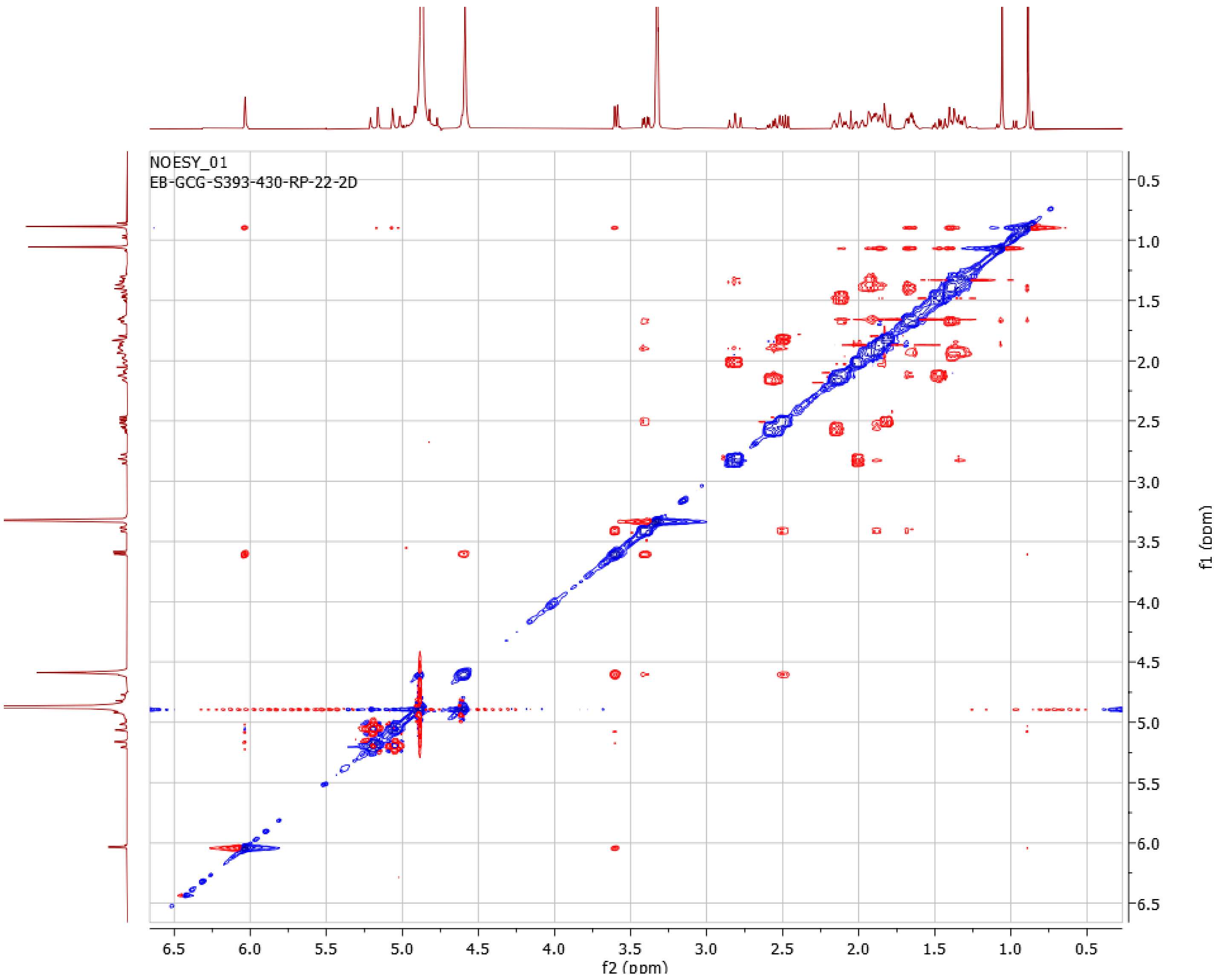

Figure S72. NOESY Spectrum of 3-oxo-diginatigenin (**compound 7**)

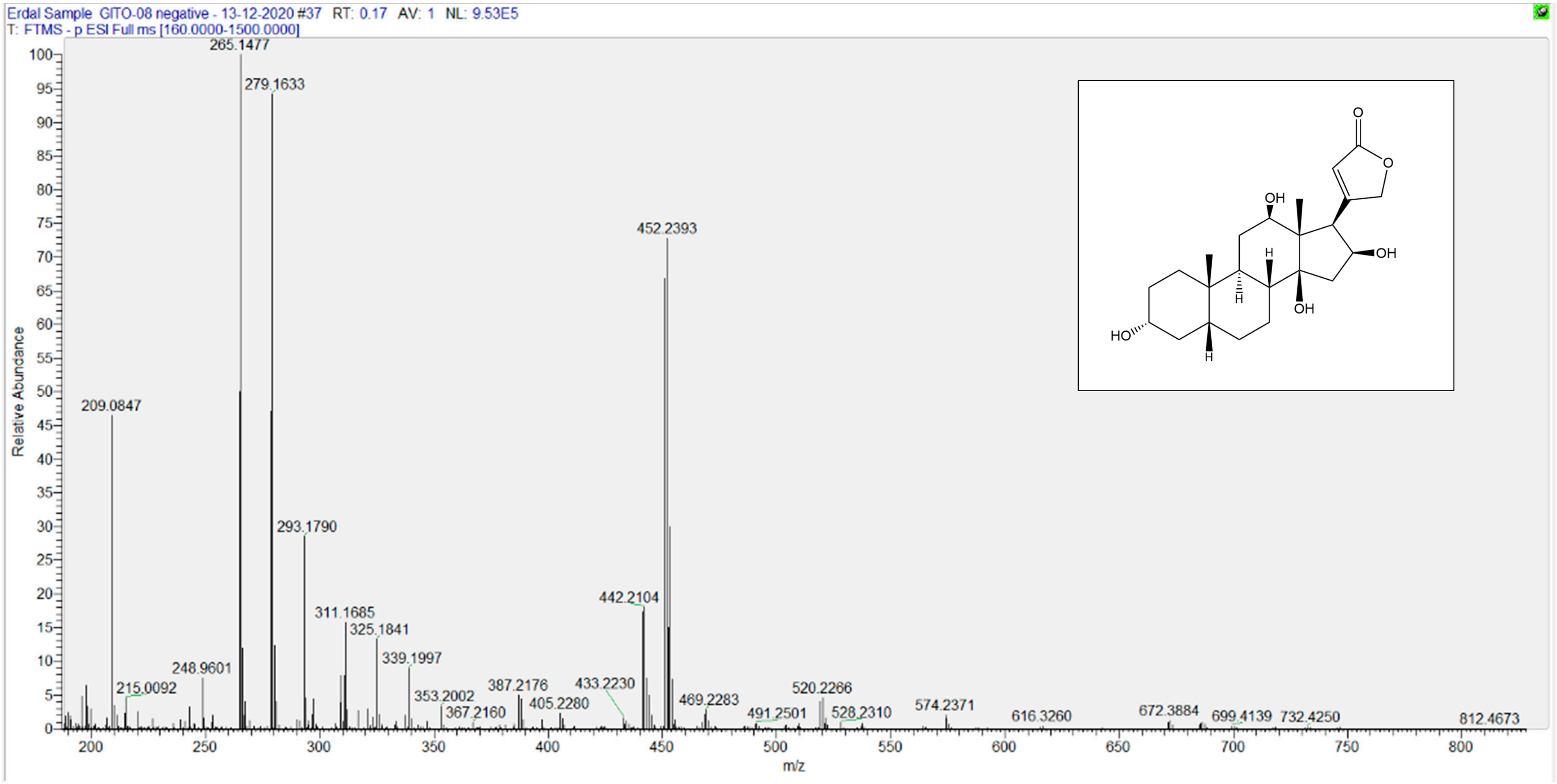

Figure S73. Negative-ion mode HR-ESI-MS spectrum of 3-epi-diginatigenin (**compound 8**)

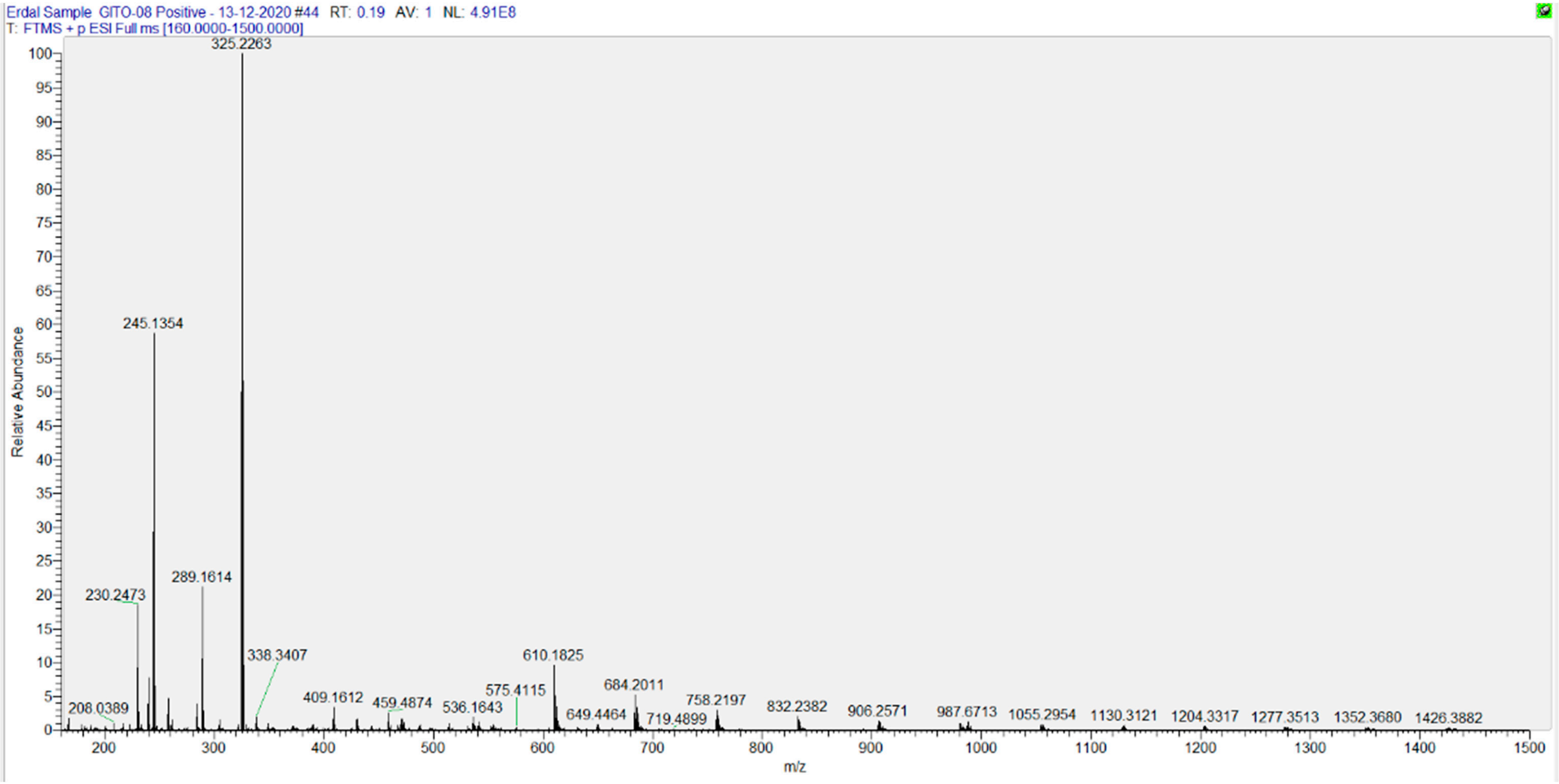

Figure S74. Positive-ion mode HR-ESI-MS spectrum of 3-epi-diginatigenin (**compound 8**)

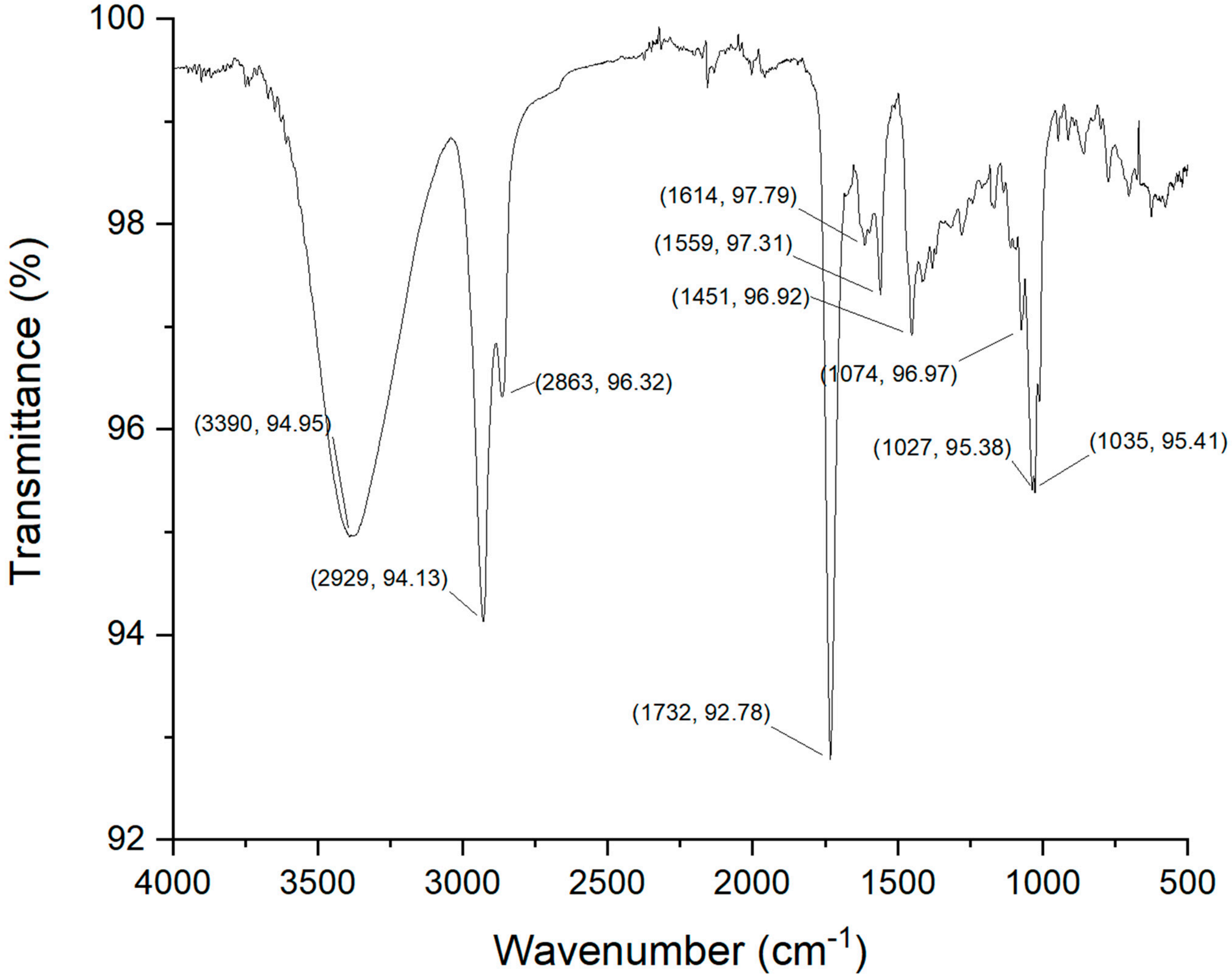

Figure S75. FT-IR Spectrum of 3-epi-diginatigenin (**compound 8**)

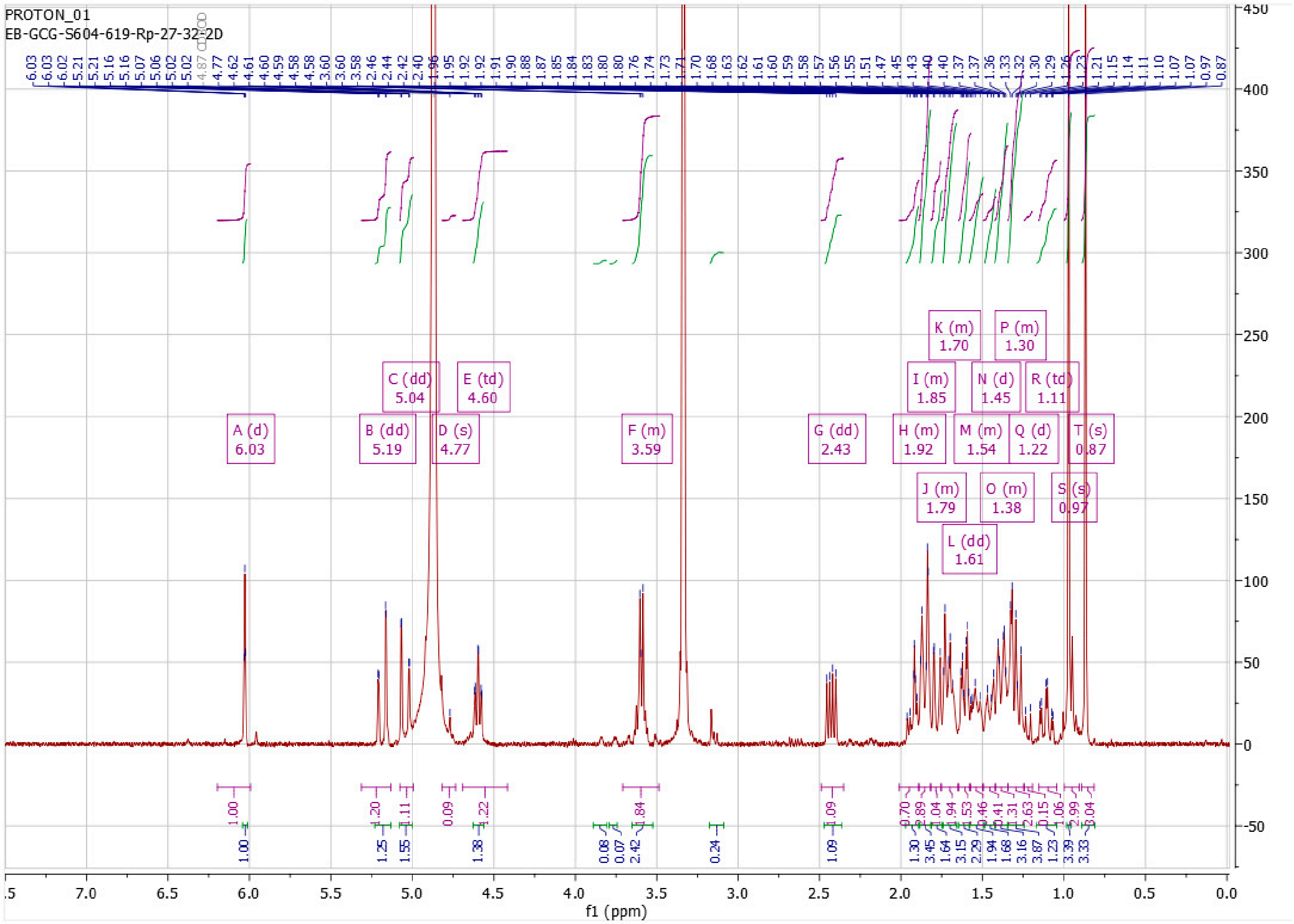

Figure S76. ^{1}H NMR Spectrum of 3-epi-diginatigenin (**compound 8**)

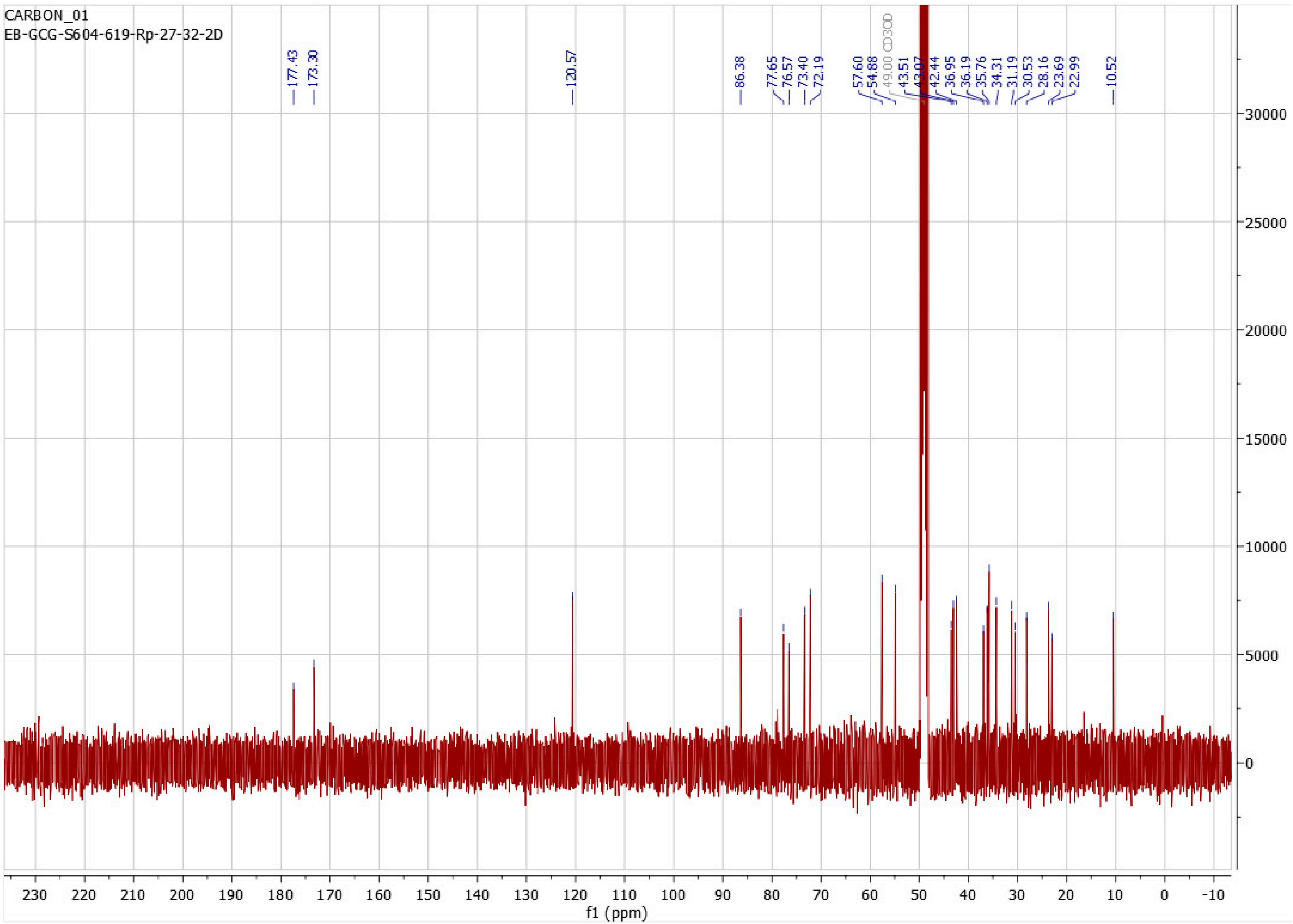

Figure S77. ^{13}C NMR Spectrum of 3-epi-diginatigenin (**compound 8**)

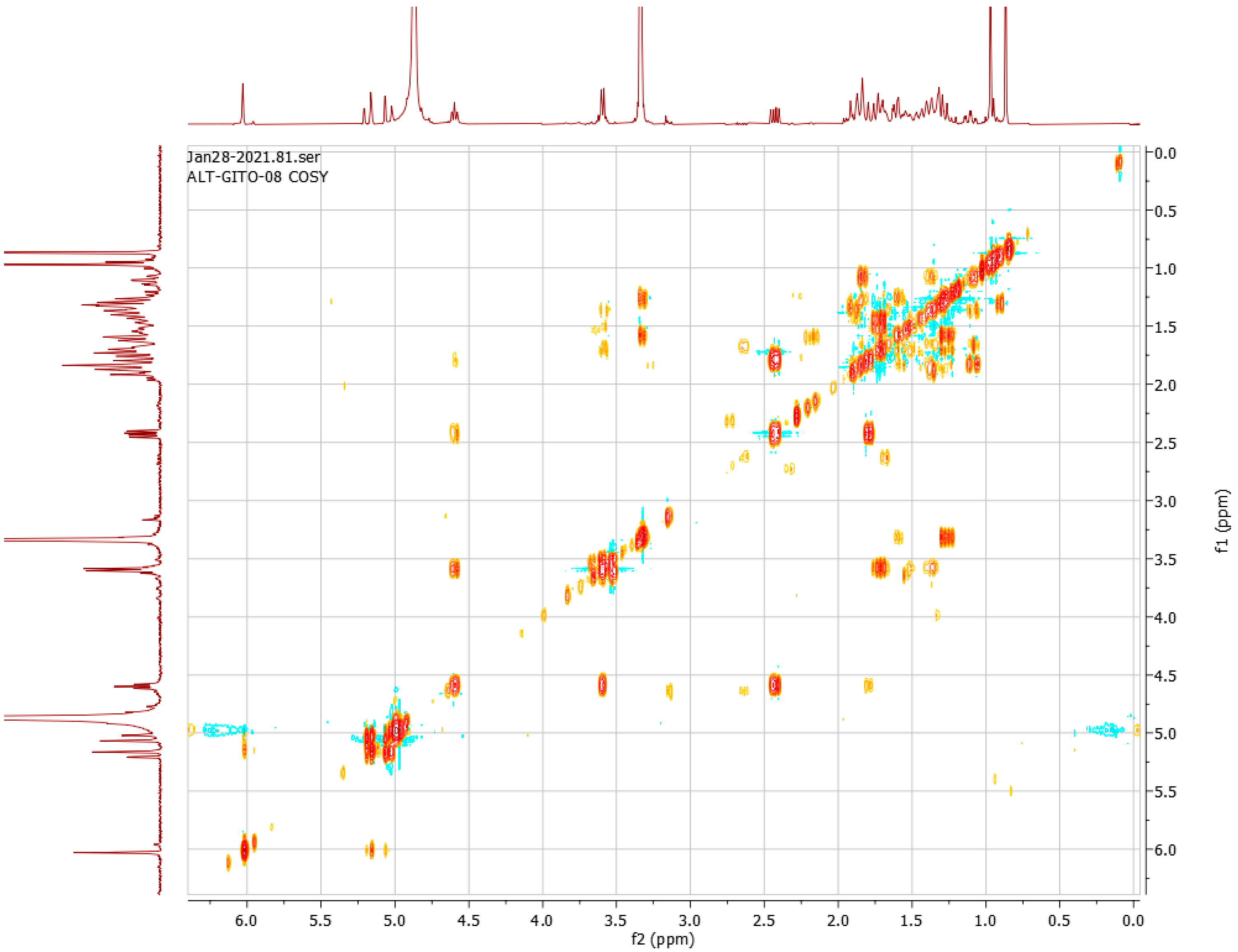

Figure S78. COSY Spectrum of 3-epi-diginatigenin (**compound 8**)

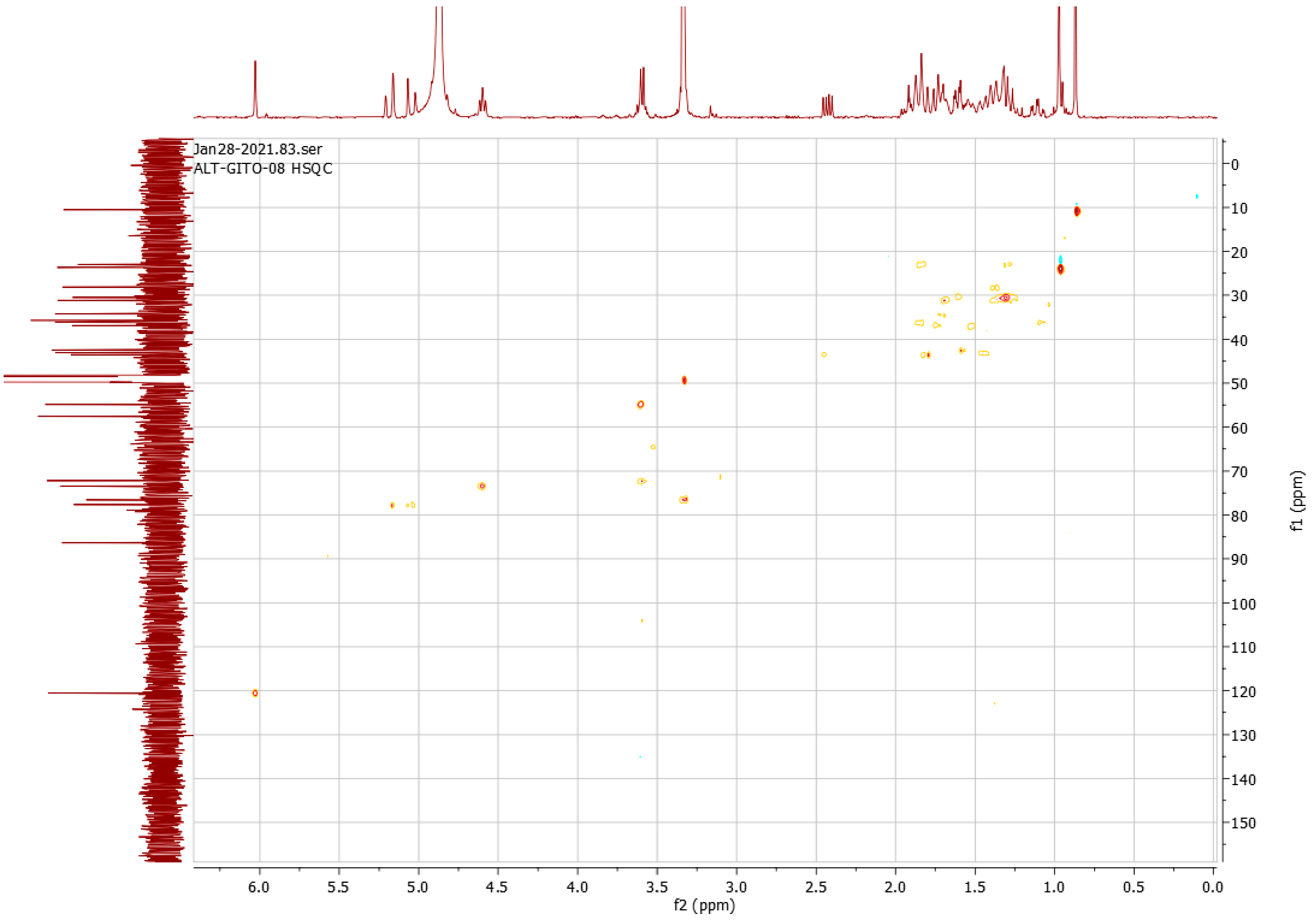

Figure S79. HSQC Spectrum of 3-epi-diginatigenin (**compound 8**)

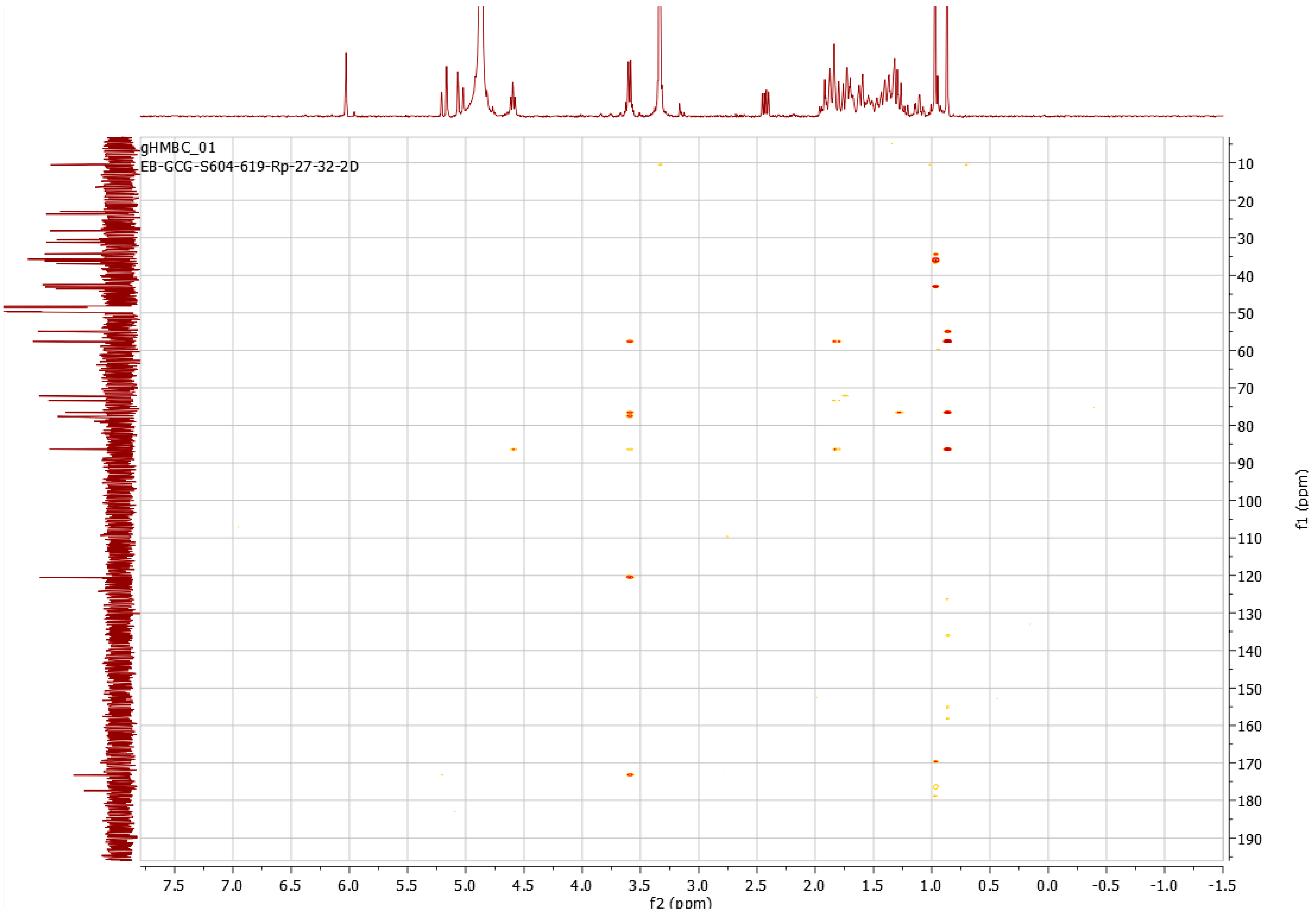

Figure S80. HMBC Spectrum of 3-epi-diginatigenin (**compound 8**)

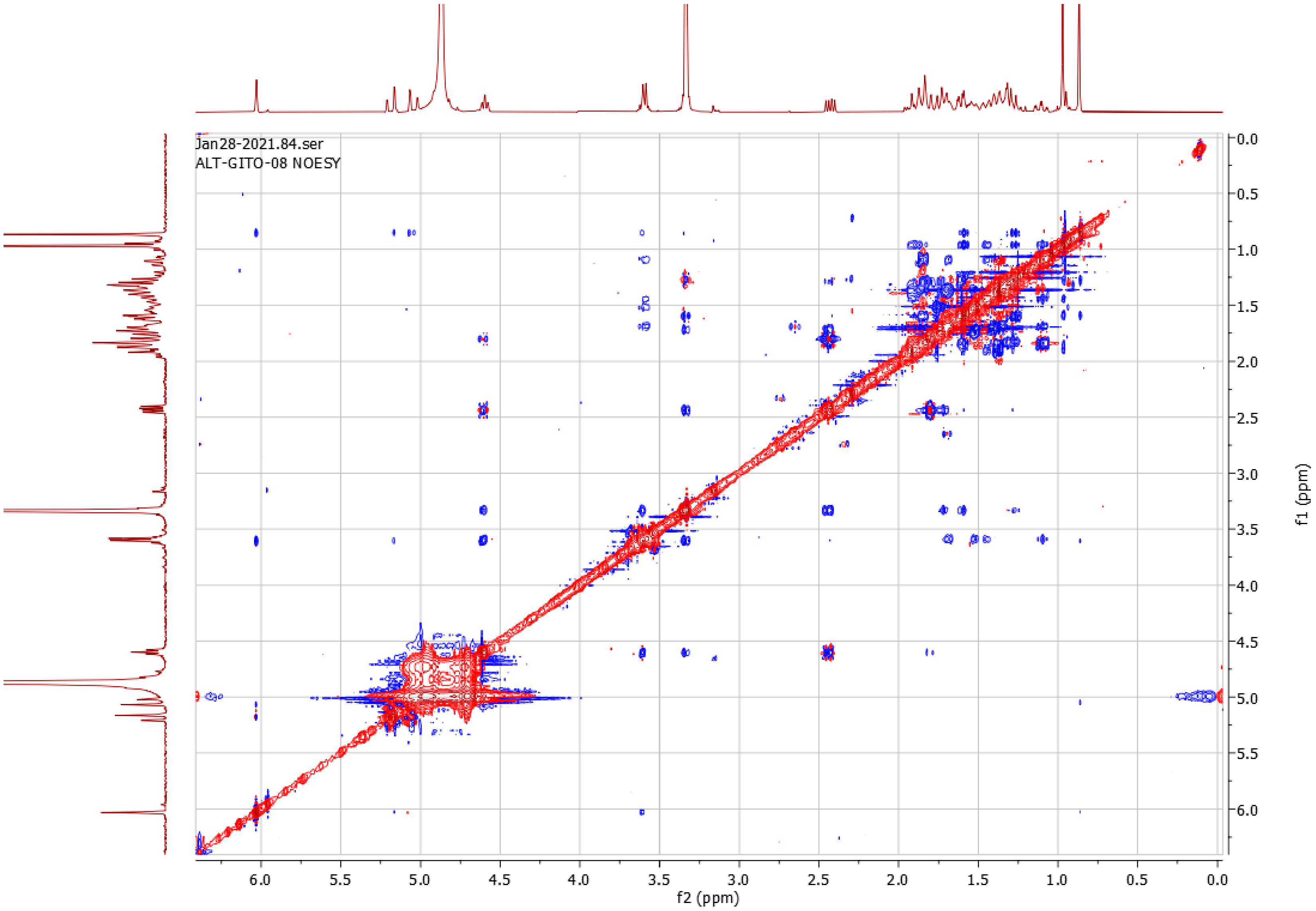

Figure S81. NOESY Spectrum of 3-epi-diginatigenin (**compound 8**)

Table S 1: HPLC Method for qualitative analysis of selected metabolites (**4**, **5** and **6**) and EtOAc extract of biotransformation broth.

**HPLC System**	Agilent 1200 Series HPLC System and DAD detector
**Stationary phase**	Kinetex C-18 column (150 x 4.6mm, 5µm particle size)
**Mobile Phase**	A:B (water: acetonitrile)- gradient*
**Flow rate**	1.0 mL/min
**Enjection volume**	20 µL
**Detection Wavelength**	210 nm

* After a 2 min hold at 15% B, elution was performed according to the following conditions: from 15% B to 70% B in 10 min; to 100% B in 3 min, hold to 100% B for 2 min and a re-equilibration period of 5min.

Samples with 1000 ppm concentration were prepared by using methanol. Prior to use, all samples were filtered through a 0.45 µm PTFE filter.

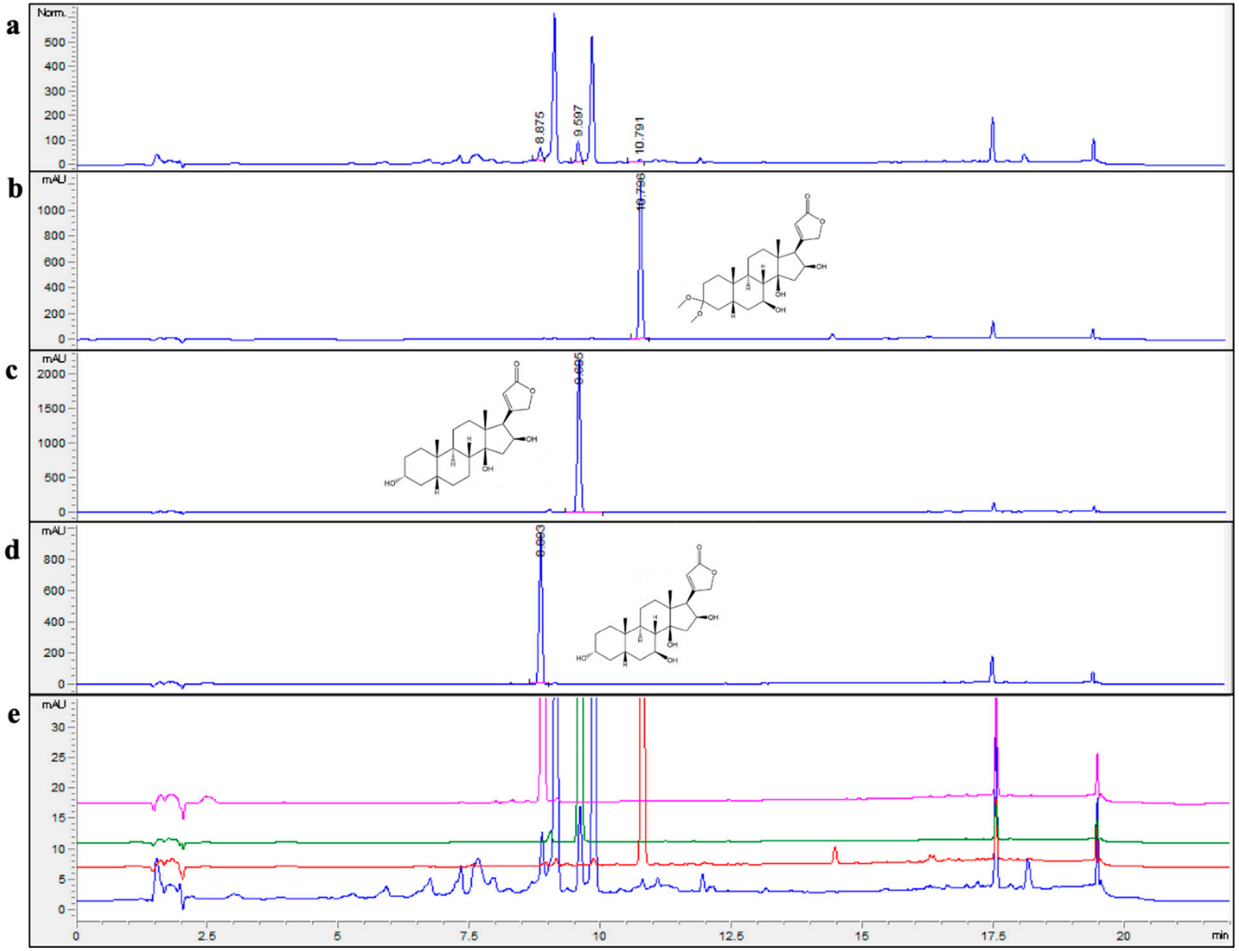

Chromatogram S1: HPLC chromatograms of **a)** EtOAc extract, **b) Compound 4** (retention time: 10.8 min), **c) Compound 5** (retention time: 9.6 min), **d) Compound 6** (retention time: 8.9 min) and **e)** comparison of the EtOAc extract (blue line) with **Compound 4** (red line), **Compound 5** (green line), **Compound 6** (pink line).

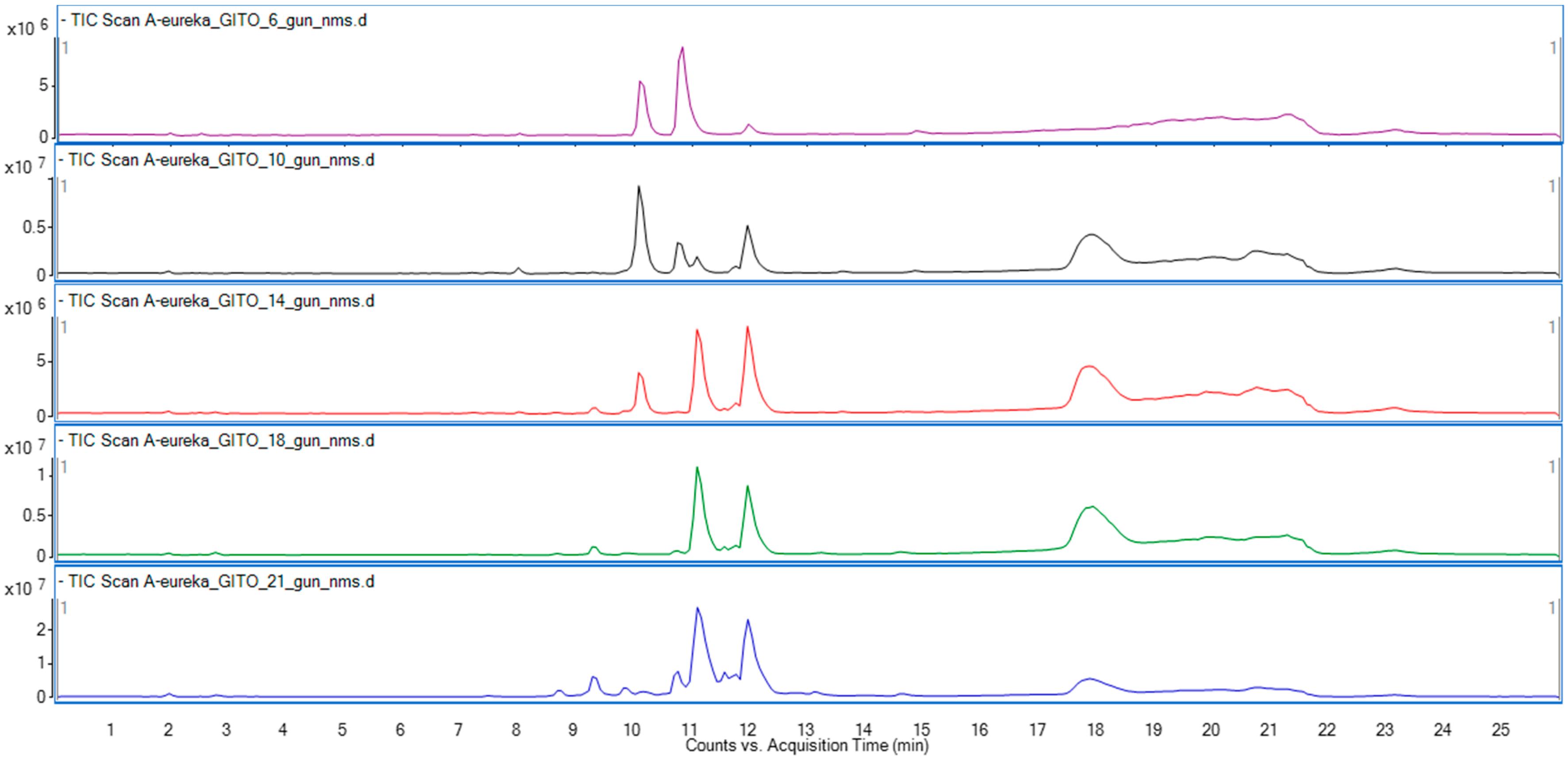

Chromatogram S2: LC-Q-ToF total ion chromatograms of the EtOAc extracts showing different biotransformation time points (Day 6, 10, 14, 18 and 21) (in negative mode).

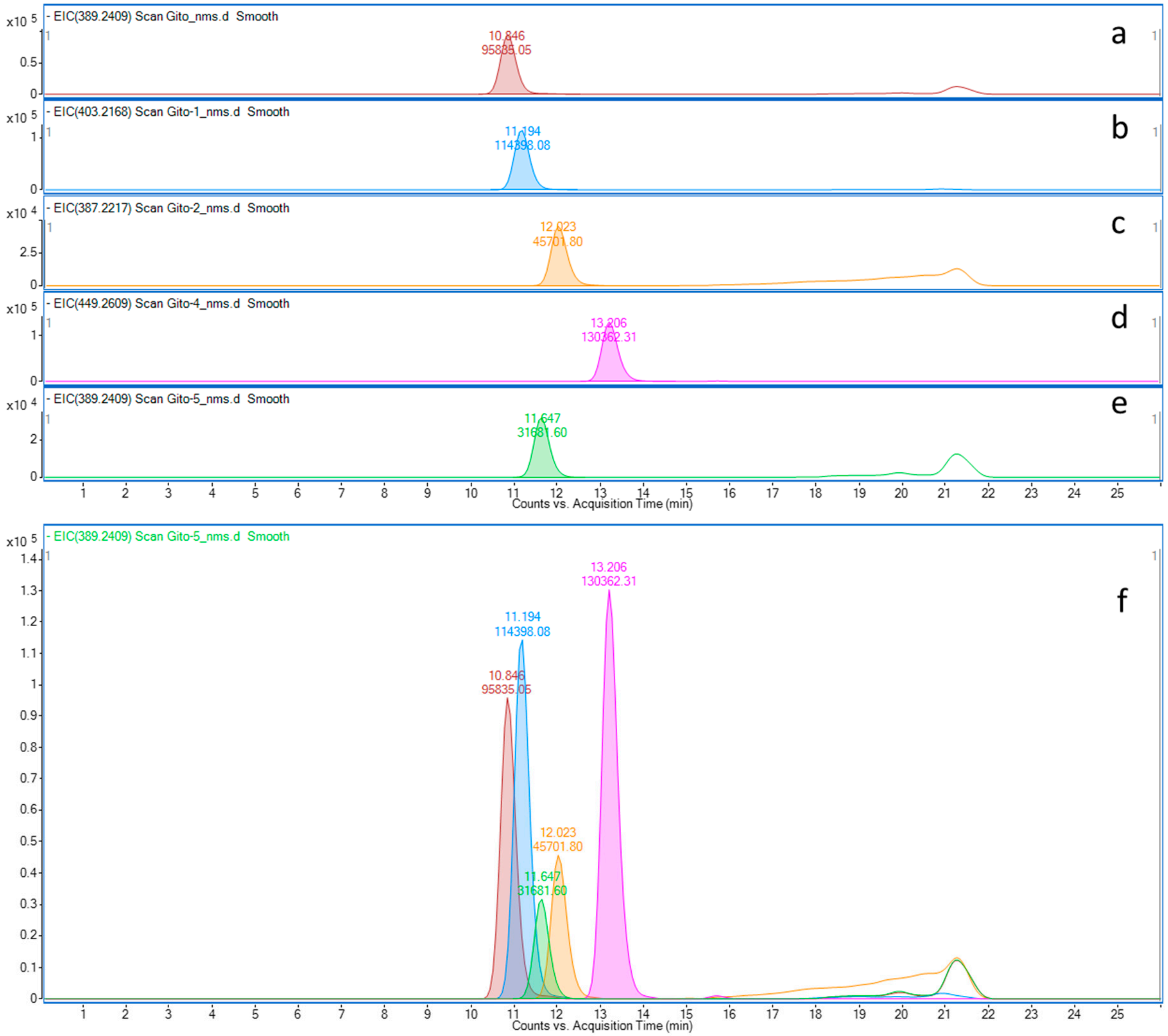

Chromatogram S3 : LC-Q-ToF extracted ion chromatograms of the selected compounds (Gitoxigenin, **1**, **2**, **4** and **5**) (in negative mode); **a)** Gitoxigenin (retention time: 10.85 min), **b) Compound 1** (retention time: 11.19 min), **c) Compound 2** (retention time: 12.02 min), **d) Compound 4** (retention time: 13.21 min), **e) Compound 5** (retention time: 11.65 min) and **f)** overlapped chromatograms

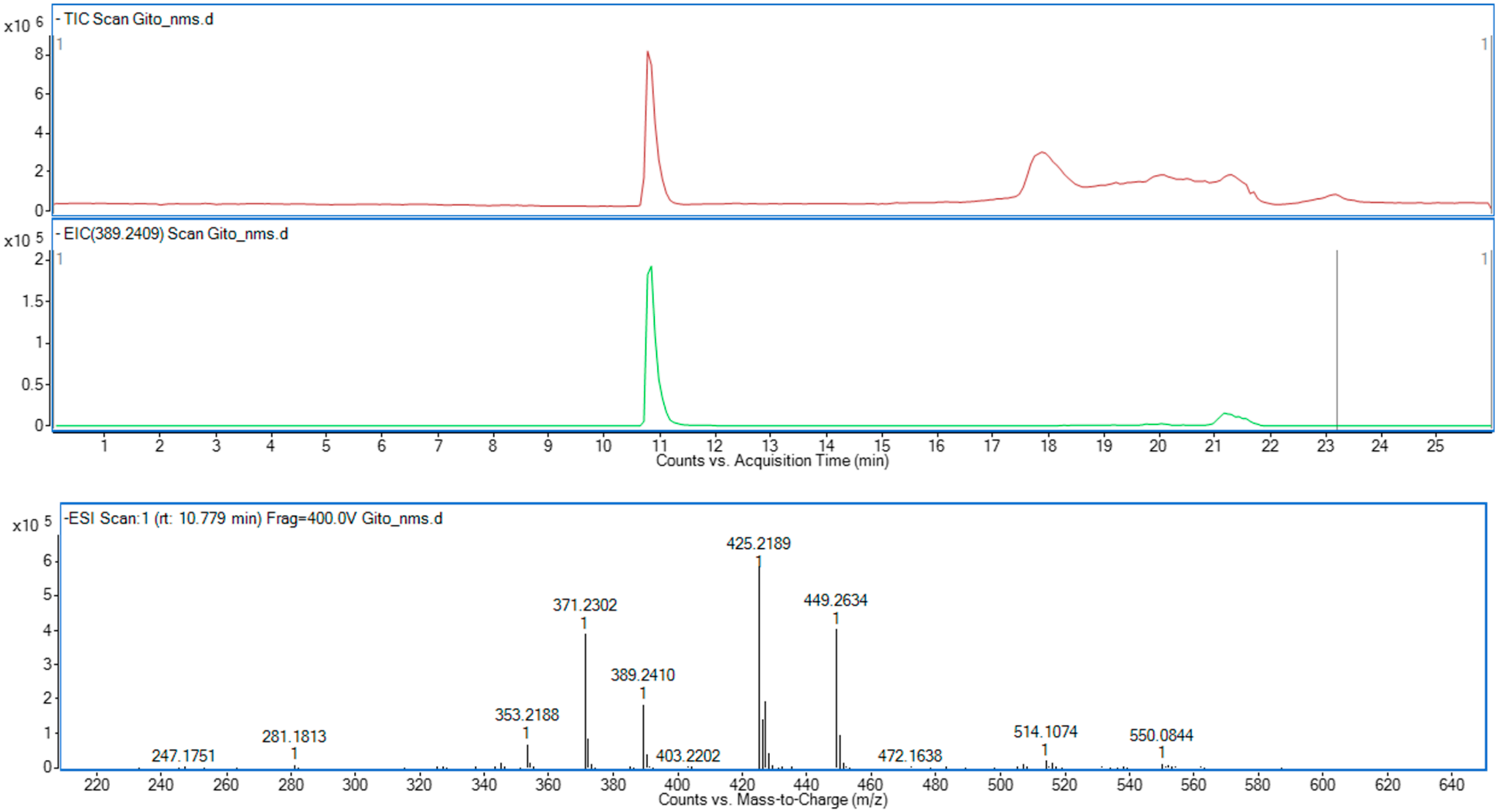

Chromatogram S4: LC-Q-ToF total and extracted ion chromatograms of gitoxigenin together with its mass spectrum (in negative mode).

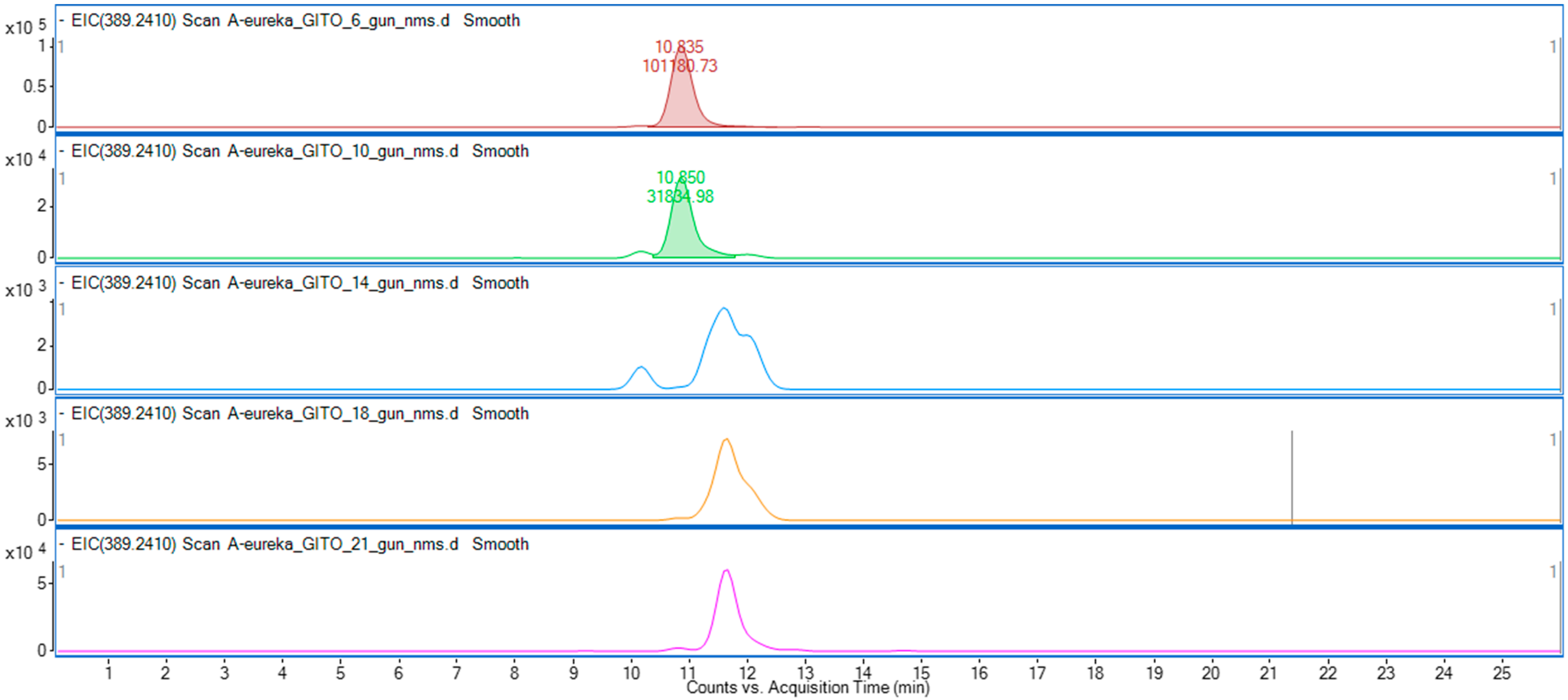

Chromatogram S5: LC-Q-ToF extracted ion chromatograms of gitoxigenin for different biotransformation time points (Day 6, 10, 14, 18 and 21), displaying that gitoxigenin was consumed before Day 14 (in negative mode).

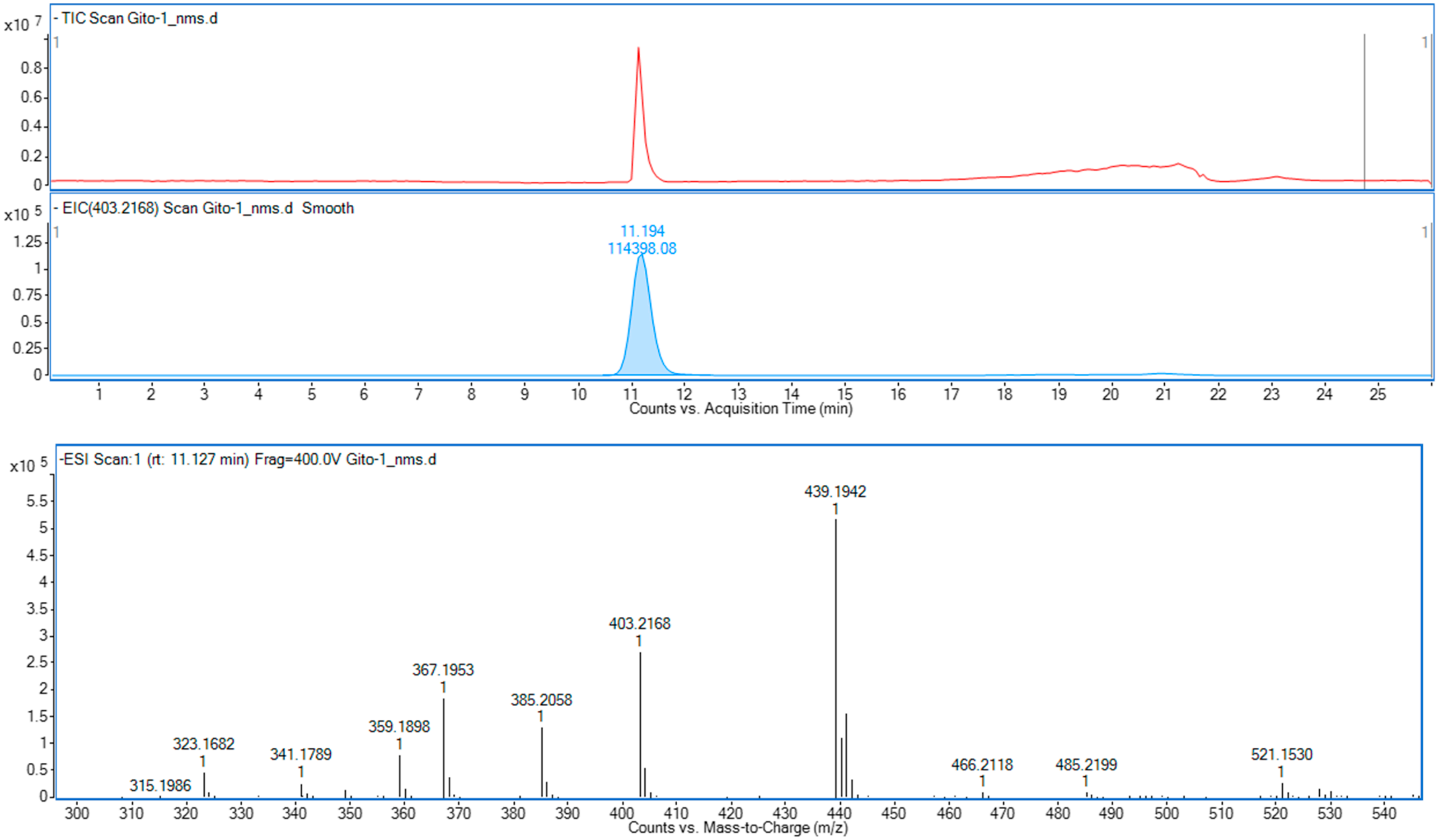

Chromatogram S6: LC-Q-ToF total and extracted ion chromatograms of **1** together with its mass spectrum (in negative mode).

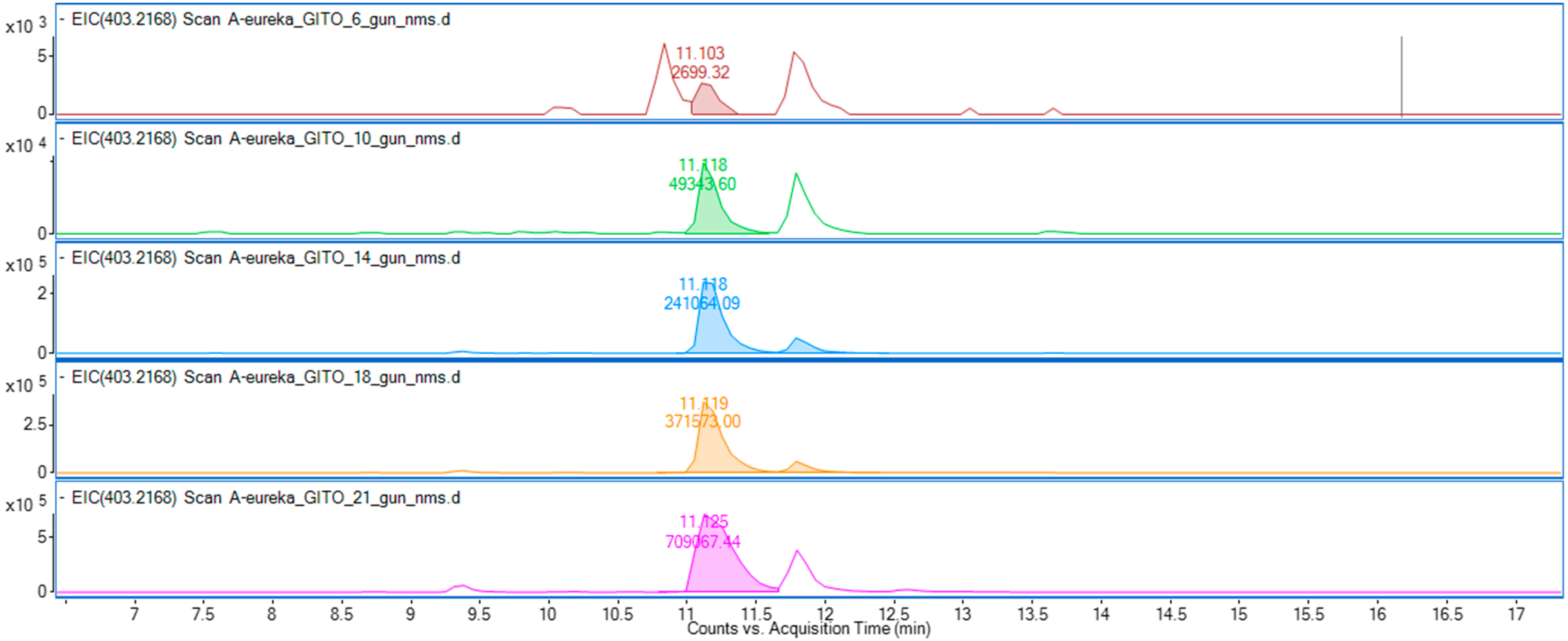

Chromatogram S7: LC-Q-ToF extracted ion chromatograms of **1** for different biotransformation time points (Day 6, 10, 14, 18 and 21), displaying that compound **1** was present and accumulated from Day 6 to Day 21 (in negative mode).

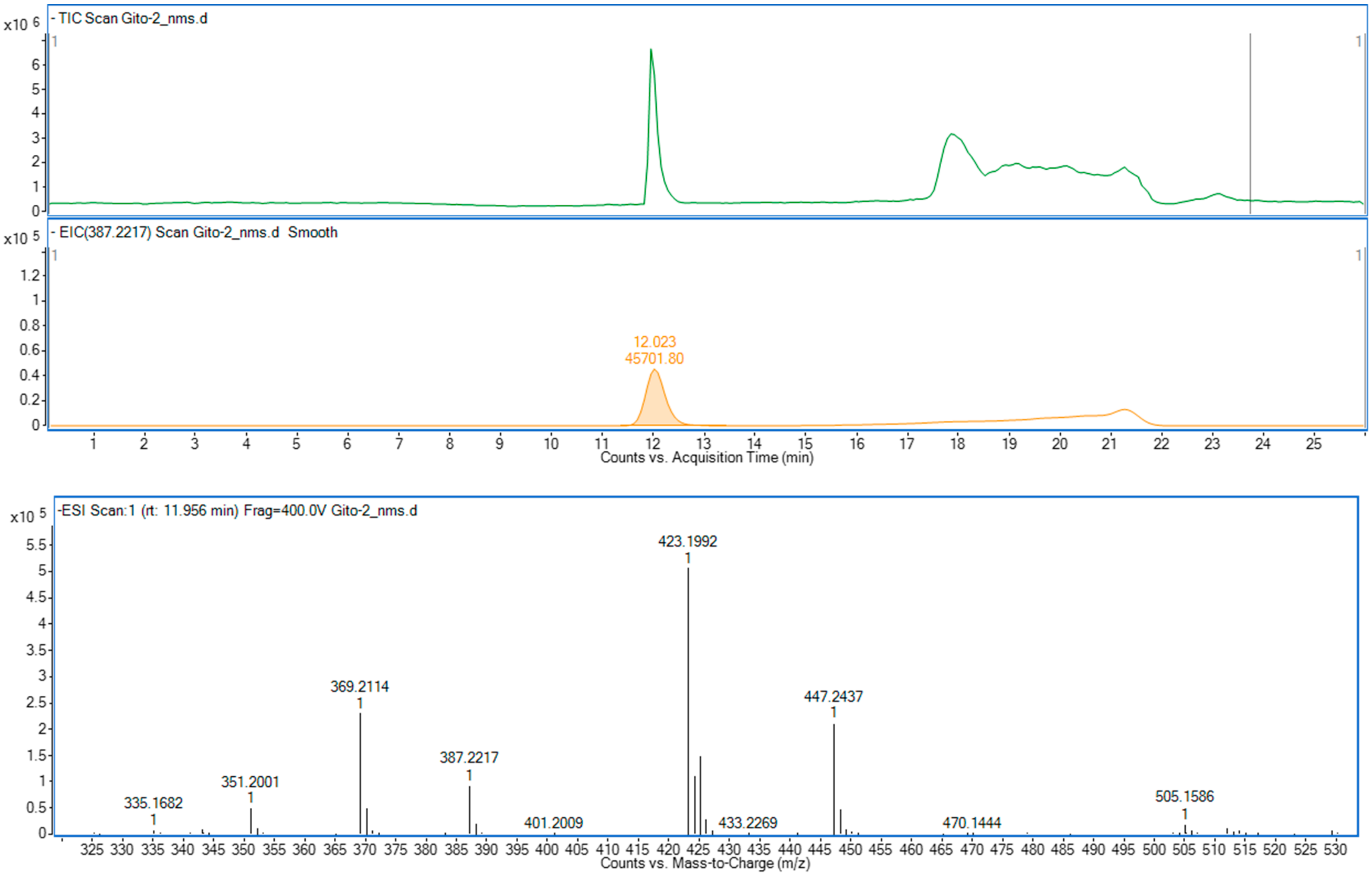

Chromatogram S8: LC-Q-ToF total and extracted ion chromatograms of **2** together with its mass spectrum (in negative mode).

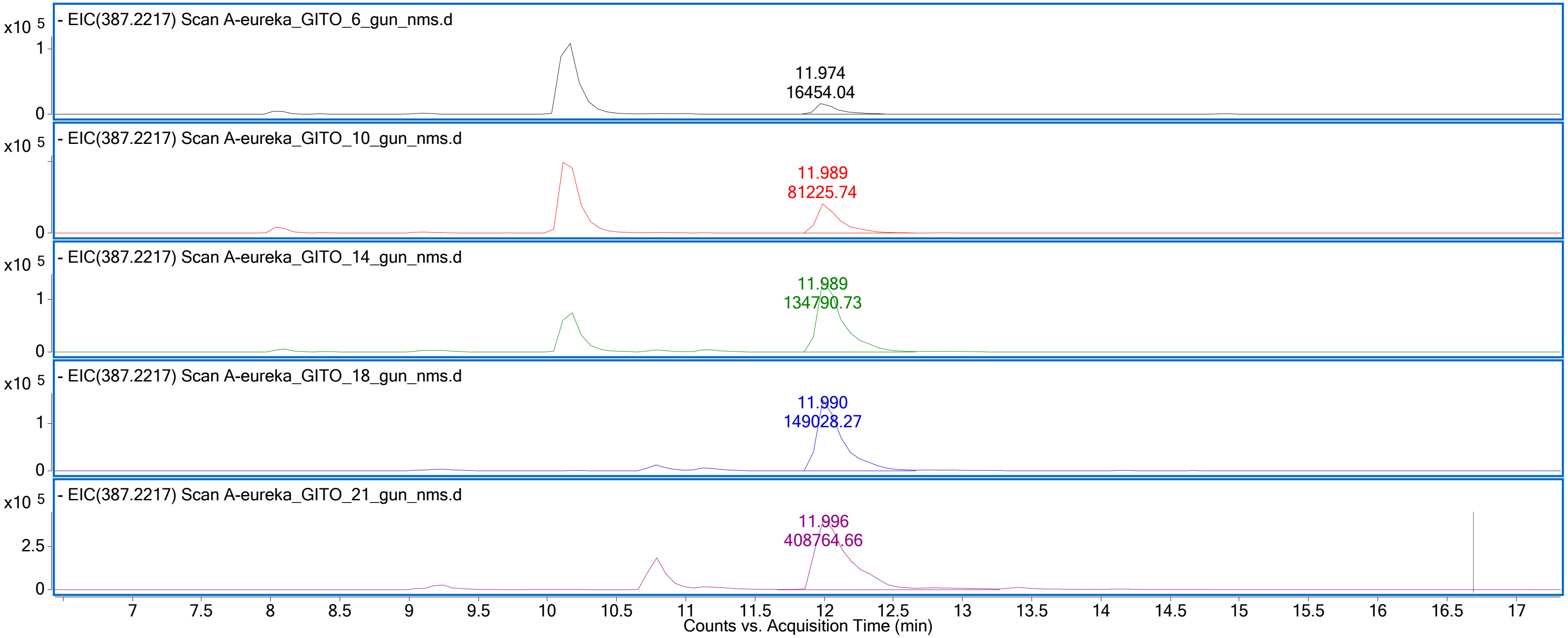

Chromatogram S9: LC-Q-ToF extracted ion chromatograms of **2** for different biotransformation time points (Day 6, 10, 14, 18 and 21), displaying that compound **2** was present and accumulated from Day 6 to Day 21 (in negative mode).

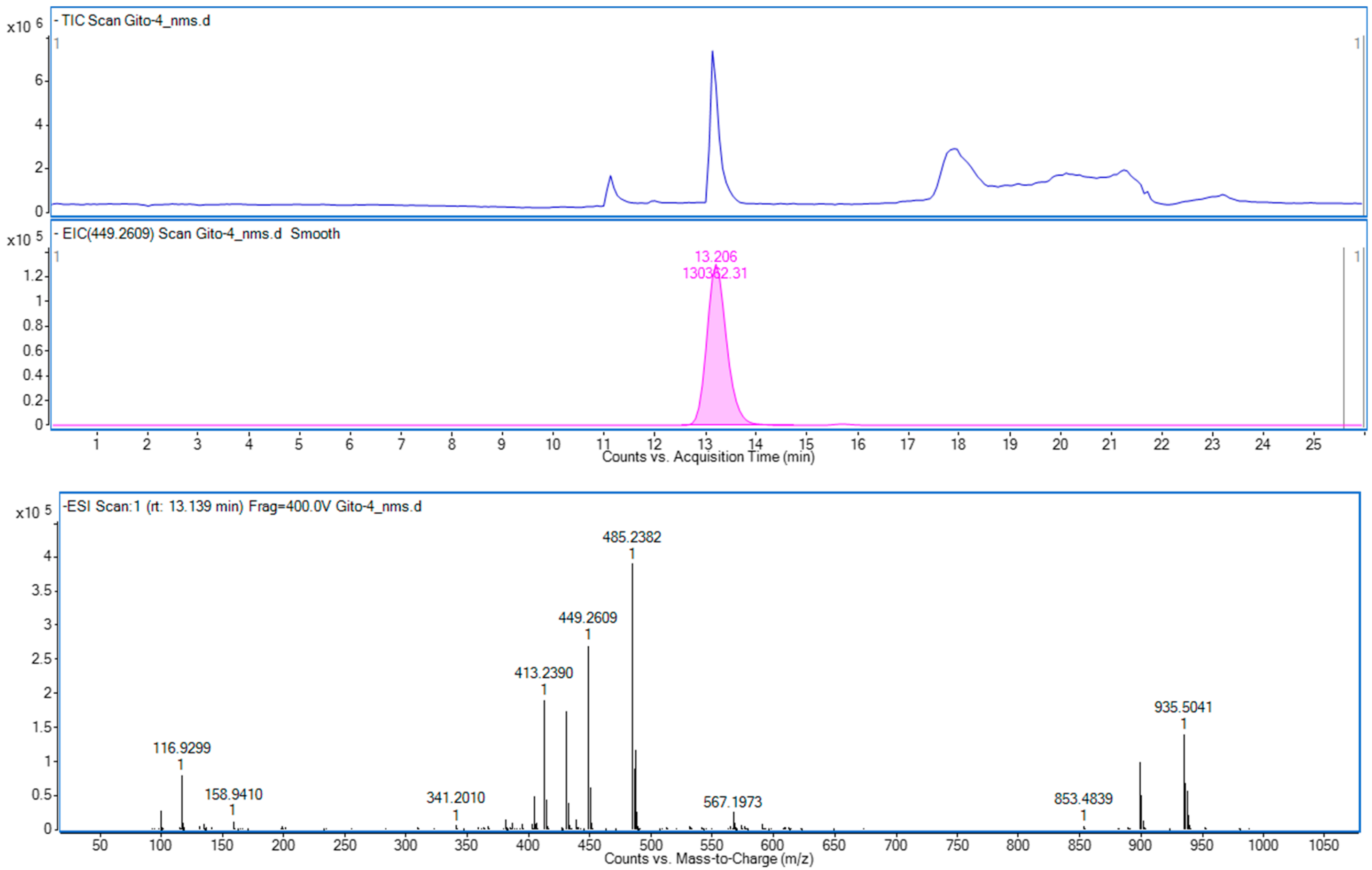

Chromatogram S10: LC-Q-ToF total and extracted ion chromatograms of **4** together with its mass spectrum (in negative mode).

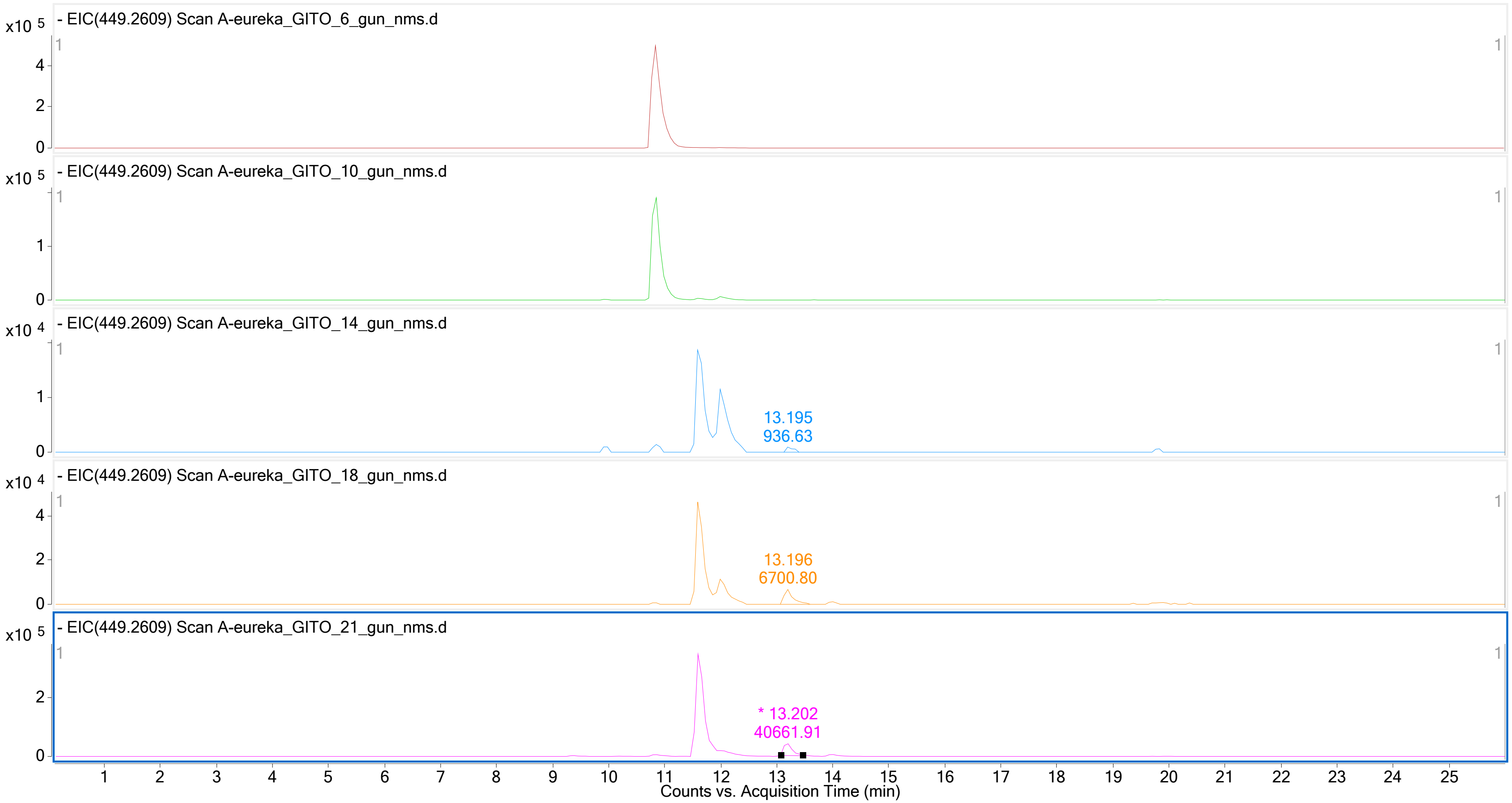

Chromatogram S11: LC-Q-ToF extracted ion chromatograms of **4** for different biotransformation time points (Day 6, 10, 14, 18 and 21), displaying that compound **4** was produced and accumulated in last three biotransformation time points (Day 14, 18 and 21) (in negative mode).

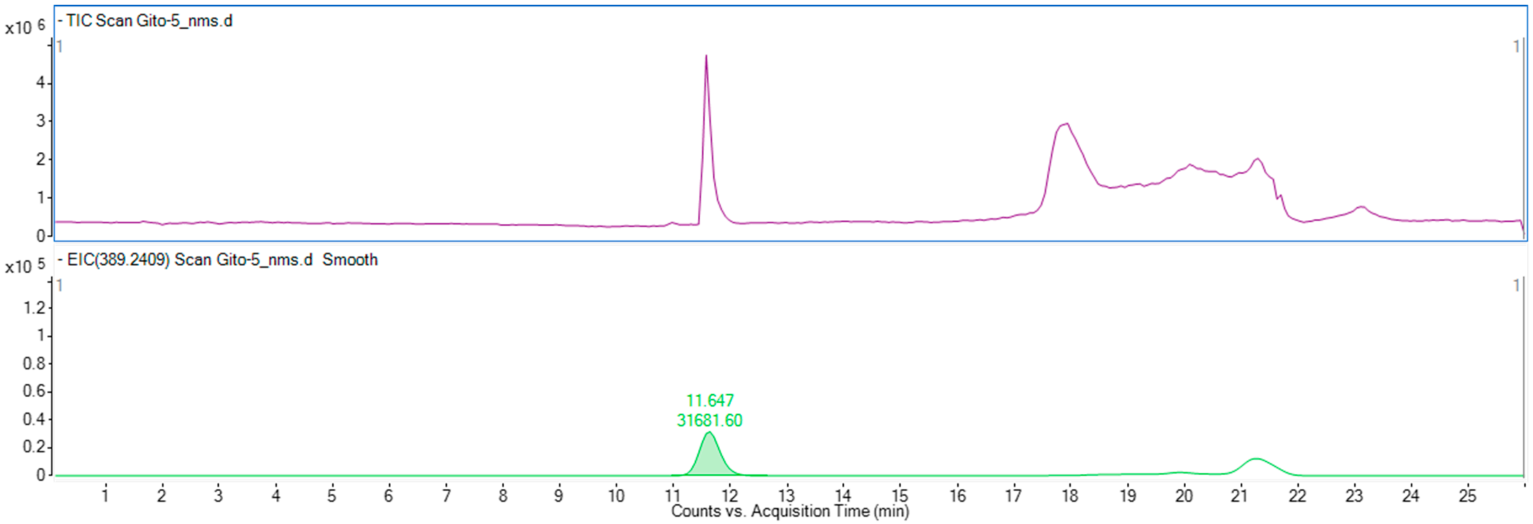

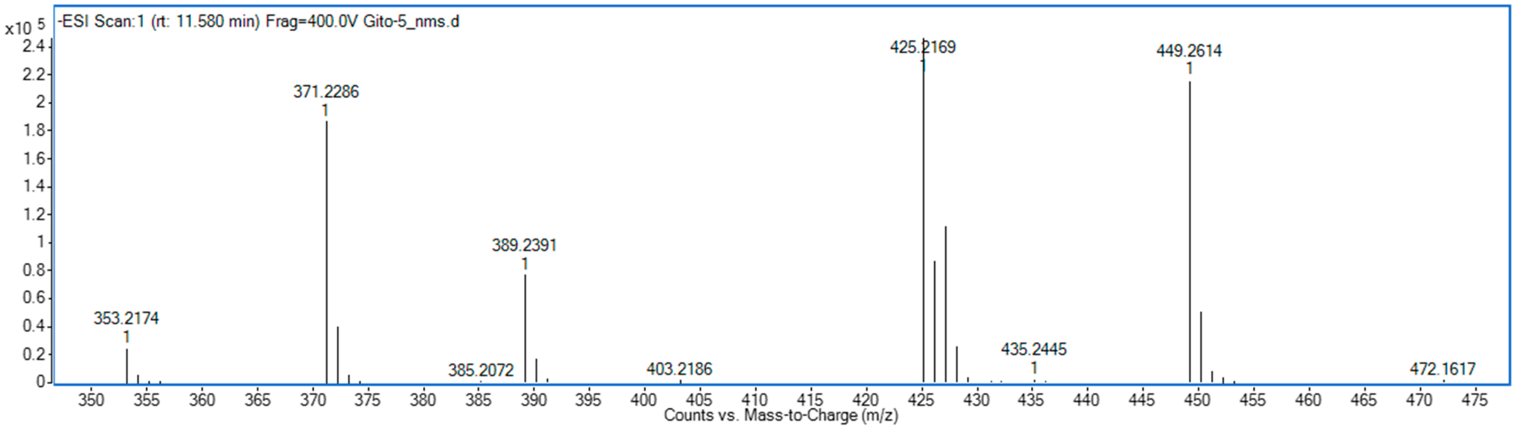

Chromatogram S12: LC-Q-ToF total and extracted ion chromatograms of **5** together with its mass spectrum (in negative mode).

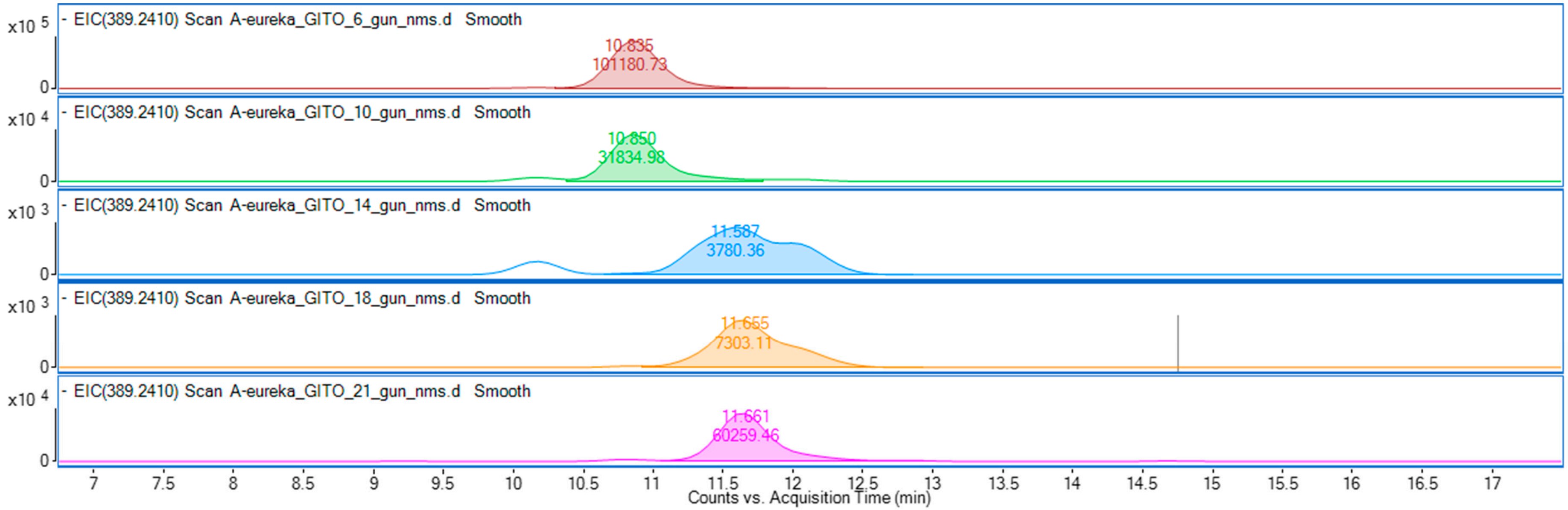

Chromatogram S13: LC-Q-ToF extracted ion chromatograms of **5** for different biotransformation time points (Day 6, 10, 14, 18 and 21), displaying that compound **5** was produced in the last three biotransformation time points (Day 14, 18 and 21) (in negative mode).

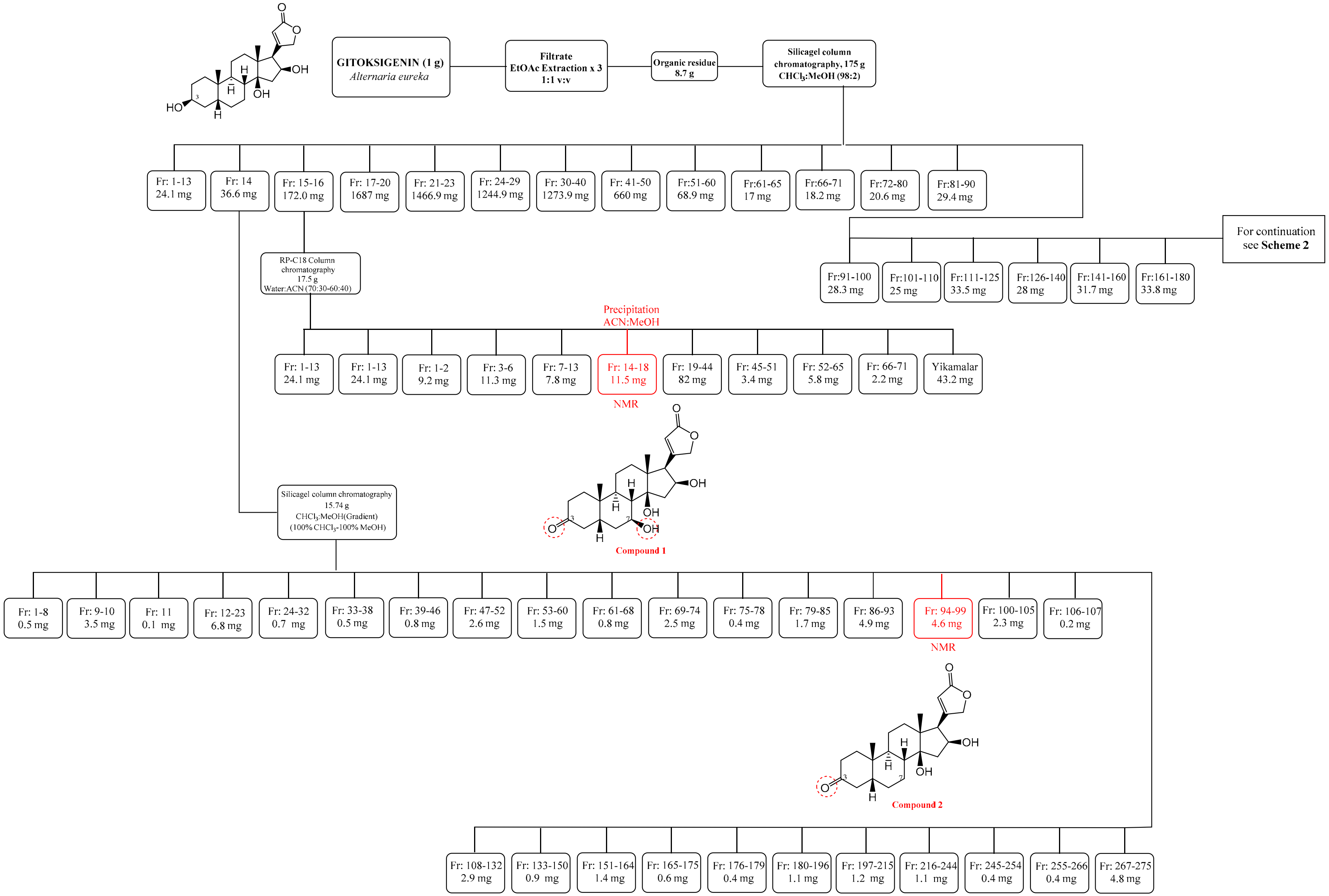

Scheme S1: Isolation pattern for biotransformation products of gitoxigenin

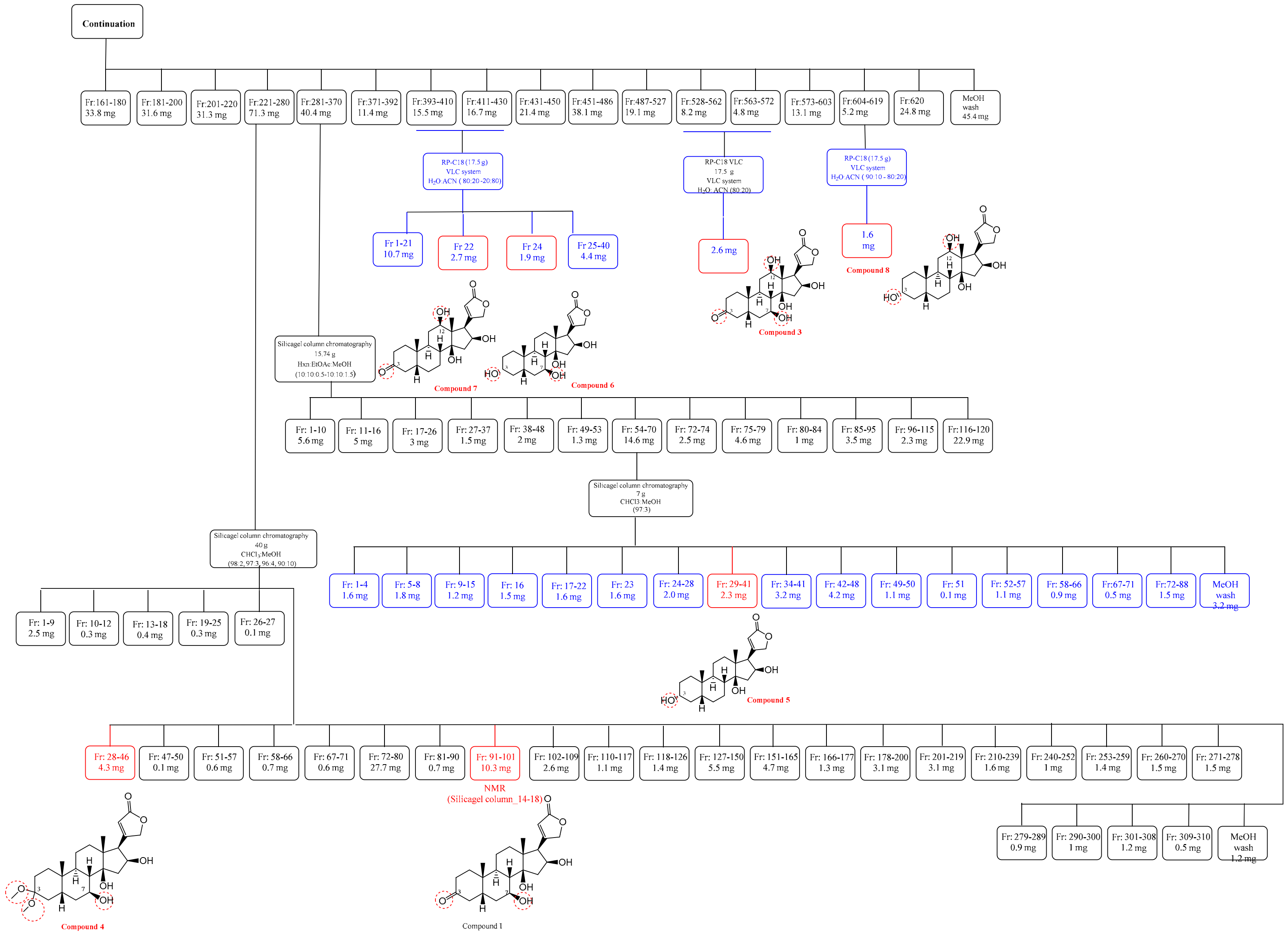

Scheme S2: Isolation pattern for biotransformation products of gitoxigenin

Table S2: LC-MS method for qualitative analysis of the selected metabolites (**1, 2, 4**, **5**), gitoxigenin, and the EtOAc extracts of biotransformation broth.

Column	Agilent Zorbax Extend column 4.6 mm x 150 mm x 3.5 µm
Column Temp.	25 ◦C
Injection volume	1 µl
Run Time	26 min.
Mobile Phase A	5mM Ammonium Acetate in Water
Mobile Phase B	Acetonitrile
Flow rate	0.6 ml/min
Gradient	0 min- 15 % B
	2 min- 15 % B
	12 min- 70 % B
	15 min-90 % B
	18 min- 90 % B
	18.1 min- 15 % B
	26 min- 15 % B
Ionisation mode	Negative electrospray ionisation mode with jet stream technology
Drying gas temp	200 ◦C
Drying gas flow	14 L/min
Nebuliser	35 psi
Sheath gas temp	400 ◦C
Sheat gas flow	11 L/min
Capillary Voltage	1500 V
Nozzle Voltage	1000 V
Mass range	40-1700 amu
Reference ions	980.0147, 1033.9881 for negative run
